# Recent advances in pyrazolo[3,4-*b*]pyridine chemistry: synthesis techniques and biological activity

**DOI:** 10.1007/s11030-025-11231-5

**Published:** 2025-06-25

**Authors:** Anhar Abdel-Aziem, Sawsan A. Fouad

**Affiliations:** Chemistry Department, Faculty of Science (Girls), Al-Azhar University, Nasr City, Cairo 11754 Egypt

**Keywords:** Pyrazolo[3,4-*b*] pyridine, Synthesis, Reactions, Biological activity

## Abstract

In recent years, attention has been drawn to pyrazolo[3,4-*b*] pyridine and its derivatives due to their substantial pharmacological activities such as antidepressant, anticancer, antiviral, antifungal, antioxidant, anticoagulant, anti-Alzheimer, and antimicrobial. Besides their medicinal uses, they act as herbicides, fungicides, and are also used as agrochemicals, inhibitors for a wide range of kinases, and fibroblast growth factor receptors. Additionally, pyrazolo[3,4-*b*] pyridines are used in developing organic materials for different applications such as chemosensors and corrosion inhibitors for mild steel. This review aims to provide an in-depth overview of the latest advancements regarding synthetic routes, reactions, and biological importance of pyrazolo [3,4-*b*] pyridine during 2019–2025 which will be a useful resource for interested researchers engaged in this area. The synthetic methodologies focus on metal-catalyst, nanocatalyst, microwave-assisted synthesis, multicomponent reactions, and other green chemistry approaches. Emphasizing the significance of these scaffolds in drug discovery and medicinal chemistry, new developments and future directions in the design of innovative pyrazolo[3,4-*b*]pyridine-based therapies are also covered.

**Graphical abstract:**

**Figure:**
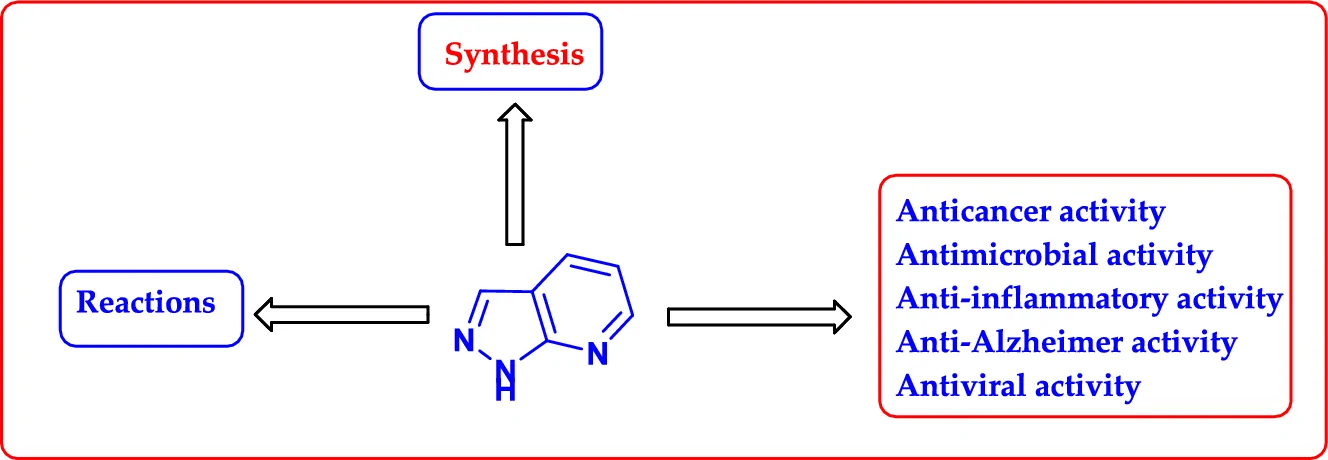

## Introduction

**Fig. 1:**
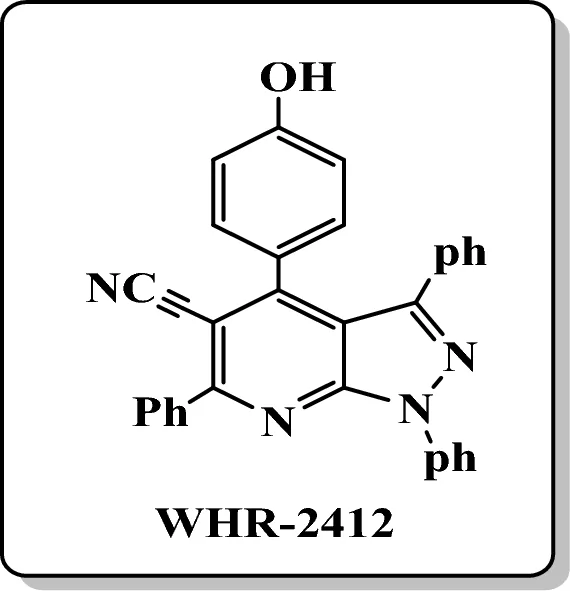
WRH-2412

Pyrazolopyridines are bicyclic heterocyclic compounds formed by fusion of two bioactive molecules pyrazole and pyridine rings [1–6] in different sites. Pyrazolo[3,4‐*b*]pyridine derivatives are fused *N*‐heterocycles that have been extensively studied because of their biological and pharmacological importance such as antileukemic [7], antihypertensive [8], anti-inflammatory [9–11], antiproliferative [12], antimicrobial [13–16] antioxidant [17], anti-Alzheimer [18–20], antiviral [21], anti-angiogenic [22], anticancer agents [23–27], and enzyme inhibitors [28–31]. It was reported that the novel synthetic **WRH-2412 (**4-(4-hydroxyphenyl)-1,3,6-triphenyl-1*H*-pyrazolo[3,4-*b*] pyridine-5-carbonitrile) exhibited antitumor activity in vitro [32] (Fig. 1).

Moreover, there are many drugs containing pyrazolo[3,4‐*b*]pyridine analogues such as tracazolate **I** (anxiolytic drug), amide derivative (**II**) (a potent inhibitor of glycogen synthase kinase-3 (GSK-3), and *N*-acylhydrazone (**III**) (analgesic) [33]. Moreover, Vericiguat V (Verquvo) and Riociguat IV (Adempas) were authorized as therapies for cardiovascular diseases and brought to mark

**Fig. 2:**
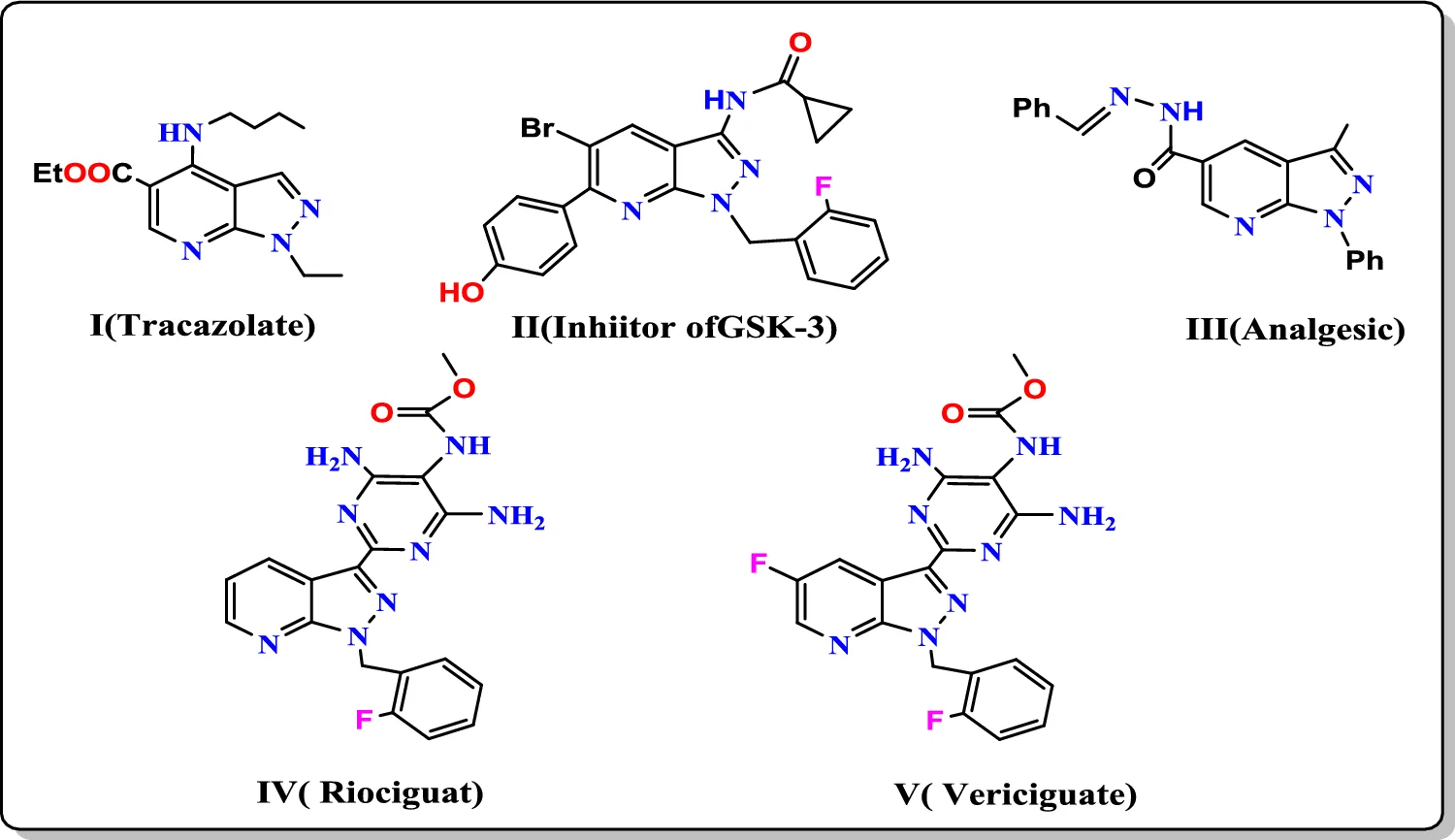
Drugs based on functionalized pyrazolo[3,4-*b*]pyridines

et in 2013 and 2021 [34], respectively (Fig. 2).

Furthermore, several pyrazolo[3,4‐*b*]pyridine analogues have been reported as p38a kinase inhibitors and inhibited CDK2 and CDK9.

### Chemistry of pyrazolo[3,4-b]pyridine

**Fig. 3:**
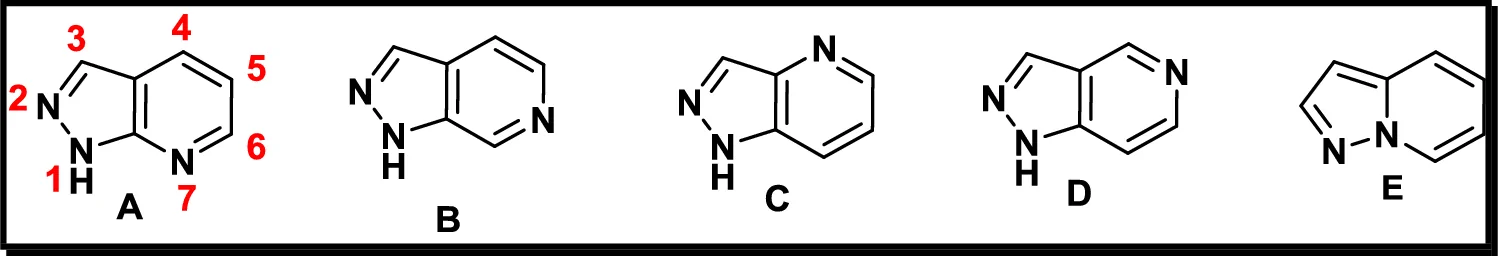
Types of pyrazolopyridine

There are five possible structures resulting from fusion of the two rings: pyrazolo[3,4-*b*] **A**, [3,4-*c*] **B**, [4,3-*c*] **C**, [4,3-*b*] **D**, and [1,5-*a*] **E** pyridine (Fig. 3).

**Fig. 4:**
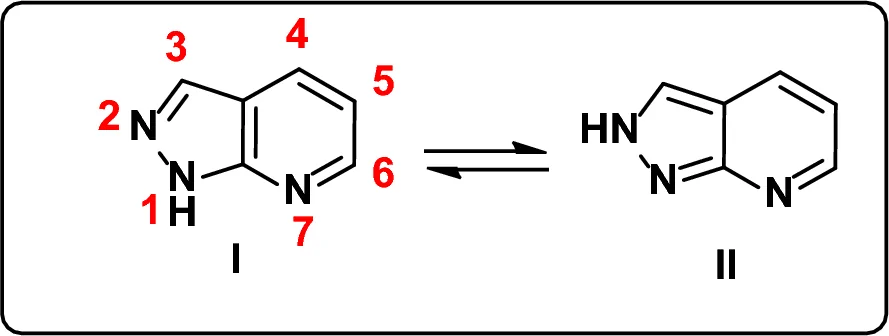
Tautomeric 1*H*-pyrazolo[3,4-*b*]pyridine (I) and 2*H*-pyrazolo[3,4-*b*]pyridine (II)

Pyrazolo[3,4-*b*]pyridine can be found in two isomeric structures: 1*H*-pyrazolo [3,4-*b*]pyridines and 2*H*-pyrazolo[3,4-*b*]pyridines (Fig. 4). Because there is little research on 2*H*-pyrazolo[3,4–*b*] pyridines, we have decided to restrict the current review to the structural, synthesis, reactions, and biological properties of 1*H*-pyrazolo[3,4–*b*]pyridines A, with a focus on the years 2019–2025.

### Synthesis of 1*H*-pyrazolo[3,4-*b*]pyridines

**Fig. 5:**
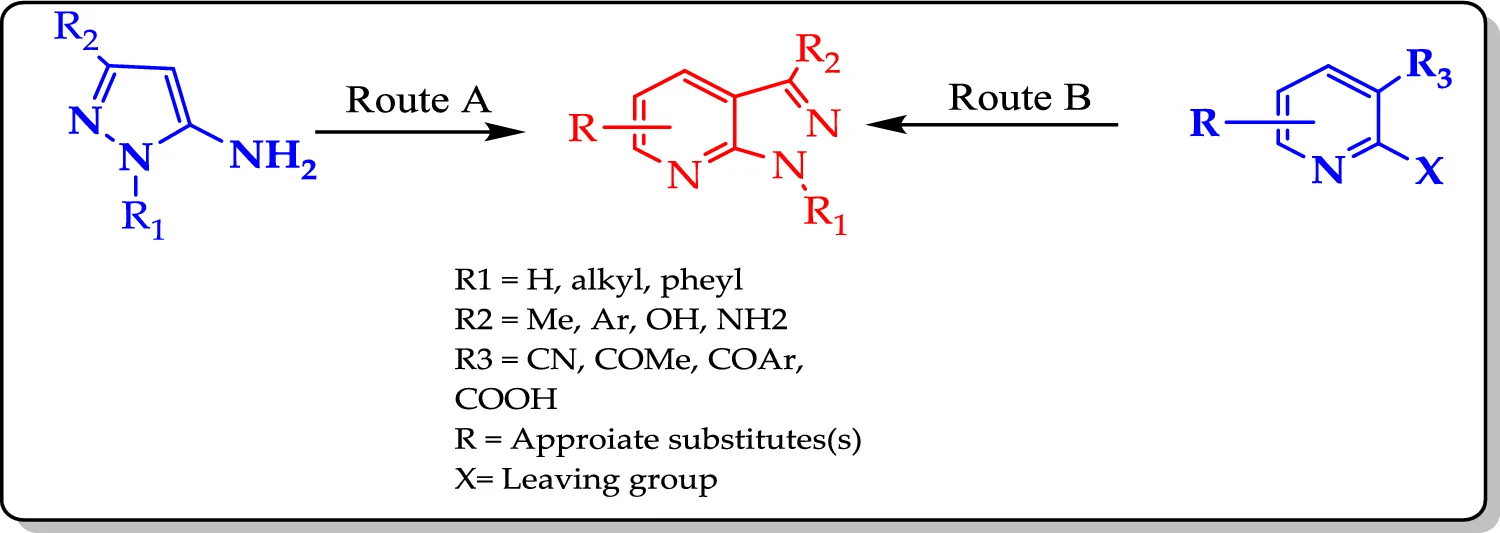
Graphical abstract of pyrazolo[3,4-*b*]pyridines derivatives

Several researchers used various catalysts, solvents, media, or physical conditions to develop novel synthetic approaches for pyrazolo[3,4-*b*]pyridines. Green synthesis and multicomponent reaction are also a better approach for pyrazolo[3,4-*b*]pyridines. In this review, the synthetic methodologies for pyrazolo[3,4-*b*]pyridines can be categorized into two principal segments: formation of pyridine onto an existing pyrazole ring (rout A) and formation of pyrazole onto an existing pyridine ring (rout B) [35] (Fig. 5).

### Formation of pyrazole onto an existing pyridine ring

**Scheme 1:**
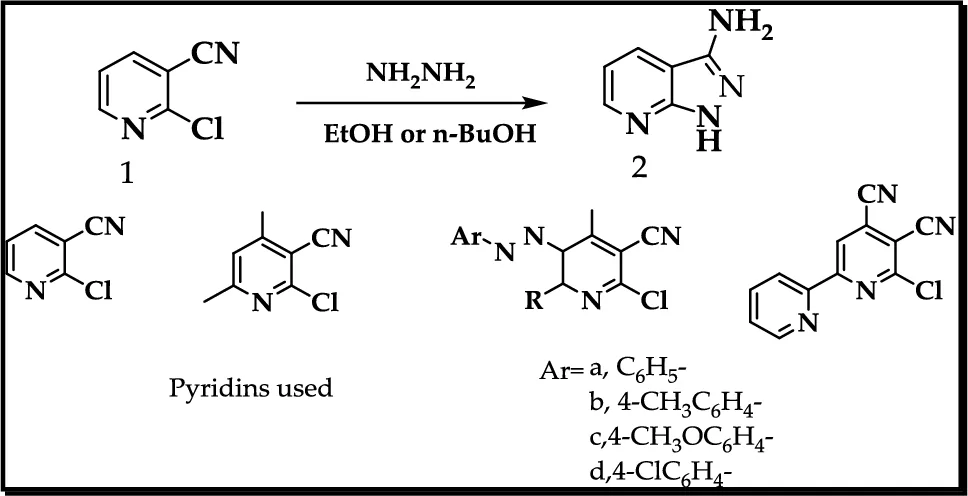
Synthesis of 3-amino-1*H*-pyrazolo[3,4-*b*]pyridines 2 using 2-chloro-3-cyano pyridines

Pyrazolo[3,4-*b*]pyridines can be produced starting with pyridine derivative and closing the pyrazole ring. This was achieved by the reaction of pyridine ring bearing chlorine atom and an electrophilic group such as (CN, Ketone, aldehyde, ester) with hydrazine hydrate or its derivatives. Many authors synthesized 3-amino-1*H*-pyrazolo [3,4-*b*]pyridines **2** via reaction of 2-chloro-3-cyano-pyridine derivatives **1** with hydrazine hydrate using ethylene glycol at 165 °C [36], butanol [37] , or ethanol [14, 38] as solvents. Arafa and Hussein [39] carried out the reaction under irradiation at 50 °C for 15 min and with 96% yield (Scheme 1).

#### The reaction mechanism of pyridine with hydrazine

**Scheme 2:**
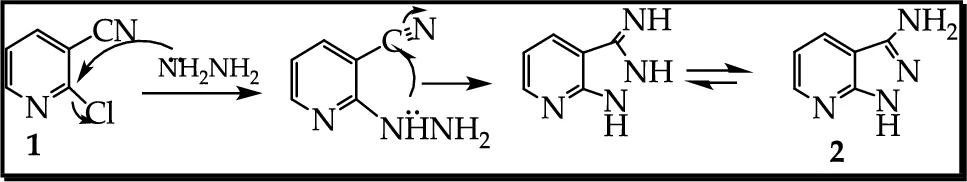
Mechanism for the reaction of 2-chloro-3-cyanopyridine with hydrazine hydrate

The reaction begins with the nucleophilic attack of the hydrazine to both electrophilic positions of the pyridine ring (chloro substituted carbon and the electrophilic group at position C3 followed by tautomerization (in the case of a C3 nitrile or ester group) or by elimination of water (in the case of aldehydes or ketones). Likewise, hydrazine hydrate easily replaced the chloro atom in derivative **1a** followed by internal nucleophilic attack on the adjacent cyano group to finally afford compound **2** (Scheme 2).

**Scheme 3:**
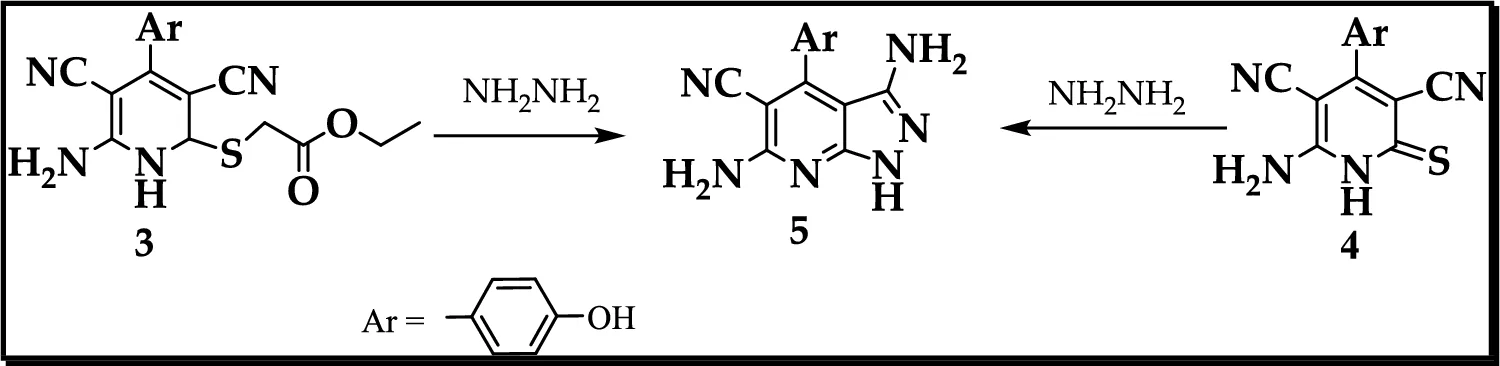
Reaction of cyano pyridine derivatives with hydrazine hydrate

On the other hand, Sanad et al. [40] reported the reaction of ethylpyridine-2(1*H*)-thioacetate **3** or pyridine-2(1*H*)-thione derivative **4** with hydrazine hydrate in boiling ethanol gave pyrazolo [3,4-*b*]pyridine **5** (Scheme 3).

**Scheme 4:**
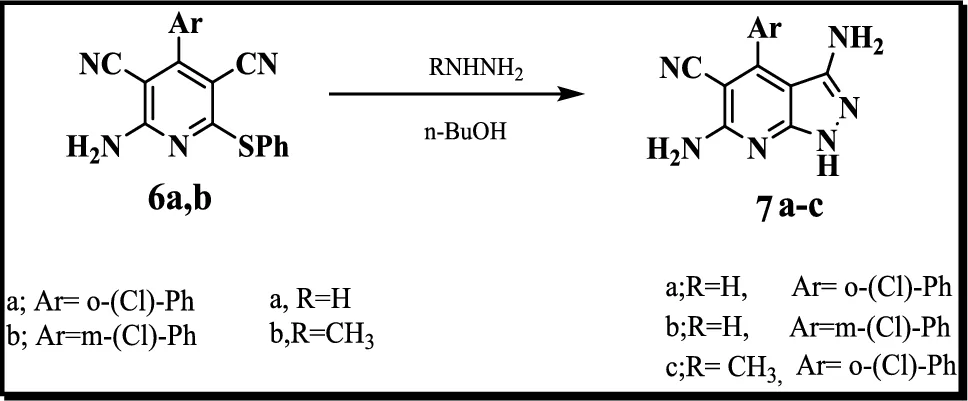
Reaction of cyano pyridines 6a,b with hydrazine hydrate derivatives

Also, Nafie et al. [41] reported the synthesis of 3,6-diamino-4-aryl-pyrazolo[3,4-*b*]pyridine-5-carbonitrile derivatives **7a-c** by the reaction of cyanopyridin derivative **6a,b** with hydrazine hydrate and/or methyl hydrazine (Scheme 4).

**Scheme 5:**
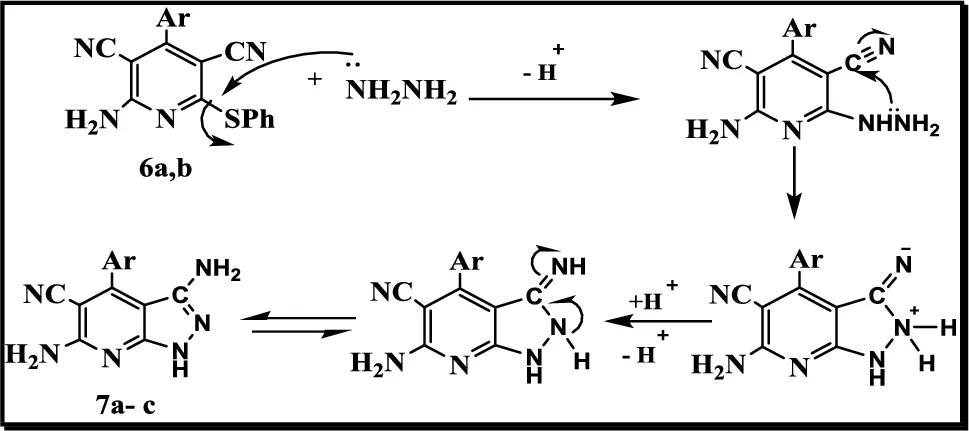
Mechanism for the reaction of pyridines 6a,b with hydrazine hydrate

The formation of compounds **7a-c** can be explained via nucleophilic addition of NH_2_ to electrophilic carbone followed by elimination of thiophenol and cyclization according to the following mechanism (Scheme 5).

**Scheme 6:**
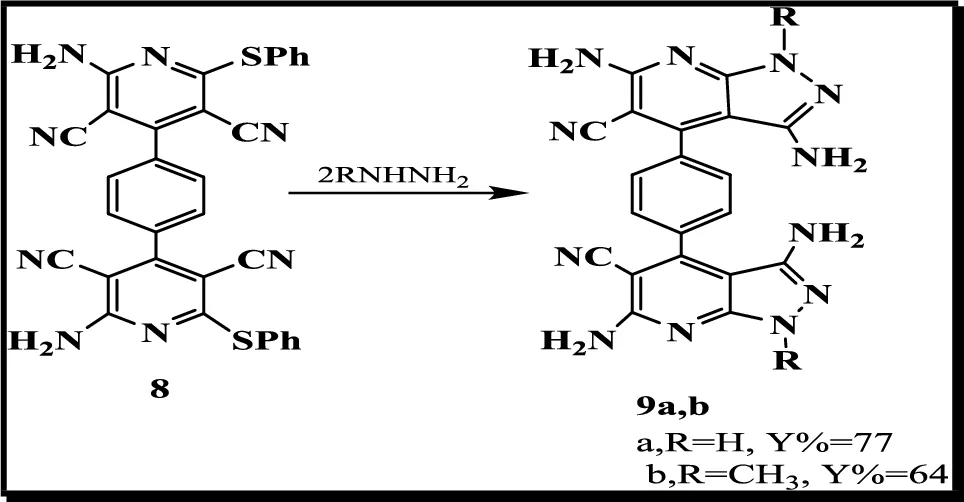
Cyclization of 4,4′-(1,4-phenylene)bis(2-amino-6-(phenylthio)pyridine-3,5-dicarbonitrile 8

However, 4,4′-(1,4-phenylene)bis(2-amino-6-(phenylthio)pyridine-3,5- dicarbonitrile **8** underwent cyclization with hydrazine hydrate or methyl hydrazine gave pyrazolo[3,4-*b*]pyridine-5-carbonitrile **9a,b** via elimination of two moles from thiophenol [41] (Scheme 6).

**Scheme 7:**
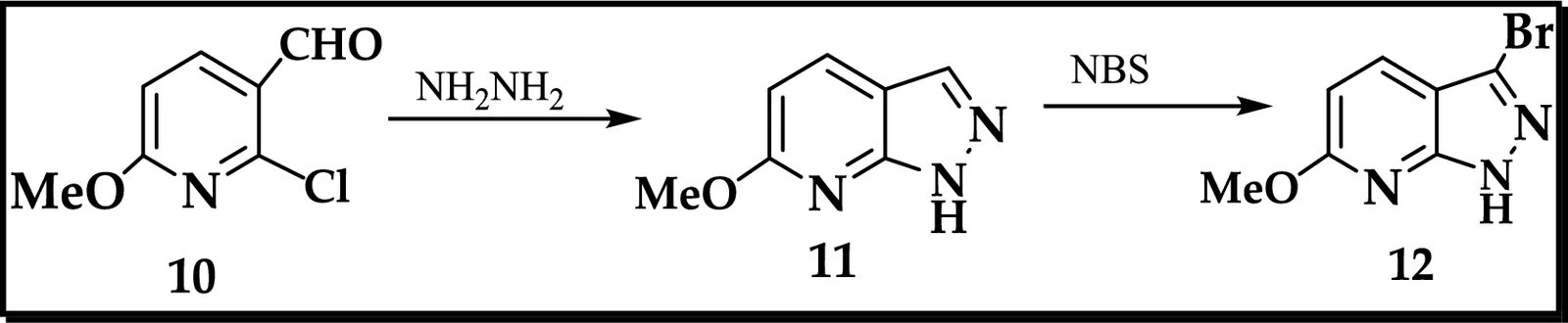
Synthesis of 3-bromo-6-methoxy-1*H*-pyrazolo[3,4-*b*]pyridine 12

3-Bromo-6-methoxy-1*H*-pyrazolo[3,4-*b*]pyridine **12** was developed by Hao *et al* [22]. Initially, 2-chloro-6 methoxy nicotinylaldehyde **10** was reacted with hydrazine hydrate under refluxing conditions produced 6-methoxy-1*H*-pyrazolo[3,4-*b*] pyridine **11**. The subsequent bromination of **11** at the 3-position with **NBS(**N-bromosuccinamide**)** generated **12** (Scheme 7).

**Scheme 8:**
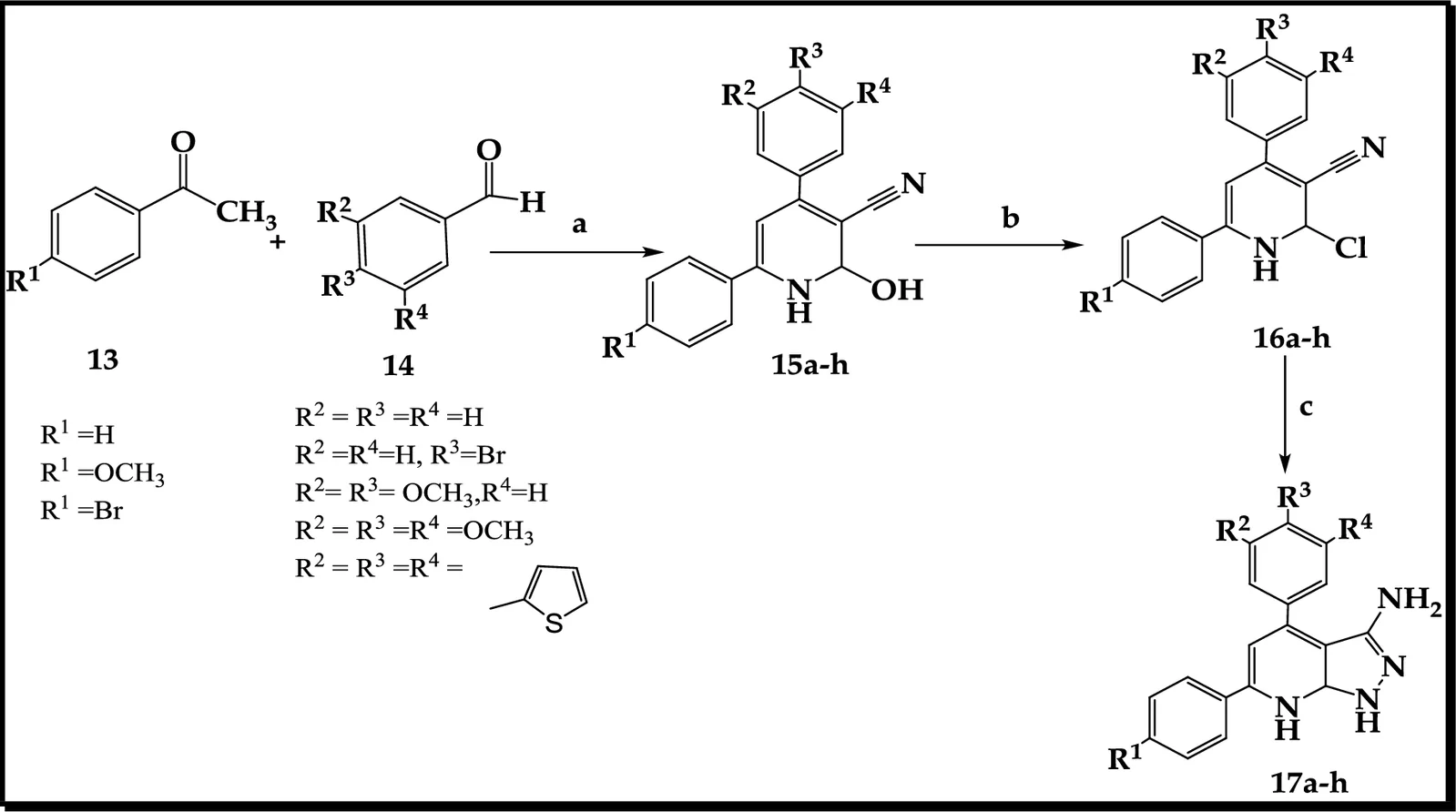
Synthesis of 1*H*-pyrazolo[3,4-*b*]pyridin-3-amine derivatives 17a-h Reagents and conditions: (**a**) ammonium acetate, ethyl cyanoacetate; ethanol, reflux, 3 h (70–77%); (**b**) POCl_3_; reflux, 15 h (75–88%); (**c**) NH_2_NH_2_.H_2_O; ethanol/ DMF, reflux, 24–48 h (79–88%)

1*H*-Pyrazolo[3,4-*b*]pyridin-3-amine derivatives 1**7a-h** were developed via chlorination of 1**5a-h** with POCl_3_ to afford the corresponding 2-chloronicotinonitrile 1**6a-h** followed by condensation with hydrazine hydrate in ethanol/DMF [20, 42] (Scheme 8).

**Scheme 9:**
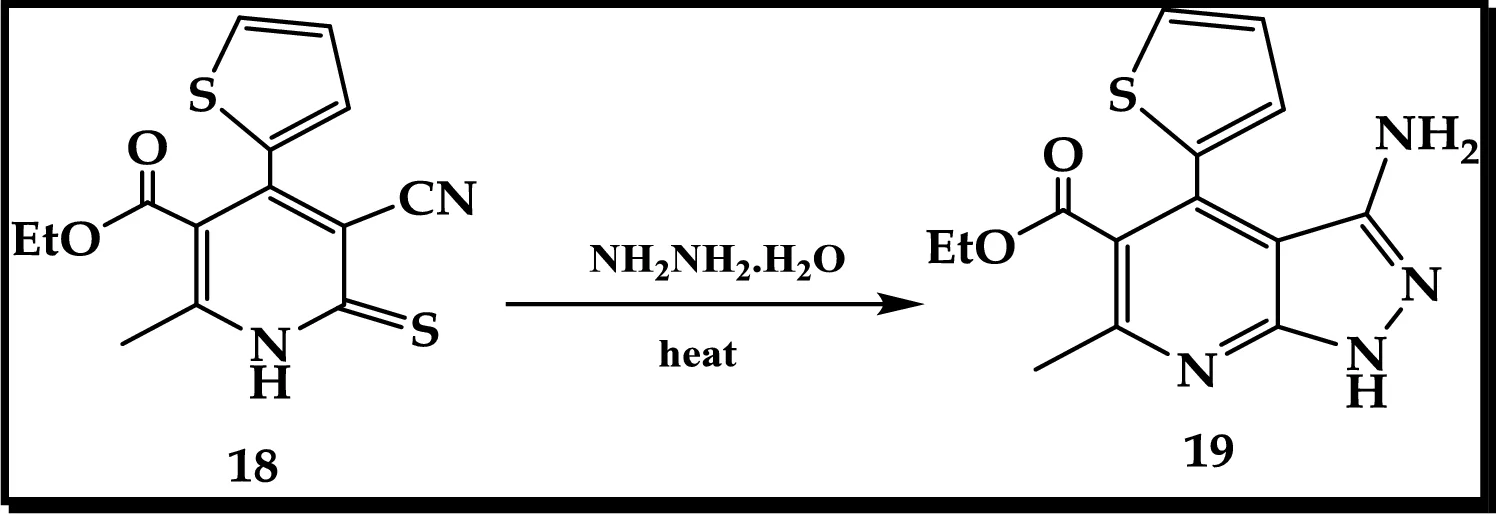
Synthesis of ethyl 3-amino-6-methyl-4-(thiophen-2-yl)-1*H*-pyrazolo[3,4- *b*]pyridine-5-carboxylate 19

Also, El Bakri et al*.* [43] prepared ethyl 3-amino-6-methyl-4-(thiophen-2-yl)-1*H*-pyrazolo[3,4-*b*]pyridine-5-carboxylate **19** in a pure state and a good yield via the reaction of ethyl 5-cyano-2-methyl-4-(thiophen-2-yl)- 6-thioxo-1,6 dihydro pyridine-3-carboxylate **18** with an excess amount of hydrazine hydrate in a steam bath for five hours in the absence of any solvent (Scheme 9).

**Scheme 10:**
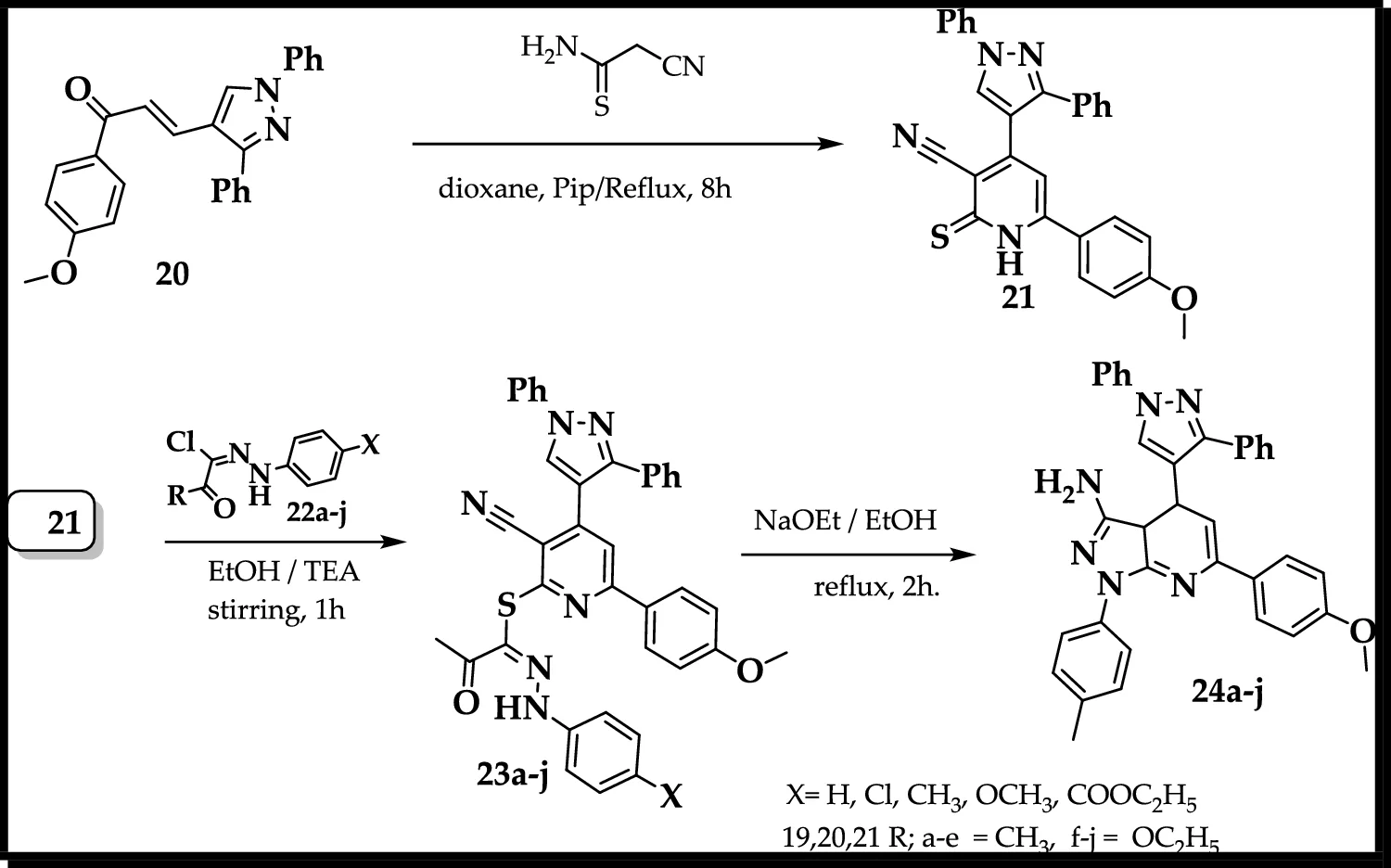
Formation of 1*H*-pyrazolo[3,4-*b*]pyridines 24 a-j from the reaction of α, β- unsaturated ketones 20 with cyanothioacetamide

α, β-Unsaturated ketones **20** reacted as 1,3-biselectrophiles with cyanothio acetamide in dioxane/piperidine yielded substituted 2-thioxo-1,2-dihydro pyridine-3-carbonitrile derivative **21**. The reaction of the latter with hydrazonoyl halides **22a-j** in hot ethanol using TEA as a catalyst afforded the corresponding pyridin-2-yl-2-oxo-*N* phenyl propane hydrazonothioate derivatives **23a-j** in excellent yields (90–96%). Heating of compound **23a-j** with ethanolic sodium ethoxide led to rearrangement and cyclization giving pyrazolo[3,4-*b*]pyridin-3-amines **24a-j** [44] (Scheme 10).

**Scheme 11:**
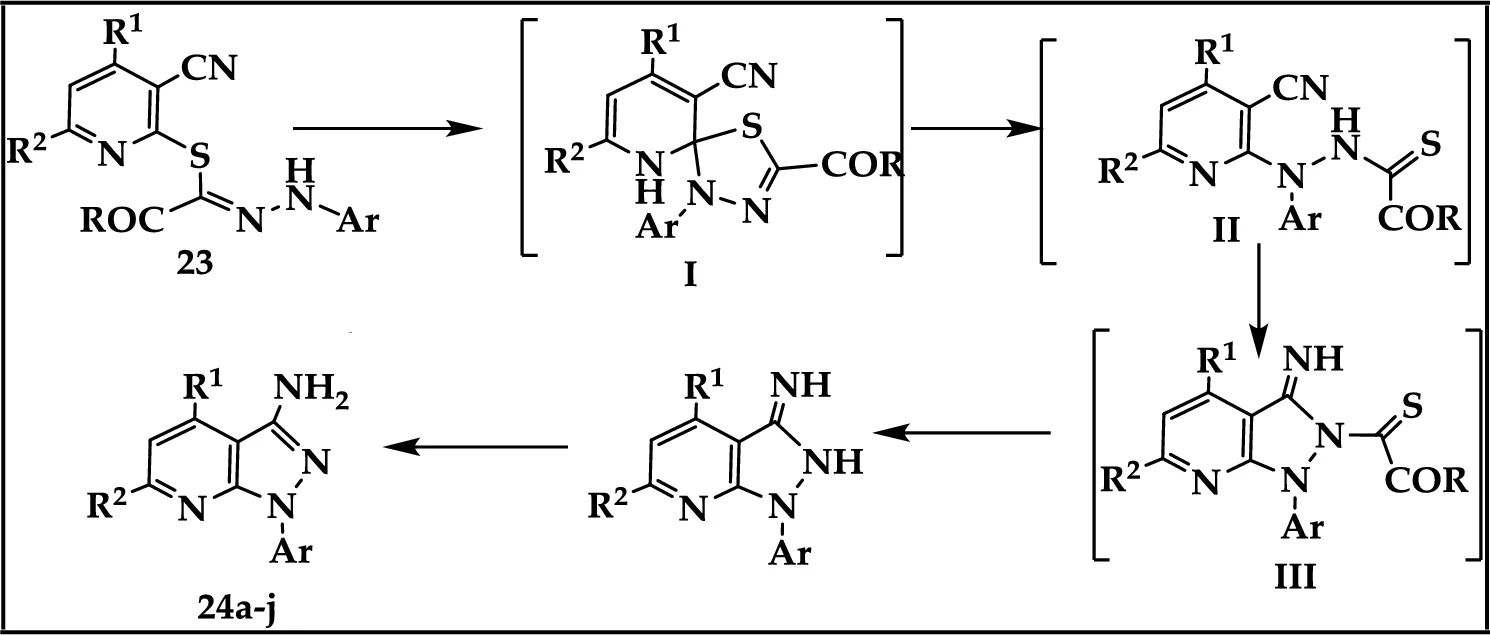
The suggested mechanism for the formation of pyrazolo[3,4-*b*]pyridin- 3-amines 24a-j

The suggested mechanism that explains the formation of pyrazolo[3,4-*b*]pyridin-3-amines **24** is illustrated in Scheme 11. The formation of the product follows two steps namely (a) Smiles rearrangement of the initially thiohydrazonates **23** to yield the thiohydrazides II and (b) cyclization of the latter give the pyrazolo[3,4-*b*]pyridine-3-amines **24a-j** as end-products.

### Formation of pyridine ring onto an existing pyrazole

#### Two-component reactions

Pyrazolo[3,4-*b*]pyridines can be produced starting with 5-aminopyrazole derivative and closing the pyridine ring. The amino pyrazoles will act as 1,3-NCC-dineucleophil and react with 1,3-CCC-biselectrophile (active methylene).

**Scheme 12:**
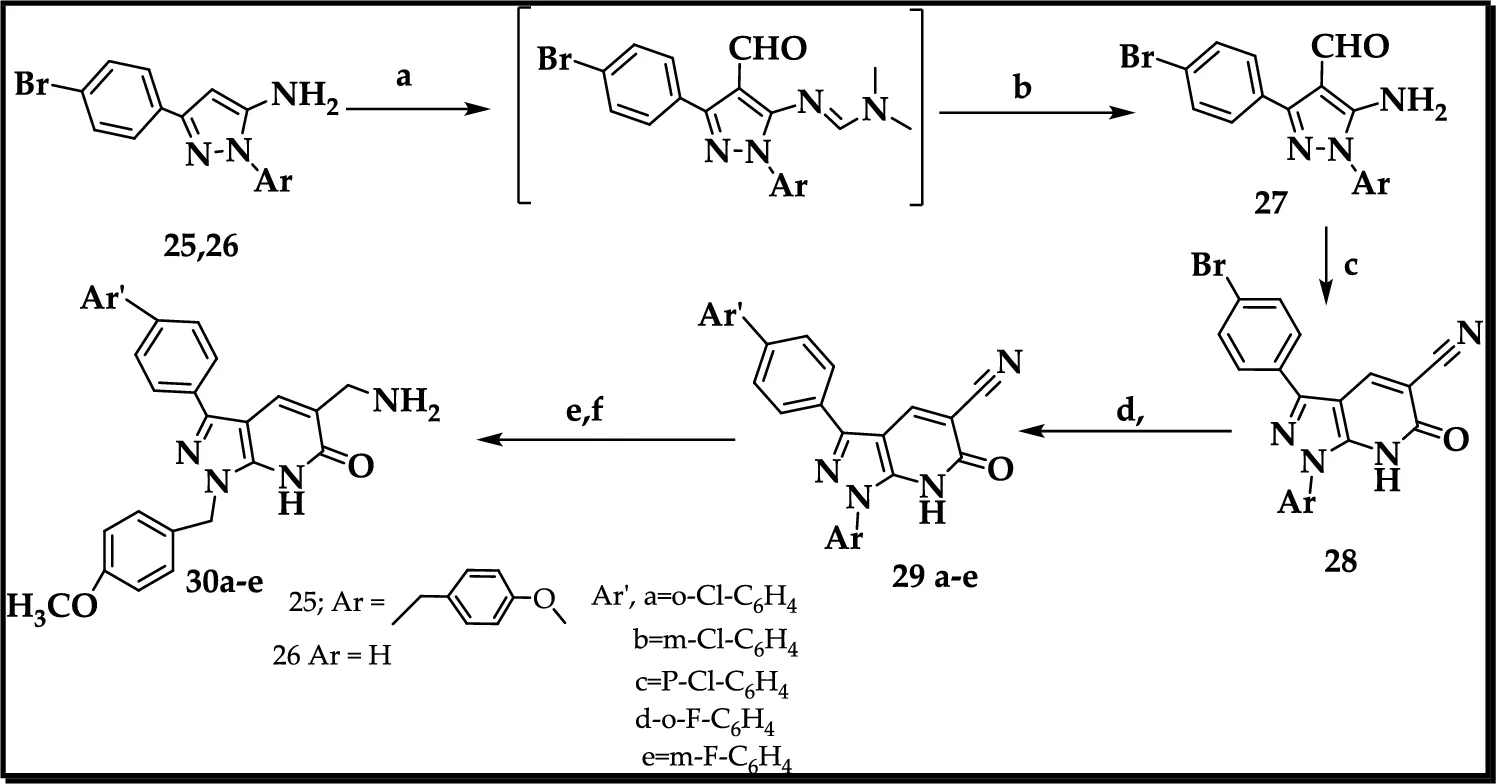
Friedlander reaction for synthesis of pyrazolo[3,4‐*b*]pyridine. Reagents and conditions: **a**. DMF, POCl_3_, 100 °C, 3 h; **b**. EtOH/H_2_O, NaOH, reflux, 2 h; **c**. CNCH_2_COOEt, EtOH, piperidine, reflux, 8 h; **d**. substituted‐phenylboronic acid, PdCl_2,_ CH_2_Cl_2_,K_2_CO_3_,H_2_O, dioxane, 100 °C,18 h; **e**. NaBH_4_, COCl_2_·6H_2_O, (Boc)_2_O,CH_3_OH, rt, 3 h; **f**. CH_2_Cl_2_,CF_3_COOH, rt,3 h

Zheng et al. [45] developed new pyrazolo[3,4‐*b*]pyridine derivatives **30a-e** by subjecting compounds **25, 26** to Vilsmeier–Haack–Arnold reaction to provide compound **27**, which was followed by Friedlander reaction to produce pyrazolo[3,4‐*b*]pyridine **28**. Suzuki–Miyaura cross‐coupling of **28** was carried out with substituted‐phenylboronic acid at 100 °C to provide the corresponding products **29a-d**. Cyano group in the structure of **29a-d** were further reduced by NaBH_4_/Boc_2_O to give Boc‐protected amines, which underwent deprotection reaction by TFA at room temperature to provide compounds **30a-d** (Scheme 12).

**Scheme 13:**
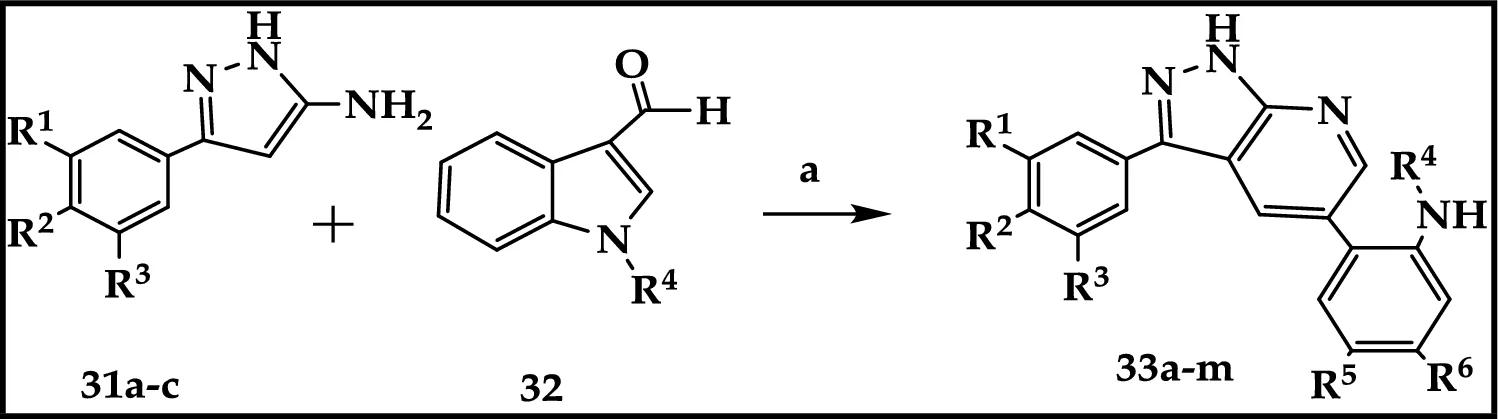
Synthesis of 1*H*-pyrazolo[3,4-*b*]pyridines from amino pyrazole and indolaldehydes. Reagents and condition: (b) AlCl_3_, MeOH, 80 (b), 4–8 h

Zhai et al. [46] demonstrated the synthesis of the target compounds **33a-m** from the reaction of compounds **31a-c** with indole-3-carboxaldehyde derivatives **32** (Scheme 13).

**Scheme 14:**
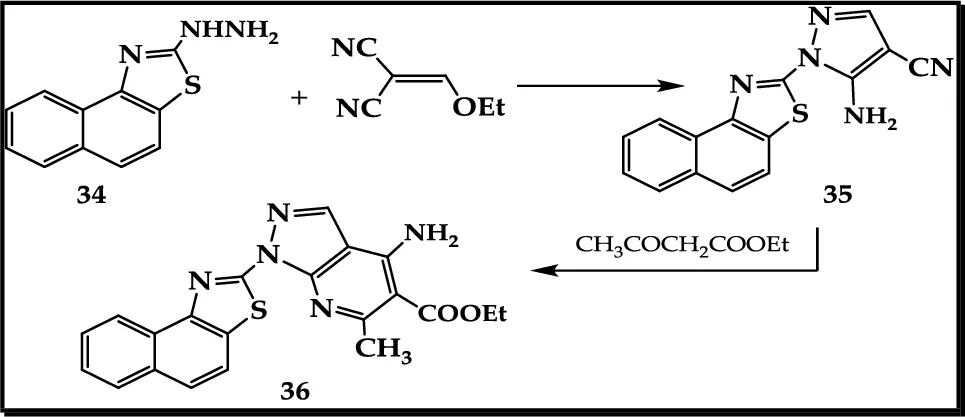
Synthesis of 1*H*-pyrazolo[3,4-*b*]pyridine 36

Fayed et al. [47] prepared 4-aminopyrazolo[3,4-*b*]pyridine derivative **36** via the reaction of compound **34** with ethoxy methylene malononitrile that afforded the corresponding 3-amino-4-cyano pyrazole derivative **35**, which was reacted with ethylacetoacetate to provide the corresponding product **36** (Scheme 14).

**Scheme 15:**
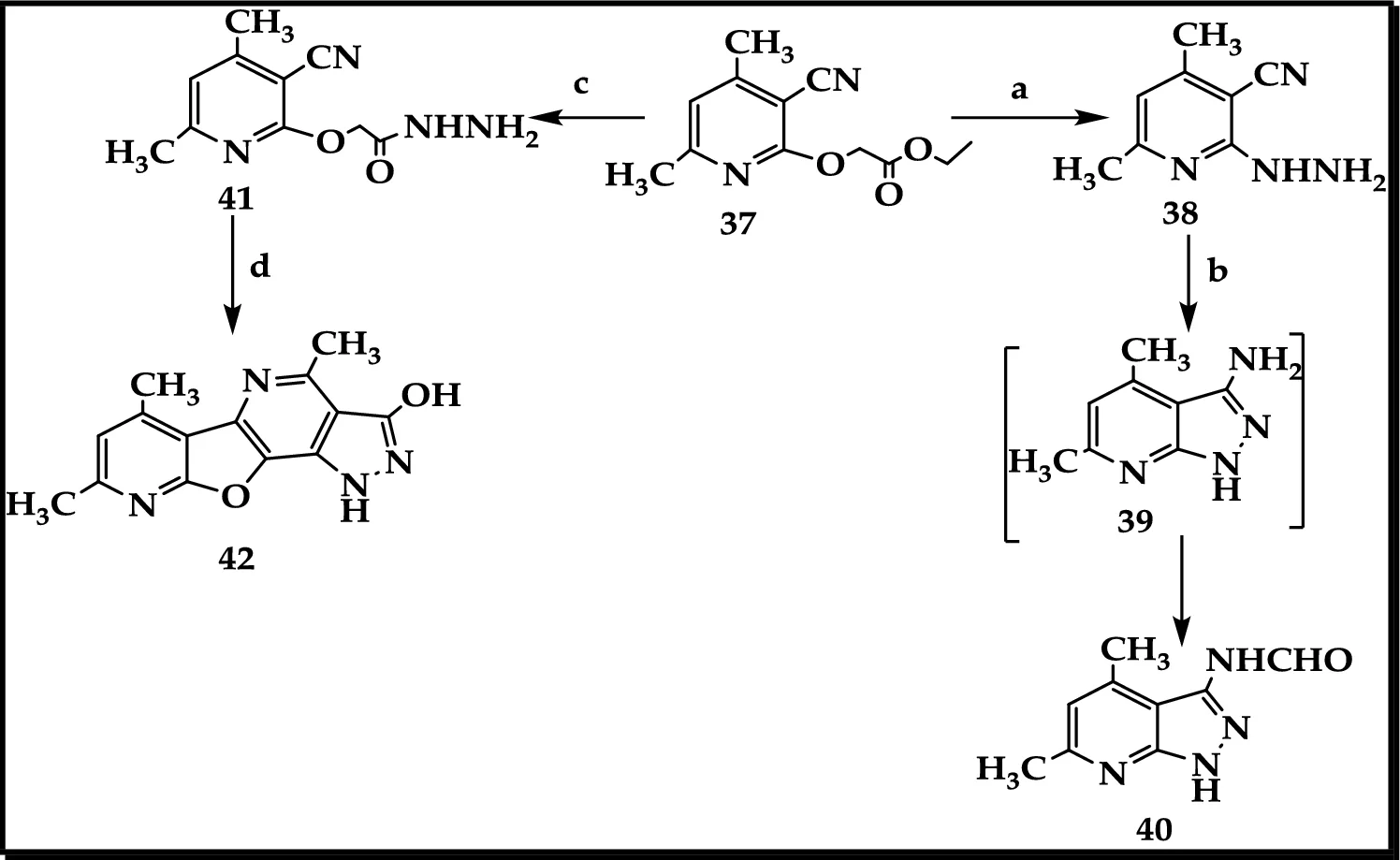
Synthesis of 1*H*-pyrazolo[3,4-*b*]pyridine from hydrazide 41. Reagents and conditions: (a), (c) Hydrazine hydrate abs. EtOH, reflux, 2 h. (b) Formamide, reflux, 4 h; (d) Hydrazine hydrate, abs. EtOH, stirring, rt. 2 h

In 2020, Keshk [48] reported a simple method to prepare pyrazole from pyridine derivative. In detail, treatment of compound **37** with hydrazine hydrate afforded a mixture of **38** (18%) and **41** (57%). Then the reaction of **38** with formamide afforded pyrazolo[3,4-*b*]pyridine **40**, where amino group of hydrazinopyridine attack cyano carbon on heating and afforded pyrazolo [3,4-*b*]pyridine **39** as intermediate. Furthermore, the reaction of compound **41** with ethyl acetoacetate in the presence of potassium carbonate afforded compound **42** (Scheme 15).

**Scheme 16:**
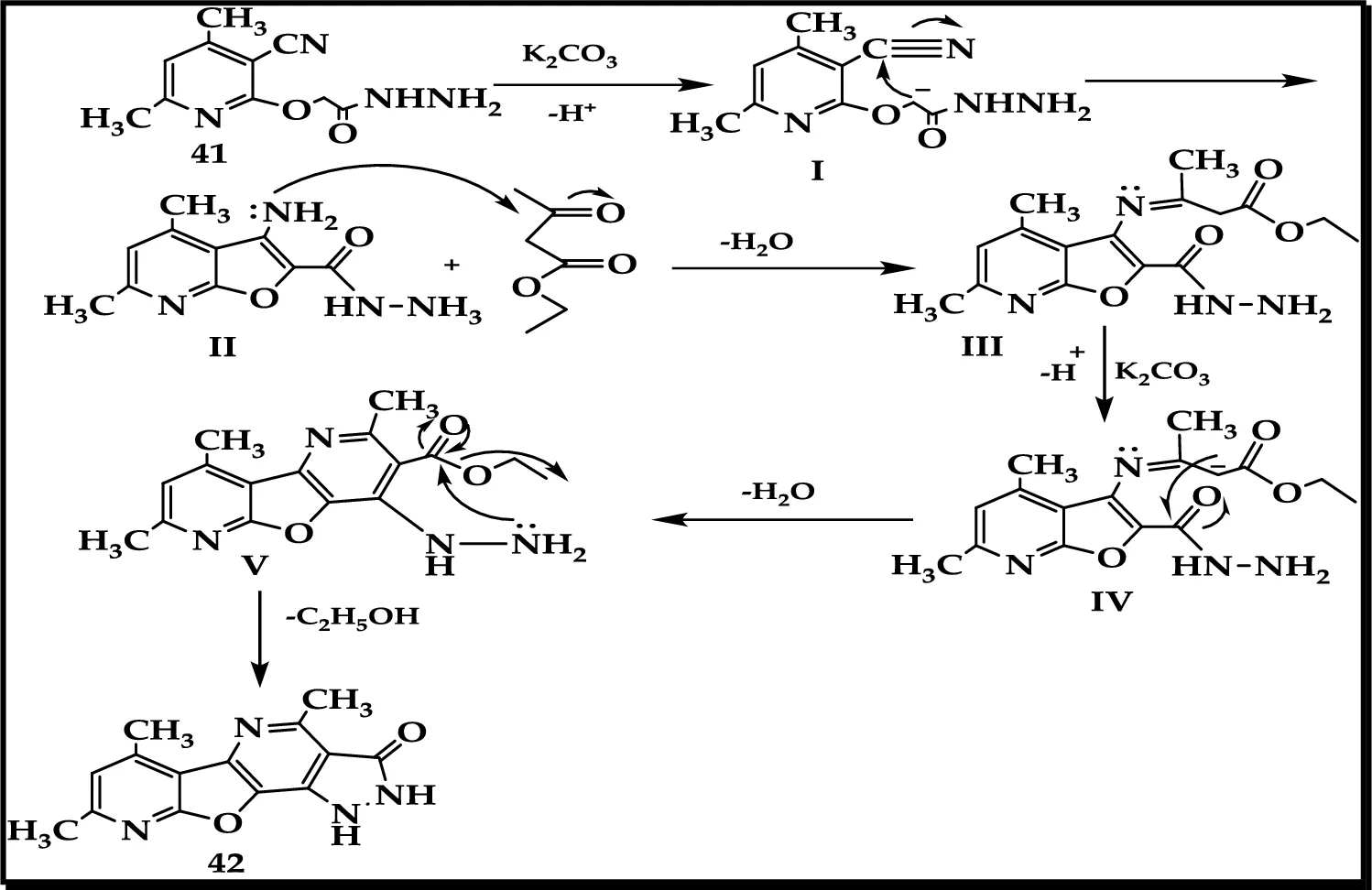
Mechanism for the synthesis of 1*H*-pyrazolo[3,4-*b*]pyridine 42 from hydrazide 41

The conversion of compound **41** to **42** could be explained according to the following mechanism (Scheme 16).

**Scheme 17:**
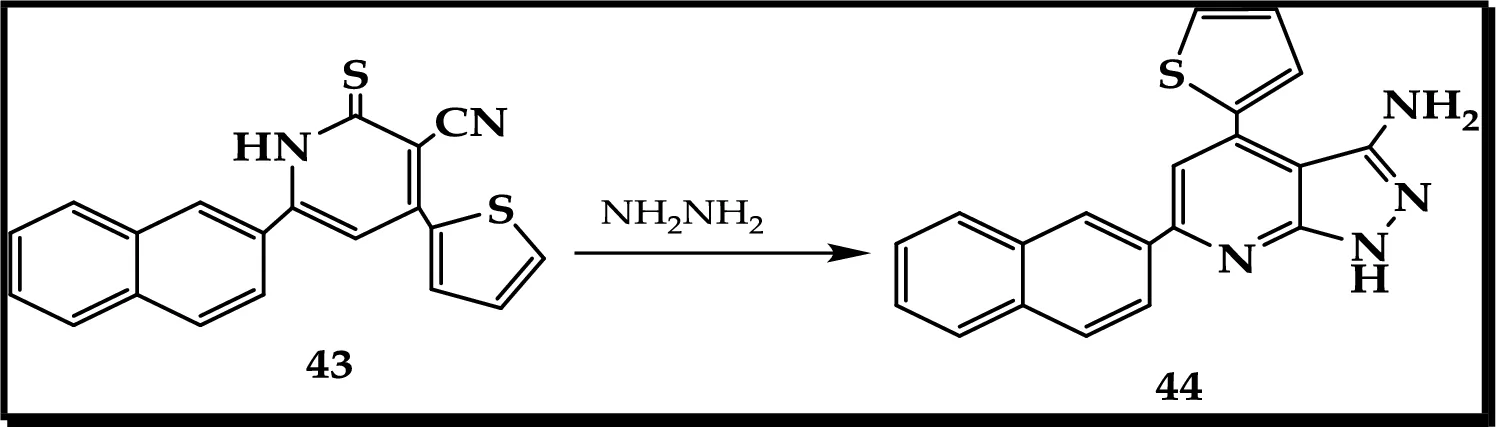
Synthesis of 3-amino-1*H*-pyrazolo[3,4-*b*]pyridine derivative 44

In 2021, Abdel-Rahman [12] and his group synthesized 3-amino-1*H*-pyrazolo[3,4-*b*]pyridine derivative **44** by the reaction of pyridine-2(1*H*) thione derivative **43** with hydrazine hydrate in ethanol under reflux conditions (Scheme 17).

**Scheme 18:**
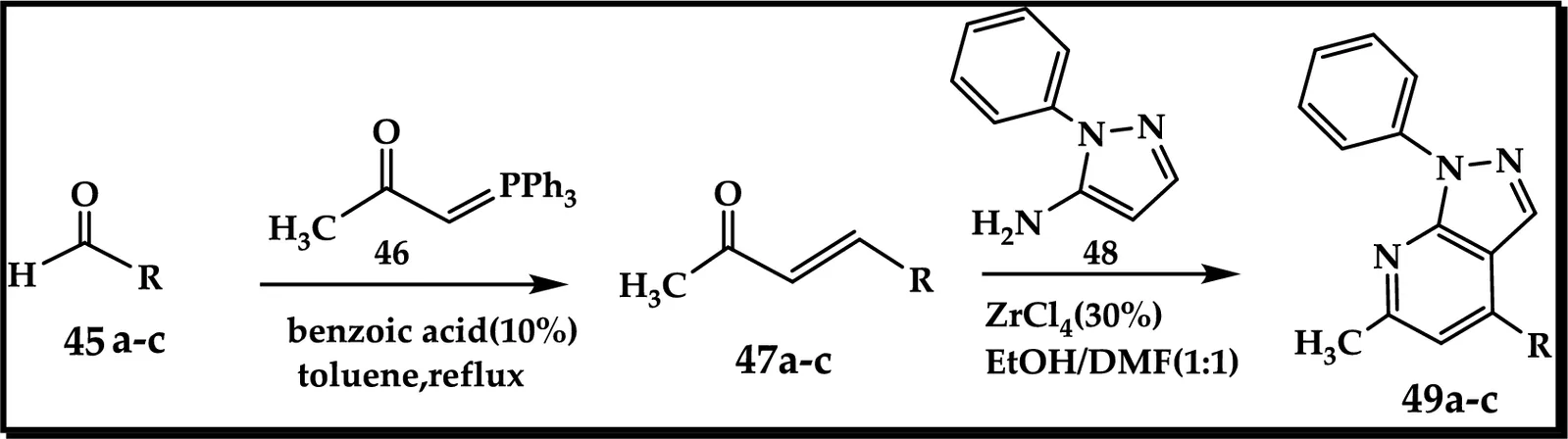
Wittig reaction for synthesis of pyrazolo[3,4-*b*]pyridine derivatives

Vidali et al*.* [49] developed new pyrazolo[3,4-*b*]pyridine derivatives **49a–c** by Wittig reaction of an aldehyde **45a-c** with a triphenyl phosphonium(1-(triphenyl phosphaneylidene)propan-2-one) **46** to give 4-arylbut-3-en-2-ones **47a–c** followed by the reaction with 5-amino-1-phenyl pyrazole **48** in the presence of ZrCl_4_ as catalyst (Scheme 18).

**Scheme 19:**
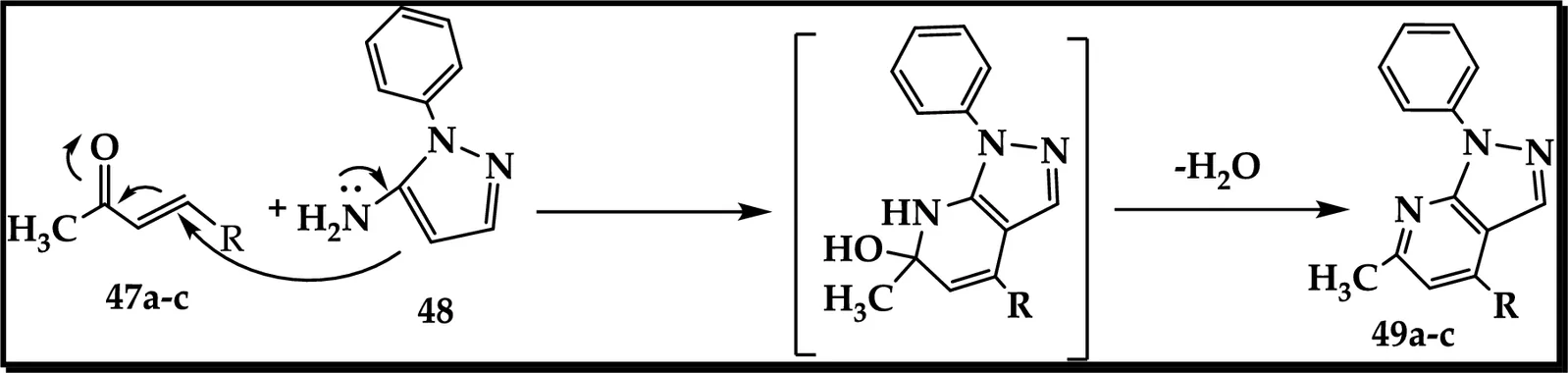
The suggested mechanism for the formation of 1-*H*-pyrazolo[3,4-*b*]pyridine derivatives 49

The suggested mechanism that explains the formation of pyrazolo[3,4-*b*] pyridine derivatives **49** is illustrated in Scheme 19. The compounds **49a-c** are assumed to be produced by Michael addition of pyrazole **48** to the β- carbon of chalcone followed by nucleophilic attack of amino group on carbonyl carbon. The final product is produced through elimination of water molecule.

**Scheme 20:**
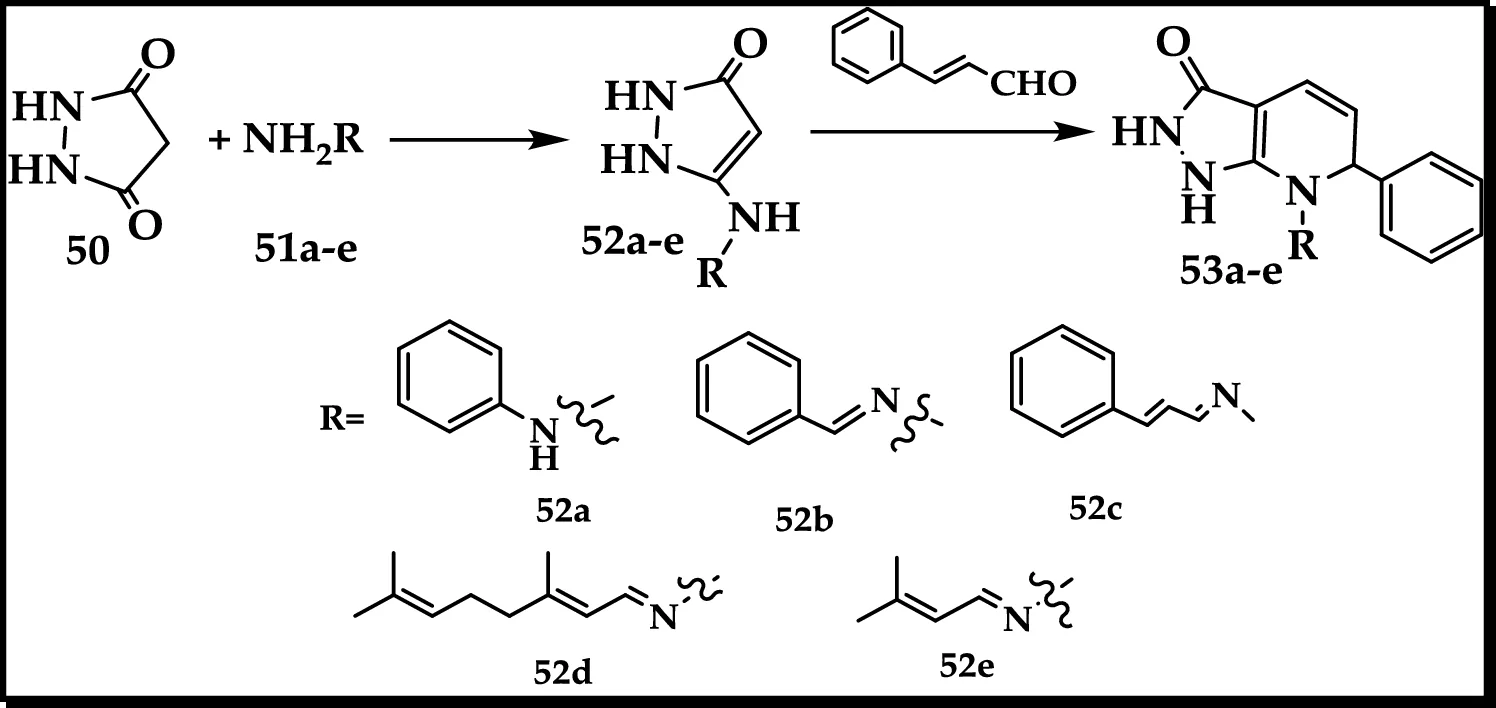
Reaction of pyrazolidine-3,5-dione and hydrazine derivatives to produce pyrazolopyridines 53a-e

Furthermore, pyrazolidine-3,5-dione and hydrazine derivatives were heated at 100 °C to give 5-(2-phenylhydrazinyl)-1-*H*-pyrazol-3(2*H*)-one **52a** which was treated with cinnamaldehyde in EtOH to give pyrazolo [3,4-*b*]pyridin-3(2*H*)-one **53a-e** [50] (Scheme 20).

**Scheme 21:**
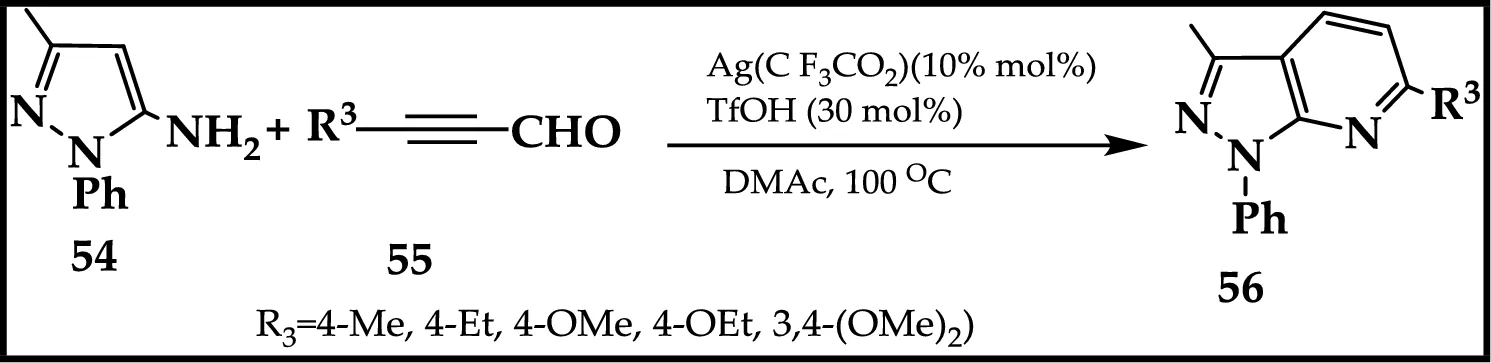
Reaction of 3-methyl-1-phenyl-1*H*-pyrazol-5-amine 54 and 3(substituted phenyl propionaldehydes) 55

Miao et al*.* [51] proposed that the reaction of 3-methyl-1-phenyl-1*H*-pyrazol-5-amine **54** and 3(substituted phenyl propionaldehydes) **55** afforded pyrazolo[3,4-*b*]pyridines **56** with (66–81%) yields through optimization of the catalyst, solvent, and temperature (Scheme 21).

**Scheme 22:**
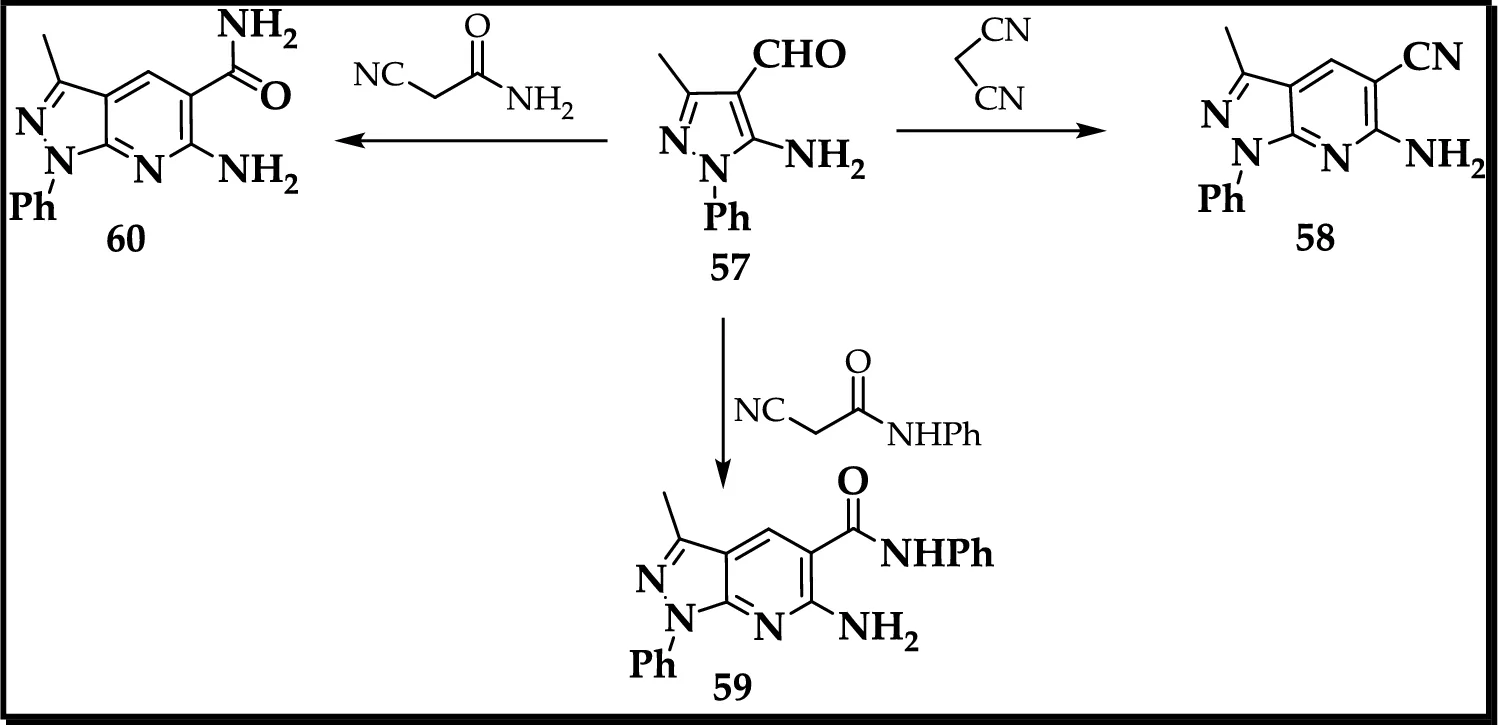
Synthesis of 1*H*-pyrazolo[3,4-*b*]pyridines using 5-aminopyrazole and active methylene compounds

Abeed et al. [52] reported the reaction of compound **57** with active methylene compounds, namely, malononitrile, phenylcyanoacetamide, and /or cyanoacetamide in ethanol under reflux to afford 5-substituted-6-amino-3-methyl-1-phenyl-1*H*-pyrazolo[3,4-*b*]pyridine **58–60** as illustrated in Scheme 22.

**Scheme 23:**
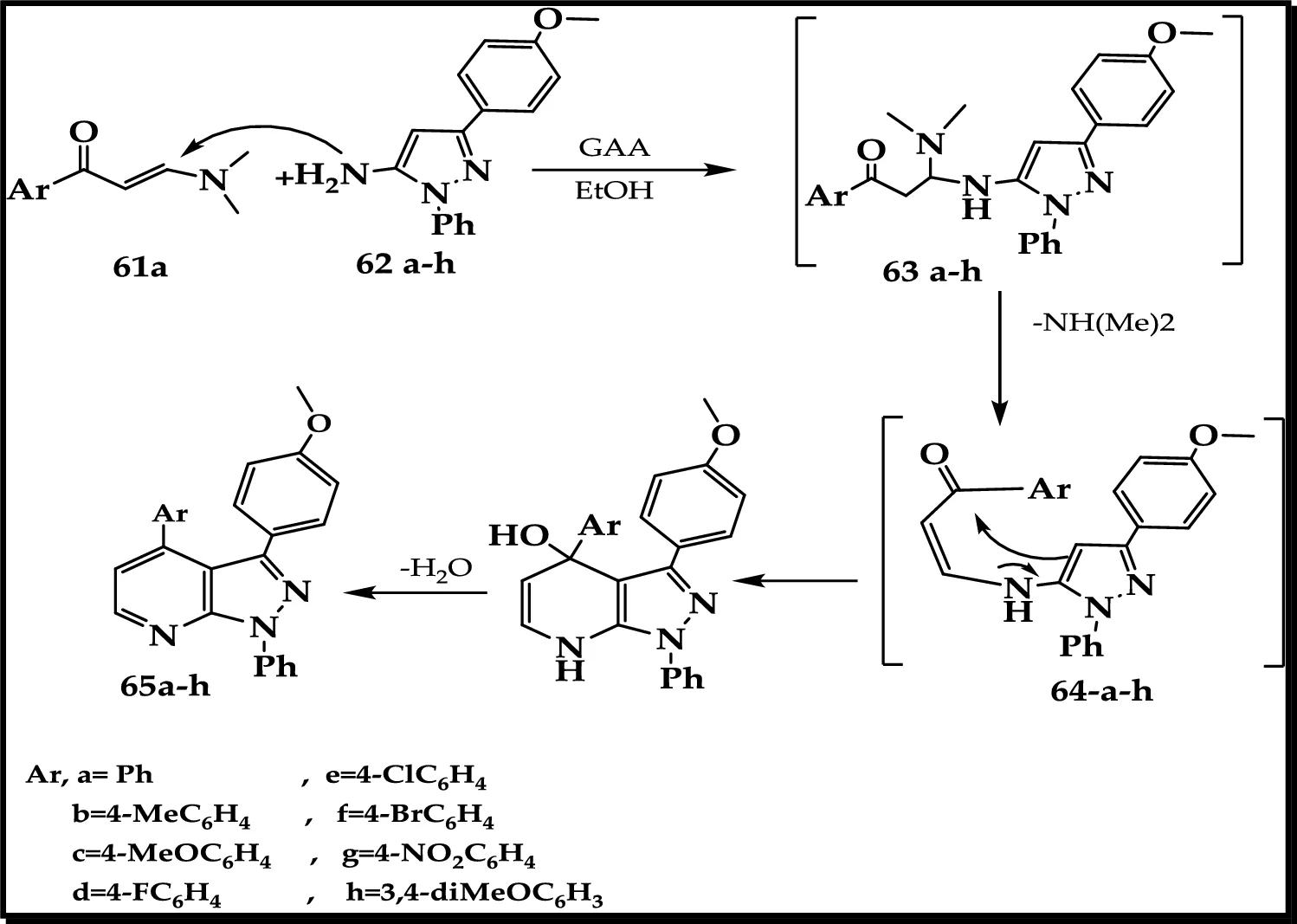
Synthesis of 1*H*-pyrazolo[3,4-*b*]pyridines from enaminone 61 and 5- aminopyrazoles 62a-h

Moreover, 3-(4-Methoxyphenyl)-pyrazolo[3,4-*b*]pyridines **65a**–**h** were prepared via the reaction of amino pyrazole derivative **62a**–**h** with enaminones **61**. The reaction may be preceded through Michael addition of exocyclic NH_2_ of amino pyrazole **62a–h** into the double bond of enaminones **61** to form intermediate **63a**–**h**. The later lost a dimethylamine molecule to give intermediate **64a**–**h**, followed by an intramolecular cyclocondensation reaction to give the targeted compounds via elimination of water molecule **65a–h** [53] (Scheme 23).

**Scheme 24:**
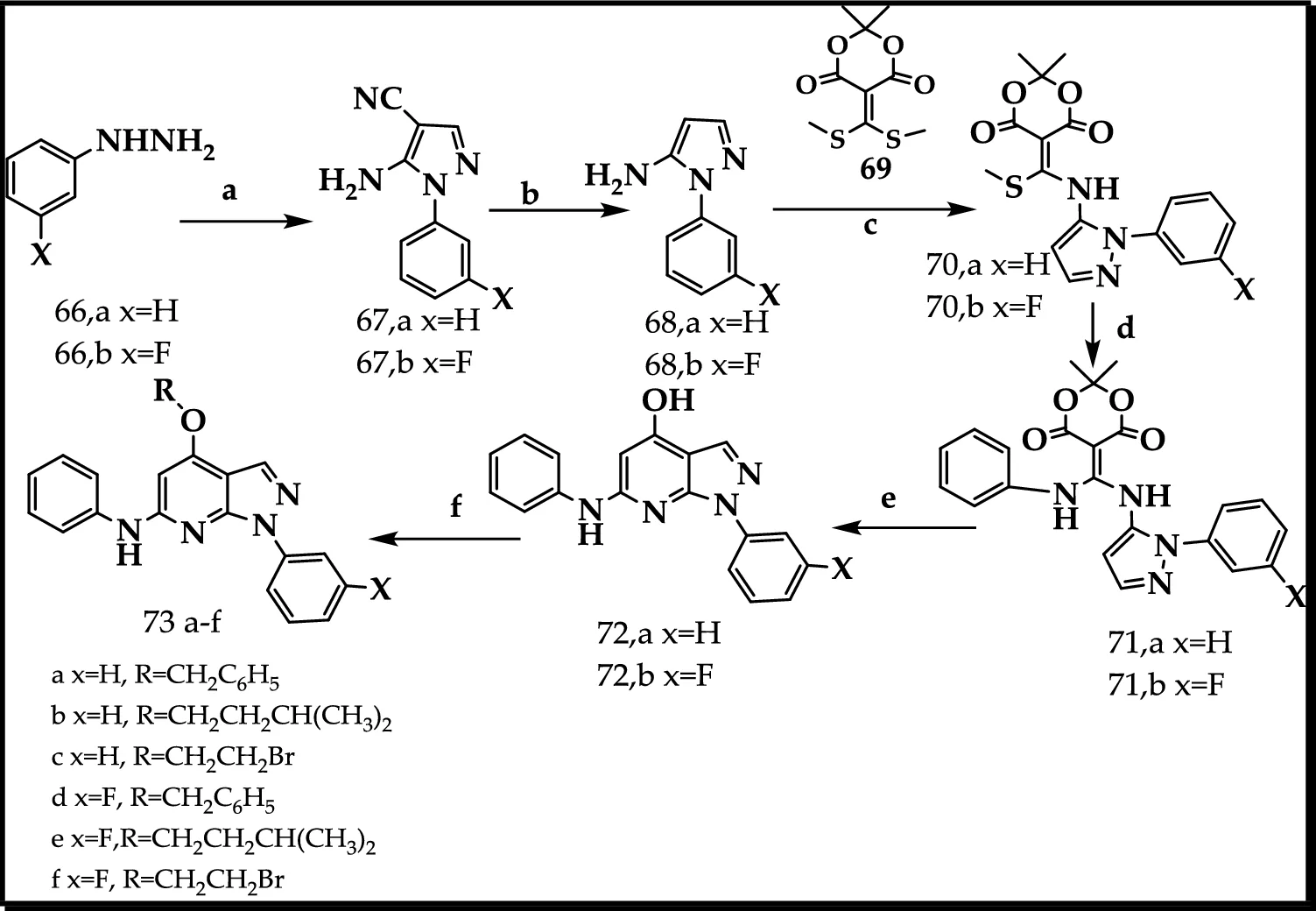
Synthesis of 1*H*-pyrazolo[3,4-*b*]pyridines 73a-f via the reaction of pyrazoles 68a, b with the Meldrum’s acid. Reagents and conditions. (a) (ethoxymethylene)malononitrile, EtOH abs., reflux, Ar. (b) H_3_PO_4_, reflux. **c** EtOH, reflux. (d) aniline, EtOH, reflux (e), Ph_2_O, reflux, Ar (f) K_2_CO_3_, DMF

Also, 1*H*-pyrazolo[3,4-*b*]pyridines **73a-f** were prepared via the reaction of pyrazoles **68a,b** with the Meldrum’s acid **69** (dimethythiomethylene-2,2-dimethyl-1,3-dioxane-4,6-dione) to give the intermediate dioxanedions **70a**,**b**. The methyl thio group of compounds **70a,b** was displaced upon reaction with aniline afforded dioxanediones **71a,b**. The latter was cyclized under thermal condition yielded pyrazolo[3,4-*b*]pyridinols **72a**,**b** which when treated with K_2_CO_3_ provided the corresponding ethers **73a**-**f** [54] (Scheme 24).

**Scheme 25:**
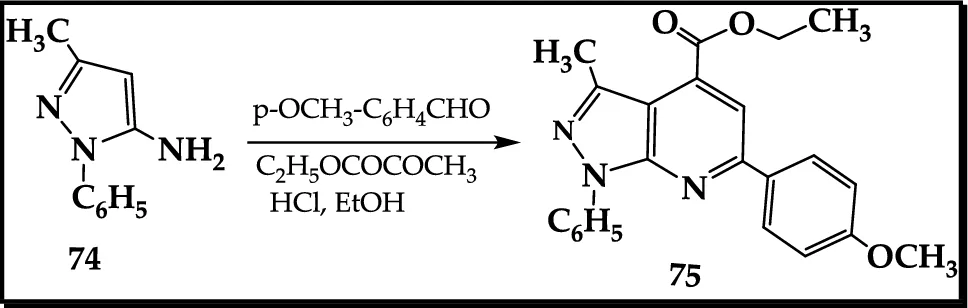
Synthesis of ethyl 3-methyl-1-phenyl-6-aryl-1*H*-pyrazolo[3,4-*b*]pyridine-4-carboxylate 75

Ramzan et al. [55] synthesized ethyl 3-methyl-1-phenyl-6-aryl-1*H*-pyrazolo[3,4*-b]*pyridine-4-carboxylate **75** via the reaction of 5-amino-3-methy-1-phenyl-1*H*-pyrazole **74** with *p*-methoxybenzaldehyde as illustrated in Scheme 25.

#### Three-component reactions

**Scheme 26:**
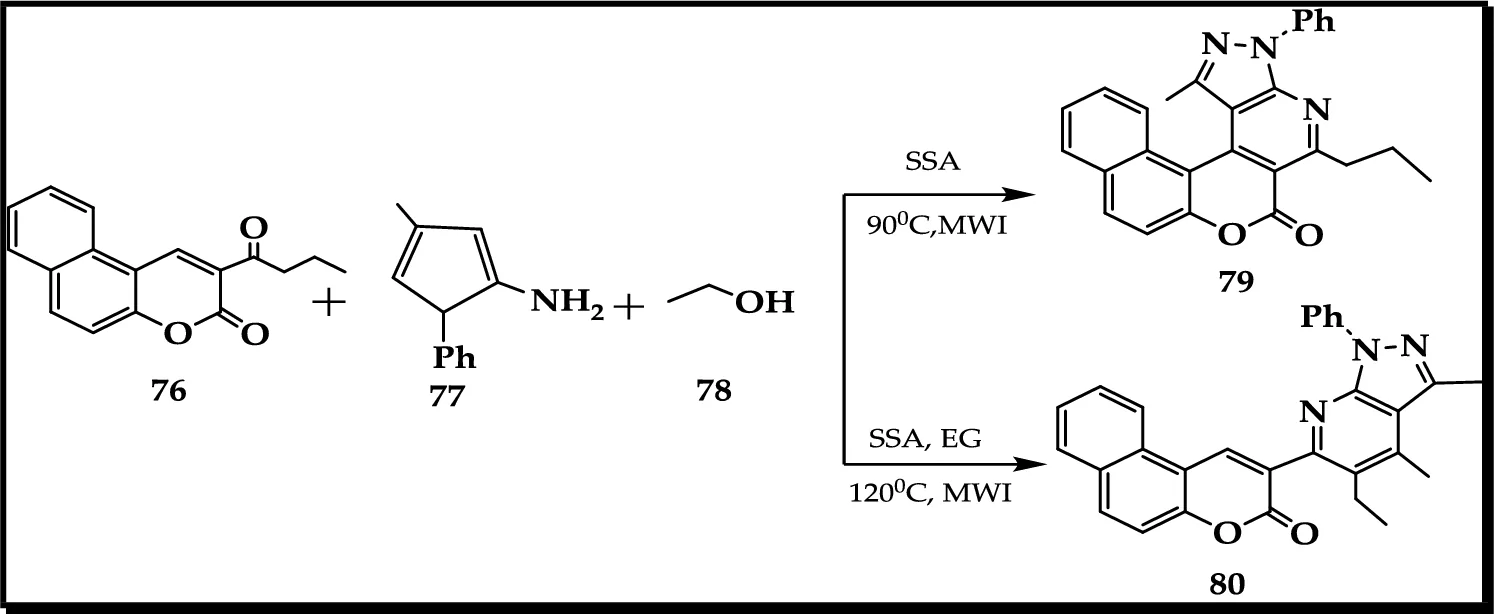
Microwave irradiation synthesis of pyrazolo[3,4-*b*]pyridine derivatives 79 and 80

Under microwave irradiation pyrazolo[3,4-*b*]pyridine derivatives **79** and **80** were obtained through one-pot reaction of 2-butyryl-3*H*-benzo[*f*]chromen-3-one **76**, 3-methyl-1-phenyl-1*H*-pyrazol-5-amine **77** and alkyl alcohol **78** catalyzed by silica sulfuric acid (SSA) in EtOH at 90 °C for 20 min [56] (Scheme 26).

**Scheme 27:**
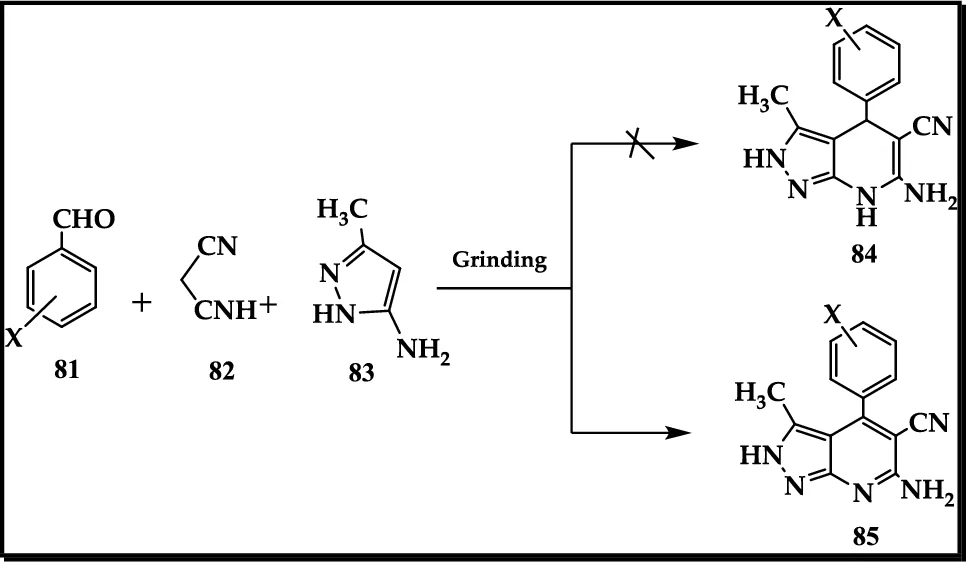
Synthesis of pyrazolo[3,4-*b*]pyridines by grinding method

On the other hand, the synthesis of pyrazolo[3,4-*b*]pyridines were accomplished by grinding of benzaldehydes **81**, malononitrile **82**, and 3-amino-5-methylpyrazole **84**. This solvent-free grinding method is more efficient; low-cost with 95% yield and in very short time (5 min) compared to classical Lewis acids such as ZnCl_2_, K10, and nano-Fe_3_O_4_ or ionic liquids [57] (Scheme 27).

**Scheme 28:**
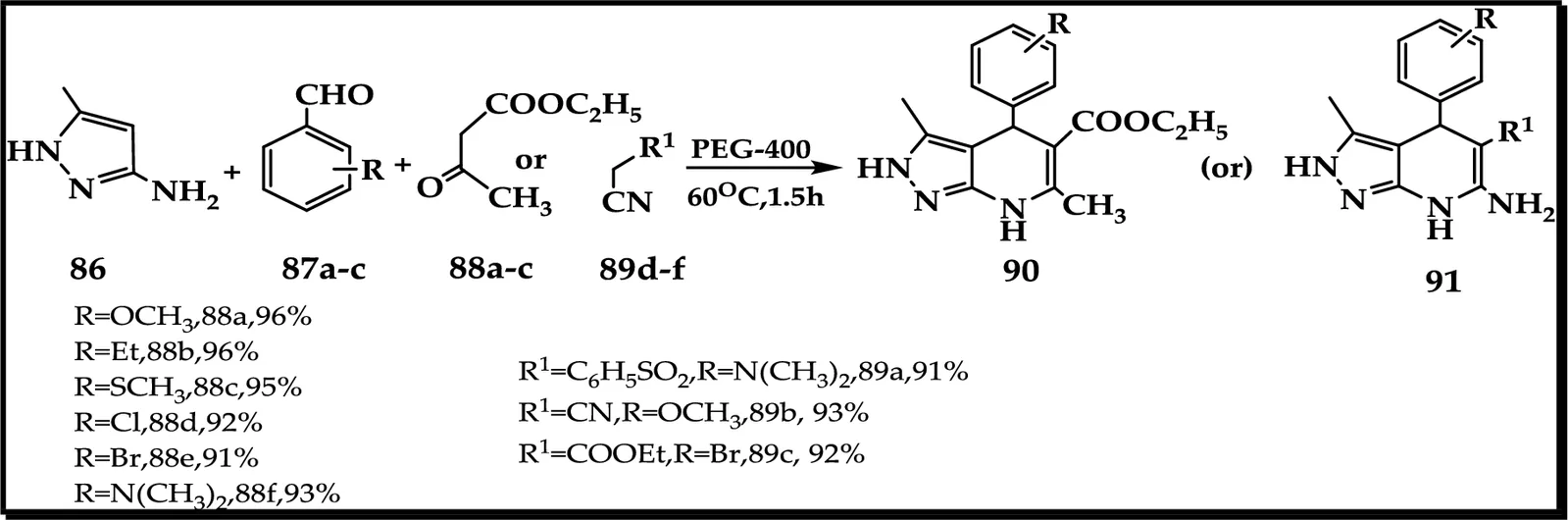
One pot three-component synthesis of pyrazolo[3,4-*b*]pyridine under catalyst-free condition

Kerru et al. [58] designed a one-pot three-component synthesis of 4,7-dihydro- 2*H*-pyrazolo[3,4-*b*]pyridine-5-carboxylate **90** by reaction of an equimolar amount of 1*H*-pyrazol-3-amino-5-methyl **86**, benzaldehyde derivatives **87**, and ethyl acetoacetate **88** in (PEG-400) at 60 °C under catalyst-free conditions (Scheme 28).

**Scheme 29:**
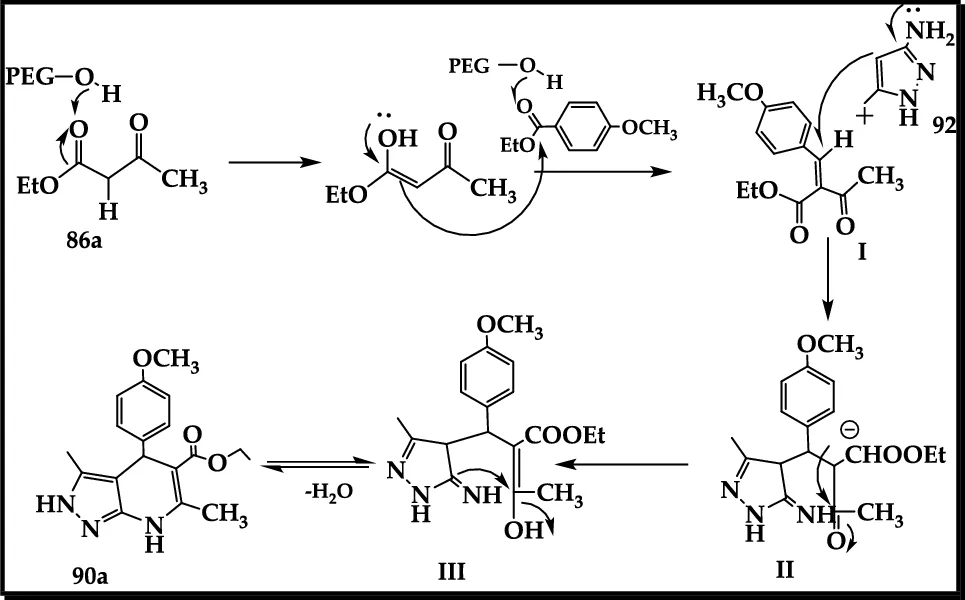
Mechanism for the synthesis of pyrazolo[3,4-*b*]pyridine 90

A possible mechanism for the formation of 3,6-dimethyl-4,7-dihydro-2*H*-pyrazolo[3,4-*b*]pyridine **90** (Scheme 29).

**Scheme 30:**
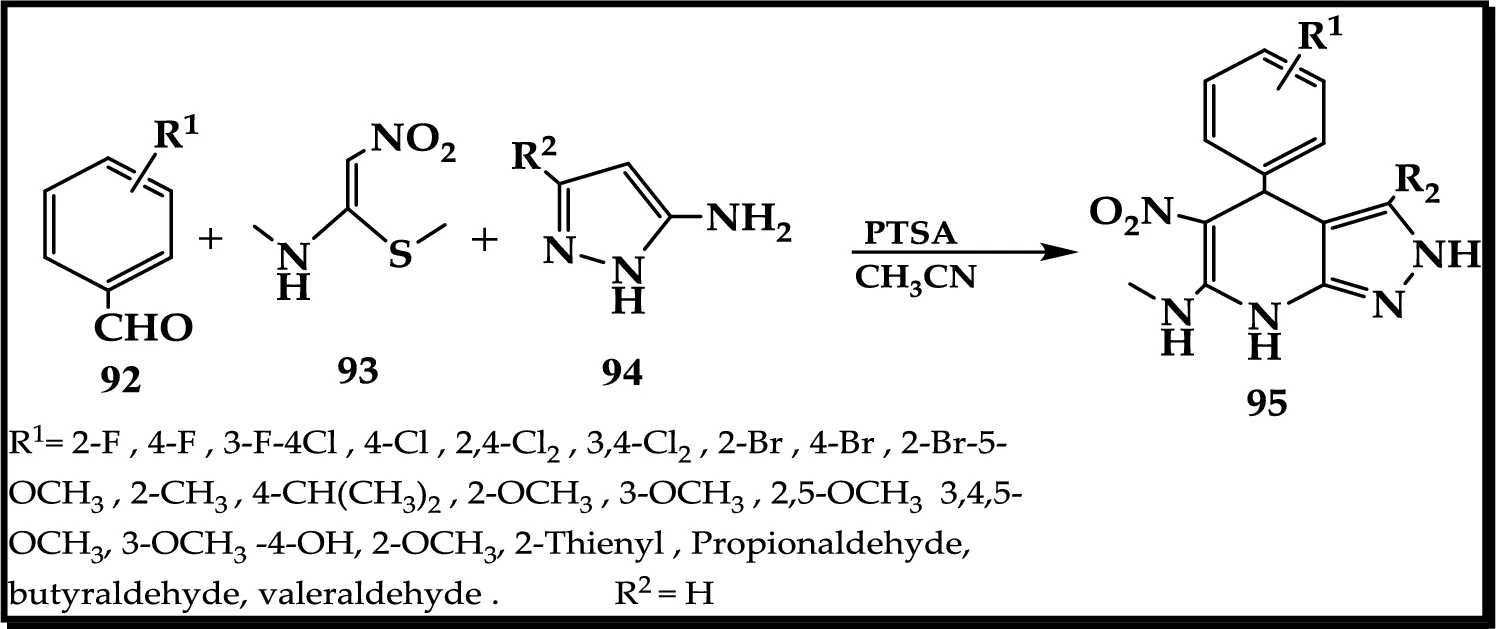
Synthesis of pyrazolo[3,4-*b*] pyridines three-component

Ji et al. [59] developed a three-component reaction of benzaldehyde derivatives **92**, *N-*methyl-1-(methylthio)-2-nitroethen-1-amine **93** and 3-amino pyrazole **94** in the presence of *p*-toluenesulfonic acid (PTSA) to furnish pyrazolo[3,4-*b*] pyridines **95** in 45% yield (Scheme 30).

**Scheme 31:**
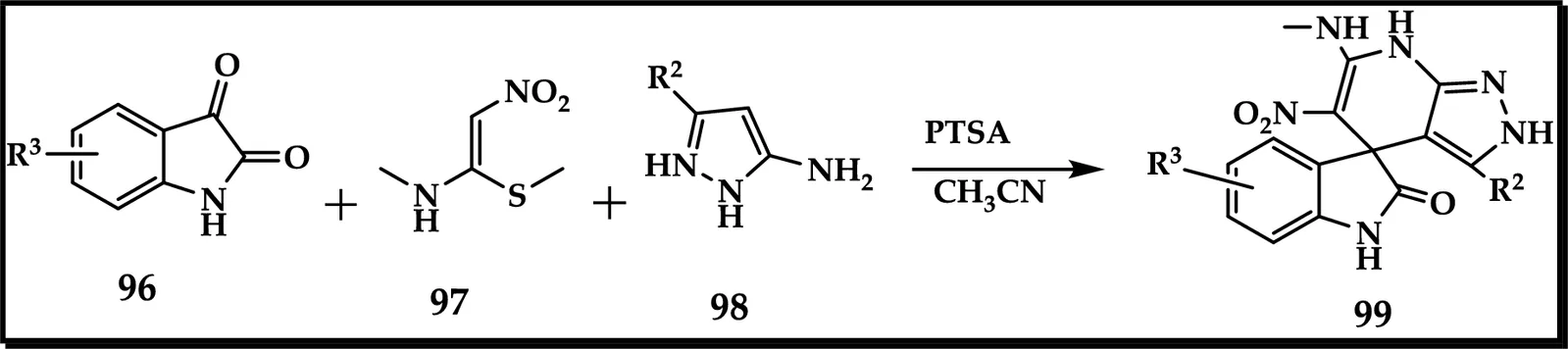
Synthesis of pyrazolo[3,4-*b*]pyridine derivative 98

In addition, the reaction of isatin **96** that bears electron-donating (Me and OMe) or electron-withdrawing groups (F, Cl, Br, I, and NO_2_) on the phenyl ring, *N*-methyl-1-(methylthio)-2-nitroethen-1-amine **93**, and 3-aminopyrazole **98** afforded the corresponding pyrazolo[3,4-*b*]pyridine derivatives **98** in 81–90% yield [59] (Scheme 31).

**Scheme 32:**
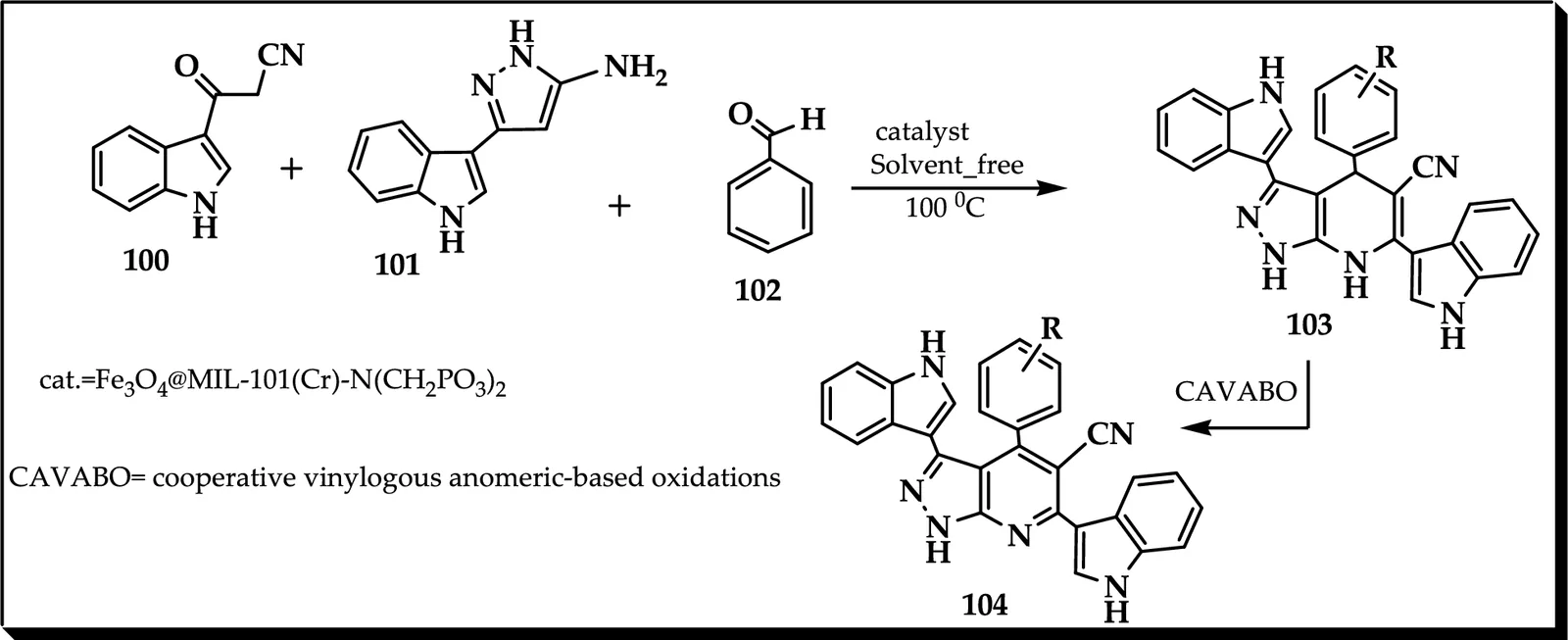
Synthesis of pyrazolo[3,4-*b*]pyridine 103 using nanocatalyst

Sepehrmansourie et al*.* [60] designed the synthesis of substituted pyrazolo[3,4-*b*]pyridines derivative **104** through one-pot reaction of 3-(cyanoacetyl) indole **100**, 5-(1*H*-indol-3-yl)-2*H*-pyrazol-3-ylamine **101**, aldehyde derivative **102,** and nano-magnetic metal–organic frameworks Fe_3_O_4_@MIL-101(Cr)-N(CH_2_ PO_3_)_2_ as catalyst under stirring at 100 °C (Scheme 32).

**Scheme 33:**
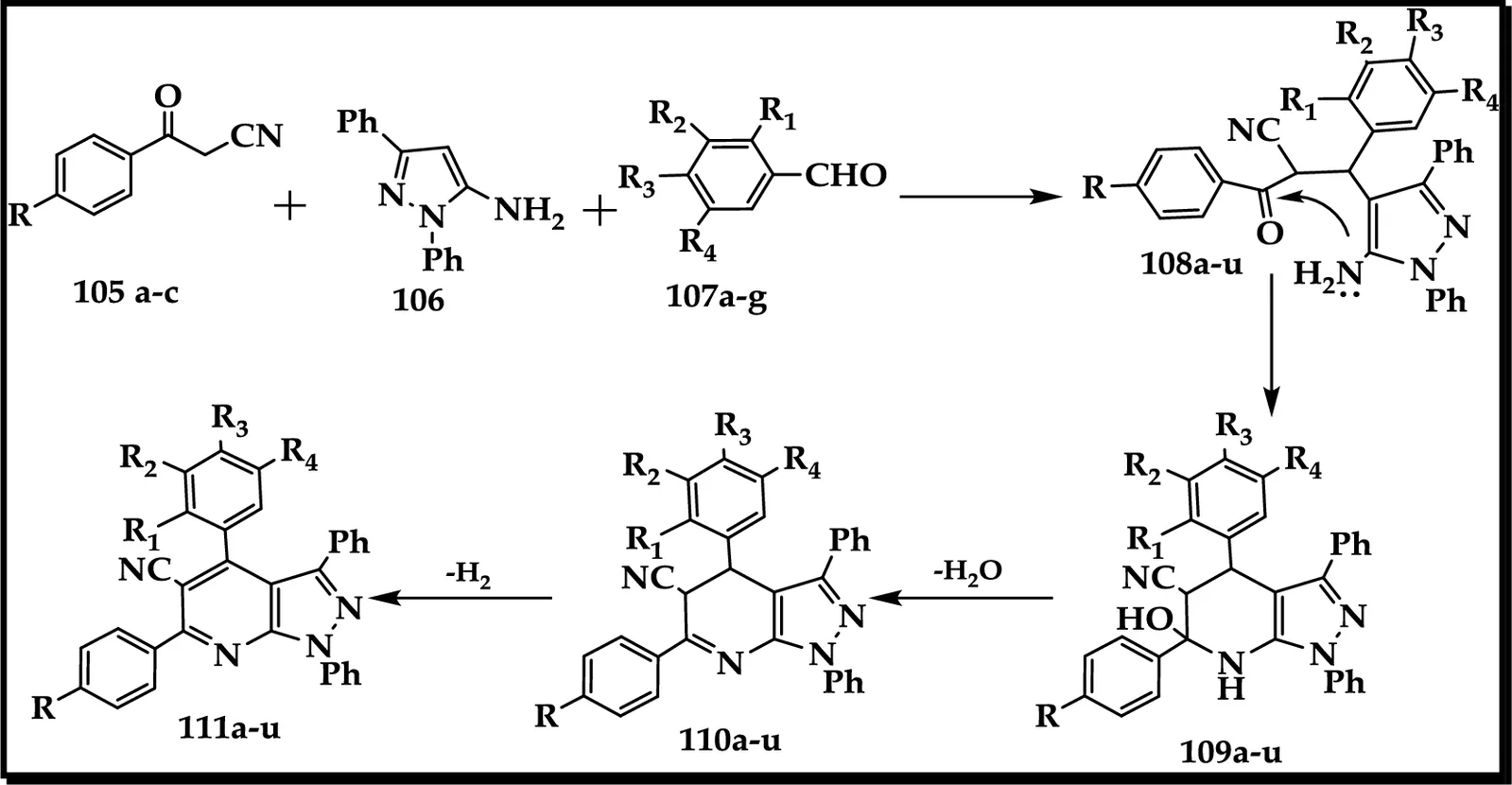
One-pot three component synthesis of pyrazolo[3,4-*b*]pyridine

Furthermore, preparation of target pyrazolo[3,4-*b*]pyridine derivatives **110a-u** were carried out through one-pot three-component reaction of the corresponding aldehyde **106a-g** with an equimolar amount of 3-oxo-3-arylpropanenitriles **104a-c** and 1,3-diphenyl pyrazol-5-amine **105** in refluxing absolute ethanol [1] (Scheme 33).

**Scheme 34:**
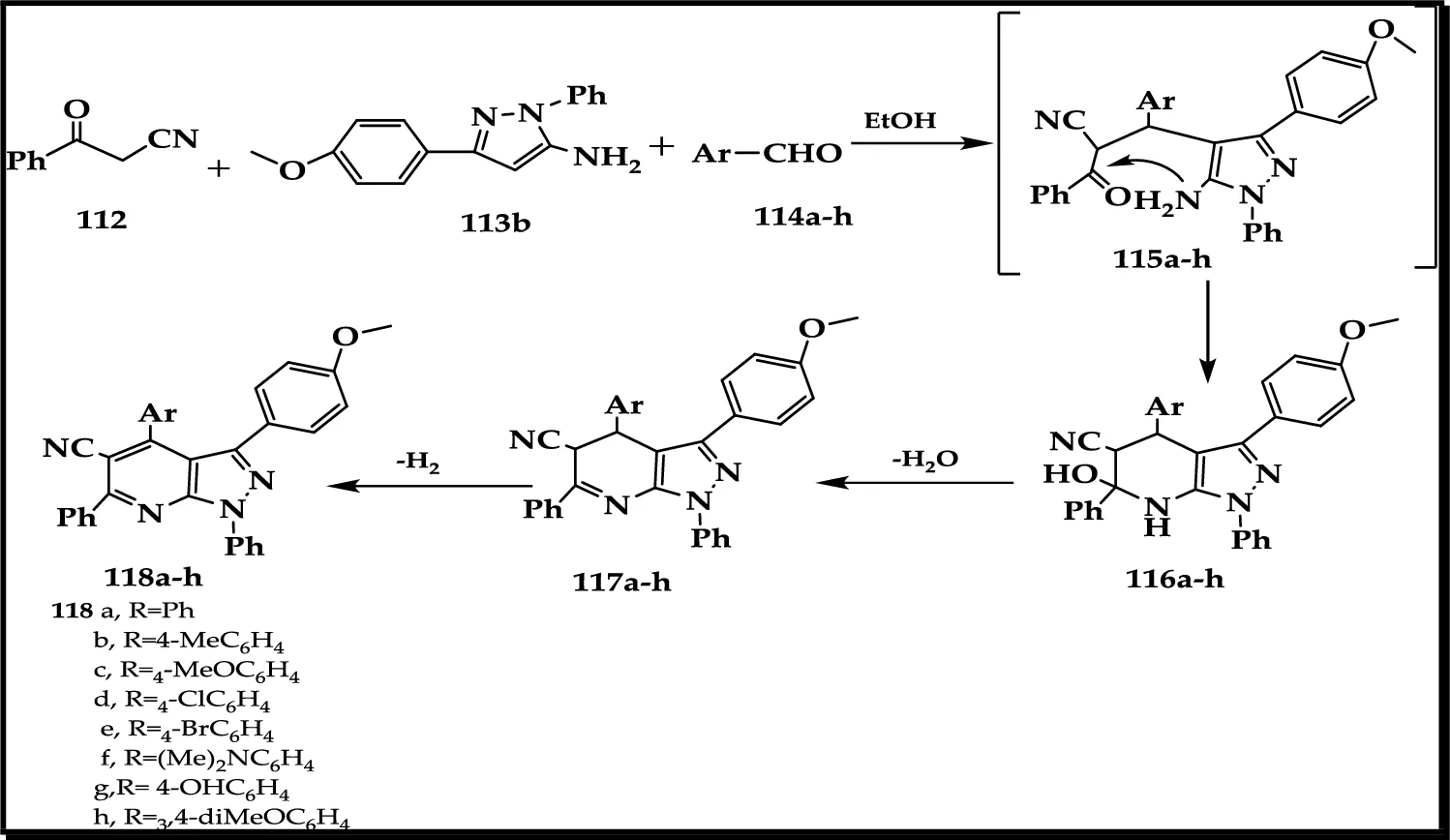
Synthesis of pyrazolo[3,4-*b*]pyridine-5-carbonitriles 118a–h

Moreover, pyrazolo[3,4-*b*]pyridine-5-carbonitriles **117a-h** were prepared via three component reaction of 3-oxo-3-phenylpropanenitrile **111**, pyrazol-5-amine **112**, and aromatic aldehydes **113a-h** in ethanol. The reaction may be proceed via the condensation reaction between 3-oxo-3-phenylpropanenitrile **111** and aldehydes **113a-h**, then the addition of NH2 of pyrazole to give intermediate **114a-h**. Followed by intramolecular cyclocondensation of yielded the target **114a-h** compounds via elimination of water molecule followed by oxidation [53] (Scheme 34).

**Scheme 35:**
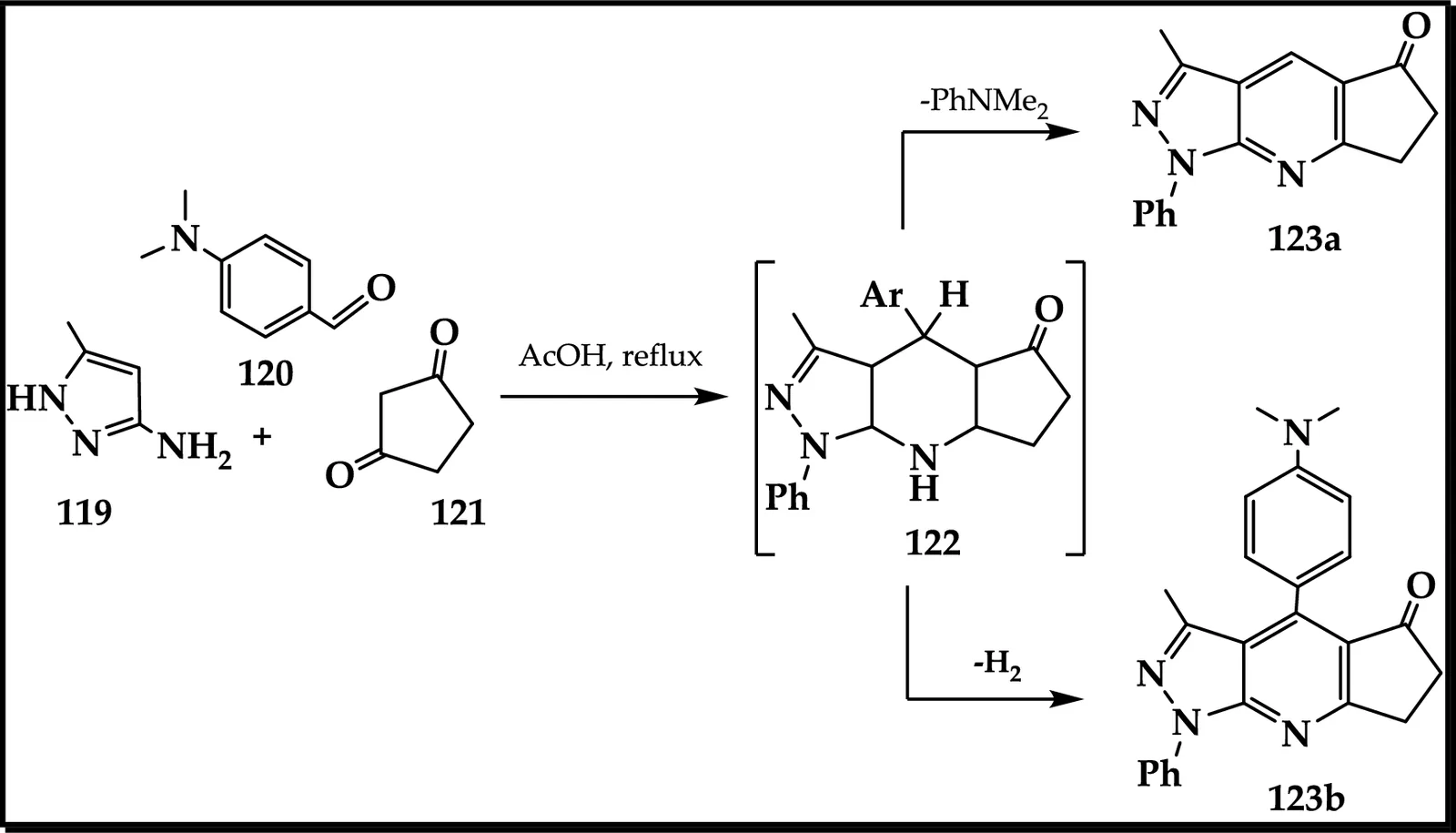
Reaction of amino pyrazole with aldehyde and diketone

Polo-Cuadrado et al. [61] reported one-pot reaction of 5-aminopyrazole **119**, 4-(dimethylamino)benzaldehyde **120**, and cyclopentane-1,3-dione **121** in glacial acetic acid at 120 °C to provide pyrazolo[3,4-*b*]pyridine derivatives **123a** and **123b**, respectively (Scheme 35).

**Scheme 36:**
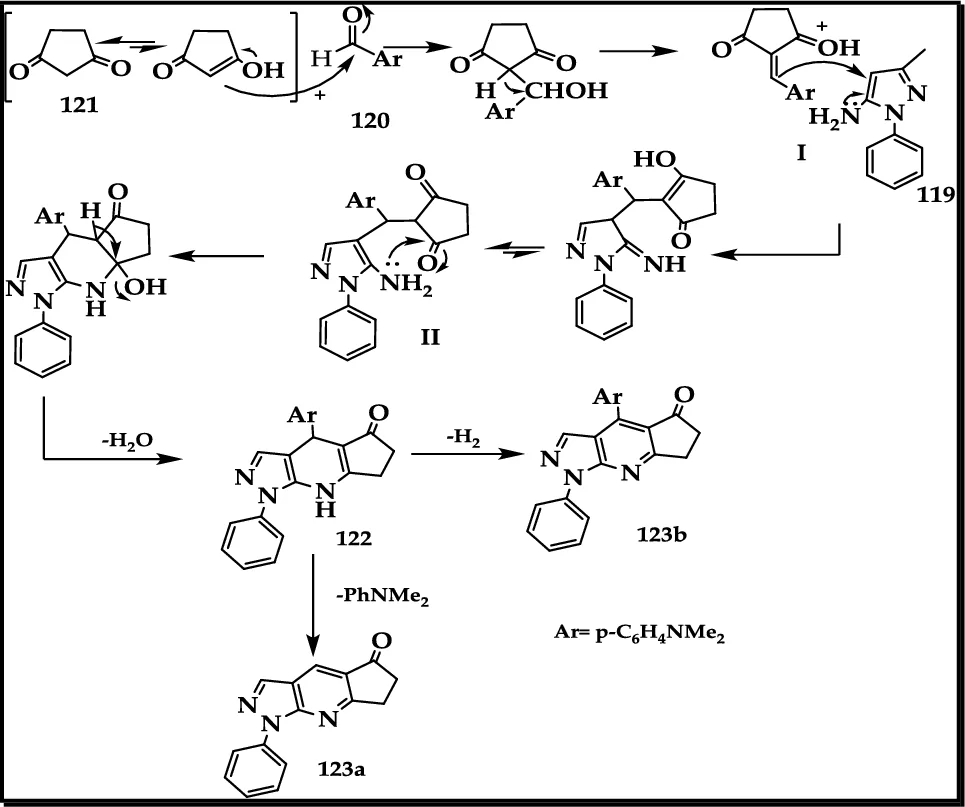
Mechanism for the formation of pyrazolo[3,4-*b*]pyridine derivatives 122a, b

A possible mechanism for the formation of pyrazolo[3,4-*b*]pyridine derivatives **123a** and **123b** is illustrated in Scheme 34. The formation proceeds via initial condensation of aldehyde **120** with cyclopentane-1,3-dione **121** to give an intermediate **I**. which then goes through Michael addition with **119** to produce an open-chain intermediate **II**. This is then cyclized, dehydrated, and dehydrogenated to provide the aromatized intermediate **122**, during which N,N-dimethylaniline splits off and a polycondensed pyrazolo[3,4-*b*]quinoline **123a** and in a separate process, dehydrogenated and elimination of N,N-dimethylaniline to afford the aromatized product **123b **(Scheme 36).

### Four-component reactions

**Scheme 37:**
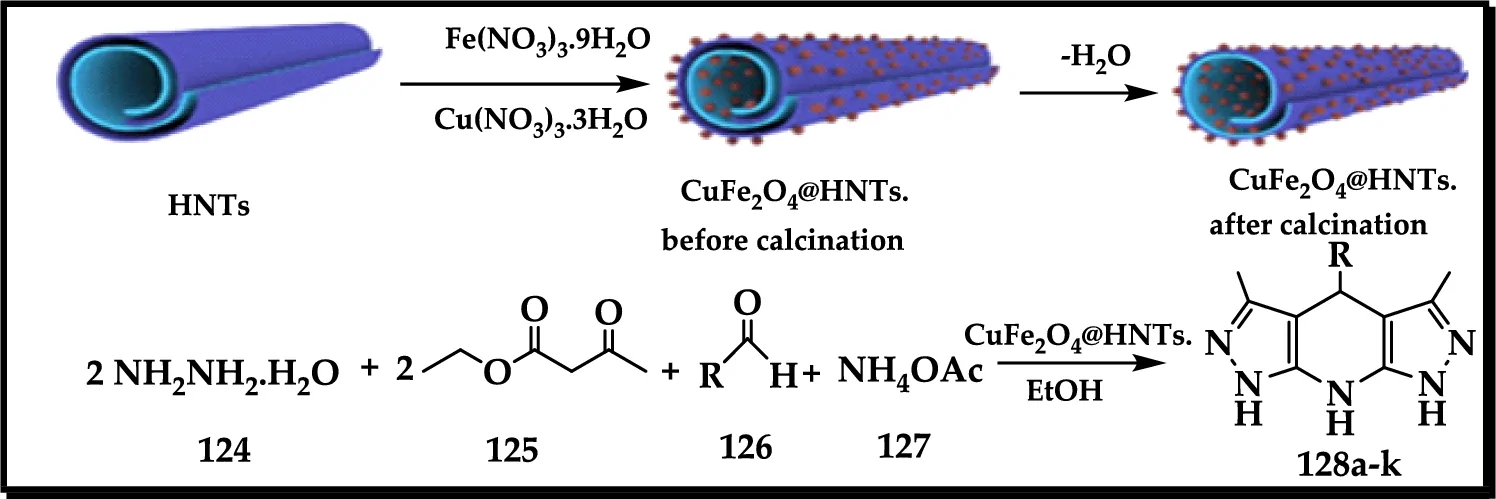
Four component synthesis of pyrazolo[3,4-*b*] pyridine using CuFe2O4@HNts as catalyst

In addition, Maleki et al. [62] synthesized pyrazolo[3,4-*b*]pyridine derivatives **128a-k** via the four-component reaction of hydrazine hydrate **124**, ethyl acetoacetate **125**, benzaldehyde **126**, and ammonium acetate **127** in the presence CuFe_2_O_4_@HNts as catalyst. It was found that CuFe_2_O_4_@HNTs nanocomposite shows an excellent catalyst activity as compared with the single component of CuFe_2_O_4_ or HNTs (Halloysite nanotubes) (Scheme 37).

**Scheme 38:**
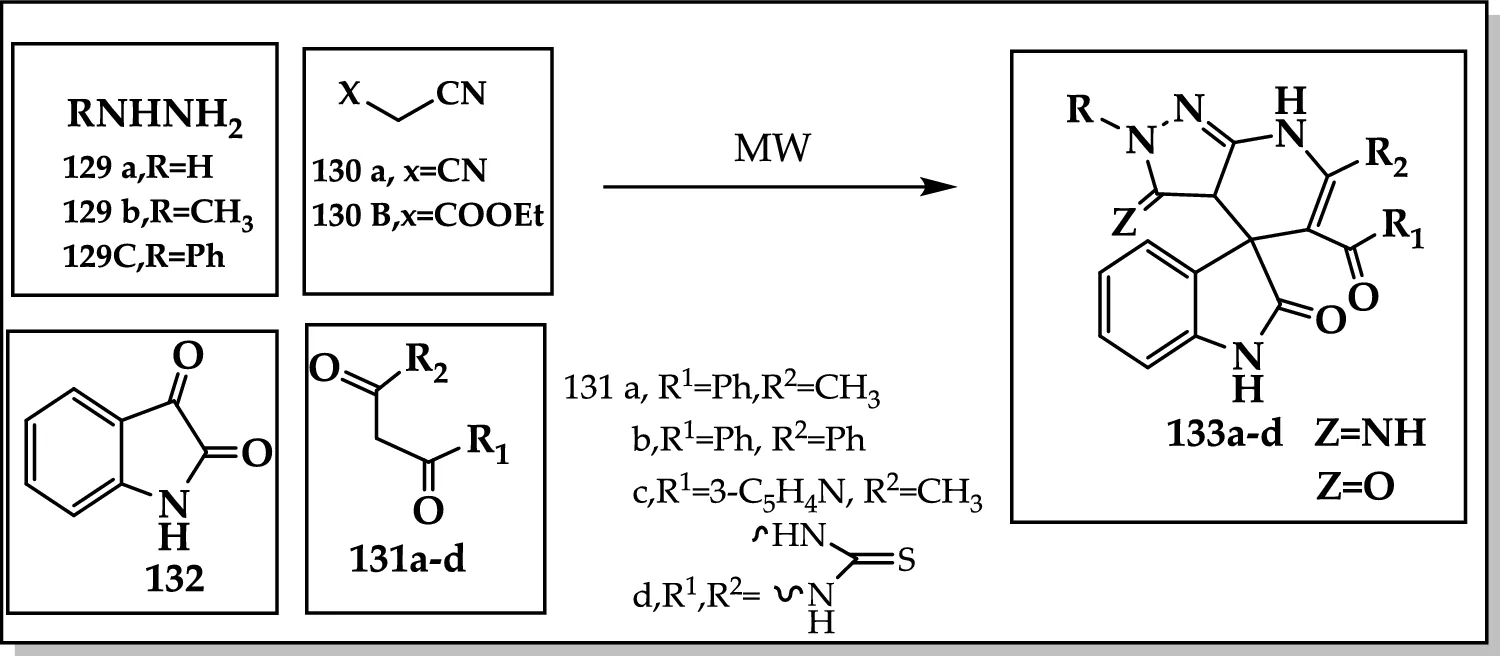
Synthesis of spiro[indoline-3,4′-pyrazolo[3,4-*b*] pyridine under microwave irradiation

Under microwave irradiation, Naglaa et al*.* [63] prepared various spiro[indoline-3,4′-pyrazolo[3,4-*b*] pyridine derivative **133** through multicomponent reaction (MCR) of hydrazine derivatives **129**, active methylene compounds **130,** dicarbonyl compounds **131**, and isatin **132** in the presence of different protic solvents (Scheme 38).

**Scheme 39:**
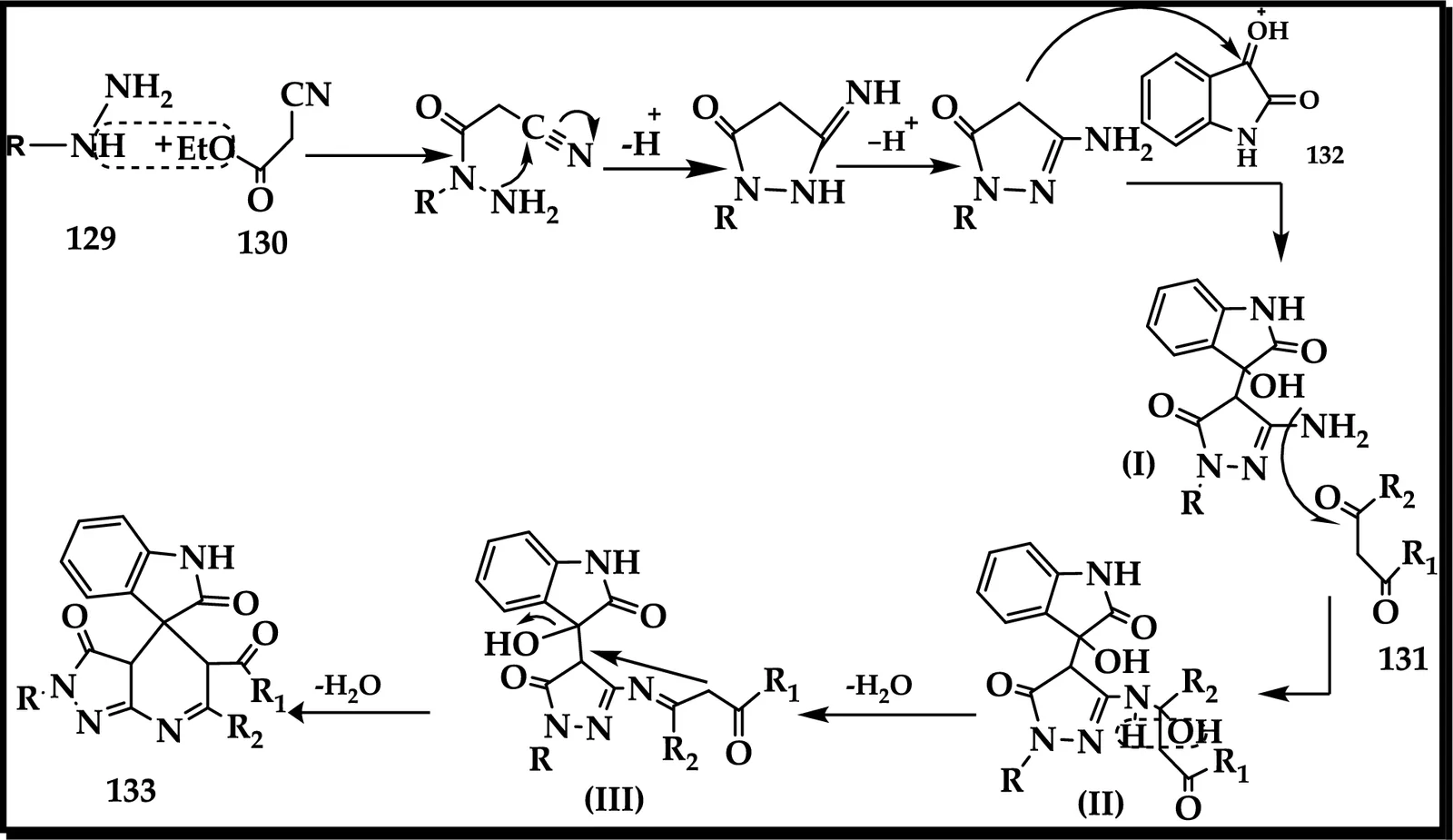
Mechanism for the synthesis of compound 133

The suggested mechanism of the reaction between phenyl hydrazine, ethyl cyanoacetate, and isatin is illustrated in Scheme 39.

## Reactions of pyrazolo[3,4-b] pyridine derivatives

### Reactions based on pyrazole moiety

**Scheme 40:**
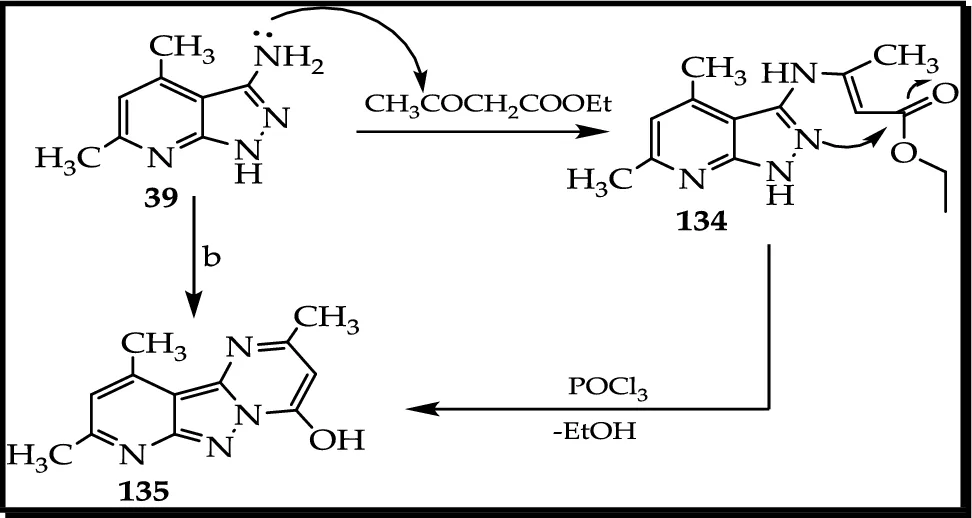
Reaction of 1-*H*-pyrazolo[3,4-*b*]pyridine with ethyl acetoacetate. Reagents and conditions: (a) ethyl acetoacetate, abs. EtOH, 1 h, 100 °C; (b) ethyl acetoacetate, abs. EtOH, conc. HCl, reflux, 40 min, 100 °C, (c) POCl_3_, reflux, 3 h, 150 °C

In 2022, Keshk [64] illustrated the reaction of dimethylpyrazolo[3,4-*b*]pyridin-3-amine **39** with ethylacetoacetate afforded 3-(4,6-dimethyl-2*H*-pyrazolo[3,4-*b*]pyridin-3-ylamino)-but-2-enoic acid ethyl ester **134**. Chlorination of compound **134** with POCl_3_ afforded pyridopyrazolopyrimidine **135**. The reaction mechanism for the formation of compound **135** involved the nucleophilic attack of the amino group of **39** on the carbonyl carbon of ethyl acetoacetate to afford **134** followed by nucleophilic attack of NH of pyrazole moiety on the ester carbonyl carbon to form compound **134** with elimination of ethanol (Scheme 40).

**Scheme 41:**
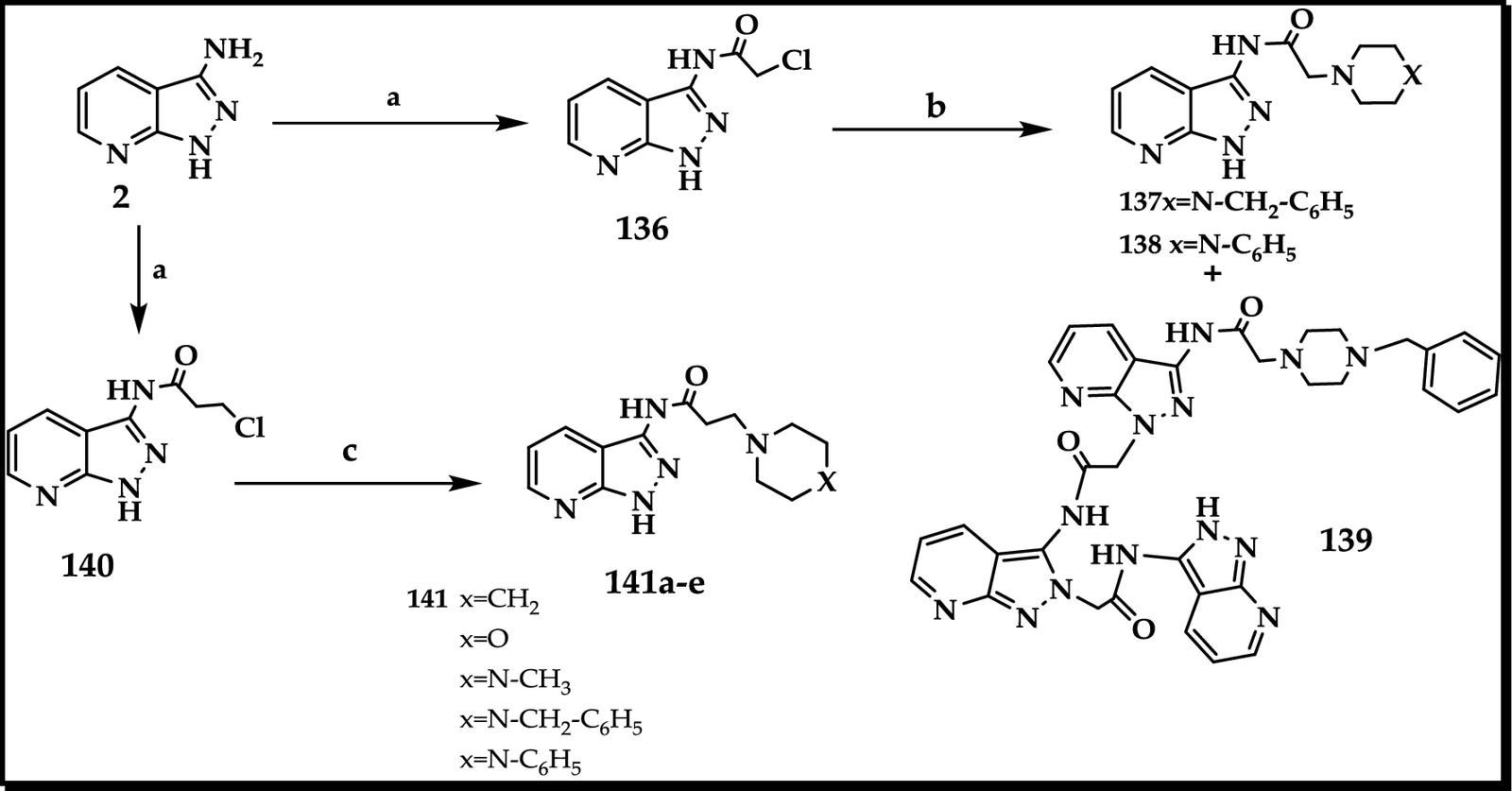
Acylation reaction of 3-amino of pyrazolo[3,4-*b*]pyridine. Reagents and conditions: (a) DMF; TEA, chloroacetyl chloride or chloropropionyl chloride, at 0 C, stirring at r. t, 8 h (60–78%); (b) = or phenylpiperazine; DMF, K_2_CO_3_, reflux, 12 h (25–55%); (c) piperidine, morpholine, methylpiperazine benzylpiperazine, or phenyl piperazine; acetonitrile or ethanol, K_2_CO_3_

Furthermore, the acylation reaction of 3-amino of pyrazolo[3,4-*b*]pyridine **2** with chloroacetyl chloride and chloropropionyl chloride afforded compounds **136** and **140**, respectively. Upon treating **136** with benzylpiperazine, an unusual dimeric product **139** was obtained in addition to the expected product **137** and** 138.** Finally, the reaction of compound **136** and **140** with different secondary cyclic amines produced compounds **137, 138**, and **141a-e,** respectively [19] (Scheme 41).

**Scheme 42:**
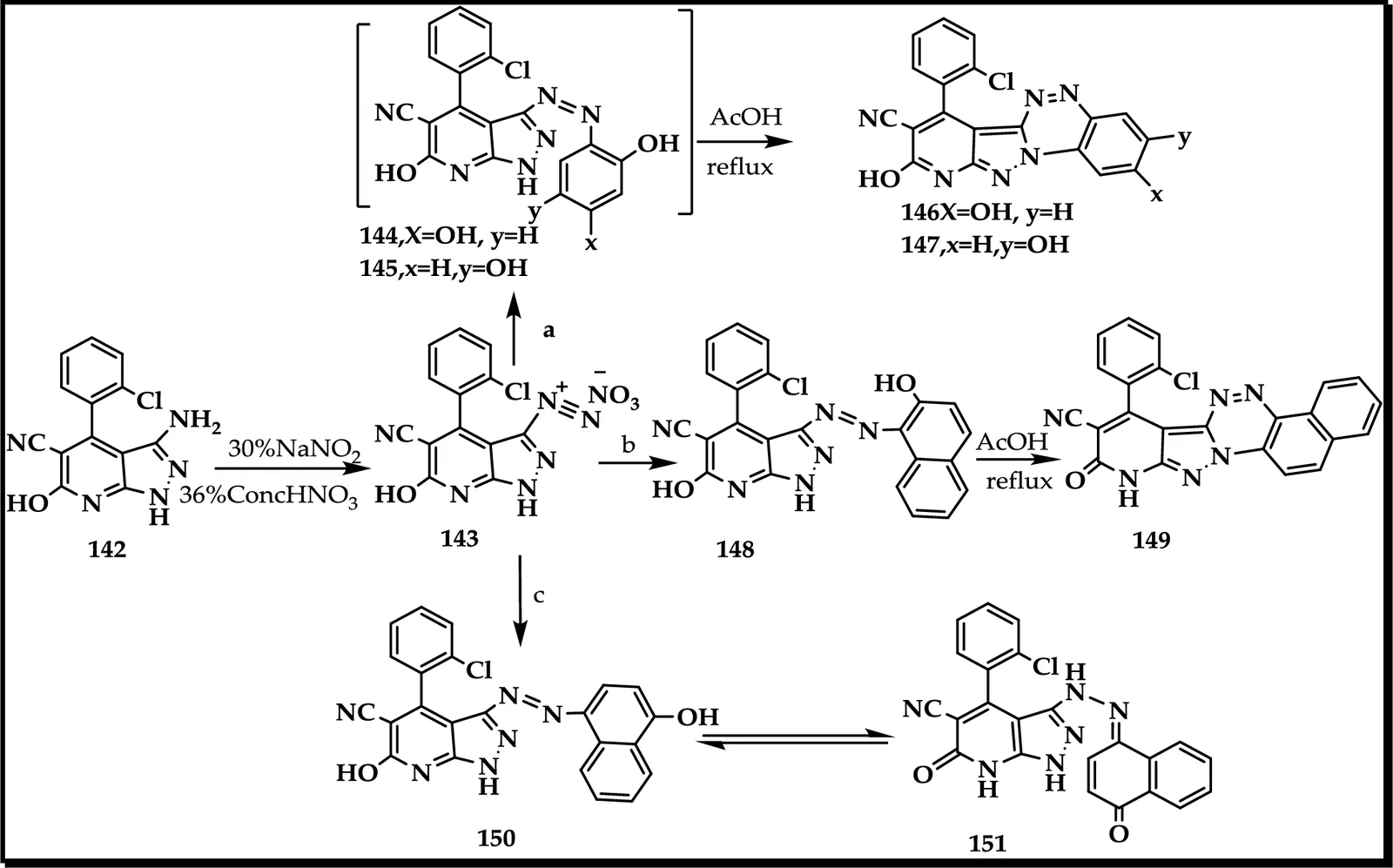
Diazotization reaction of aminopyrazolo[3,4-*b*]pyridine derivatives

Nafie et al*.* [41] reported the diazotization reaction of **142** with NaNO_2_/Conc HNO_3_ afforded the diazonium salt **143.** Coupling of **143** with resorcinol, *p-*dihydroquinone, *β*-naphthol, and α-naphthol the corresponding azo compounds **146-151,** respectively (Scheme 42).

**Scheme 43:**
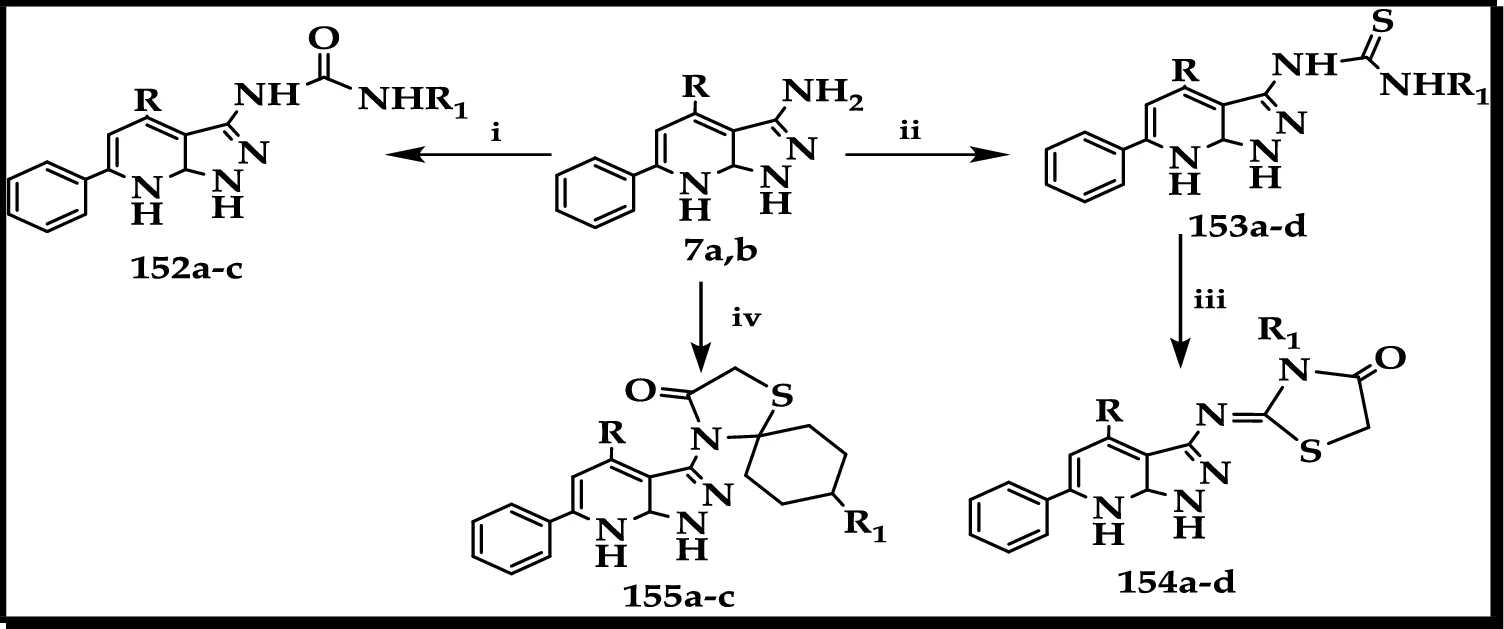
Reaction of 3-aminopyrazolo[3,4-*b*]pyridine derivatives 7a, b with alkyl or aryl (isocyanate/isothiocyanate) and cyclic ketones. Reagents and conditions: (i) alkyl or aryl isocyanates, EtOH, reflux for 6 h; (ii) alkyl or aryl isocyanates, EtOH, reflux for 8 h; (iii) ethyl chloroacetate, sodium acetate anhydrous, EtOH, reflux for 5 h; (iv) cyclohexanone or 4-methyl cyclohexanone, thioglycolic acid, dry benzene, reflux for 8 h

The reaction of 3-aminopyrazolo[3,4-*b*]pyridine derivatives **7a,b** with alkyl or aryl (isocyanate/isothiocyanate) and cyclic ketones in refluxed ethanol afforded the corresponding **152a-c**, **153a-d,** and **154a-c,** respectively. In addition, the reaction of compound **153a-d** with ethylchloroacetate afforded 3-alkyl/aryl-2((1*H-*pyrazolo[3,4-*b*]pyridin-3-yl)imino)thiazolidin-4-one derivatives **154a-d** [42] (Scheme 43).

**Scheme 44:**
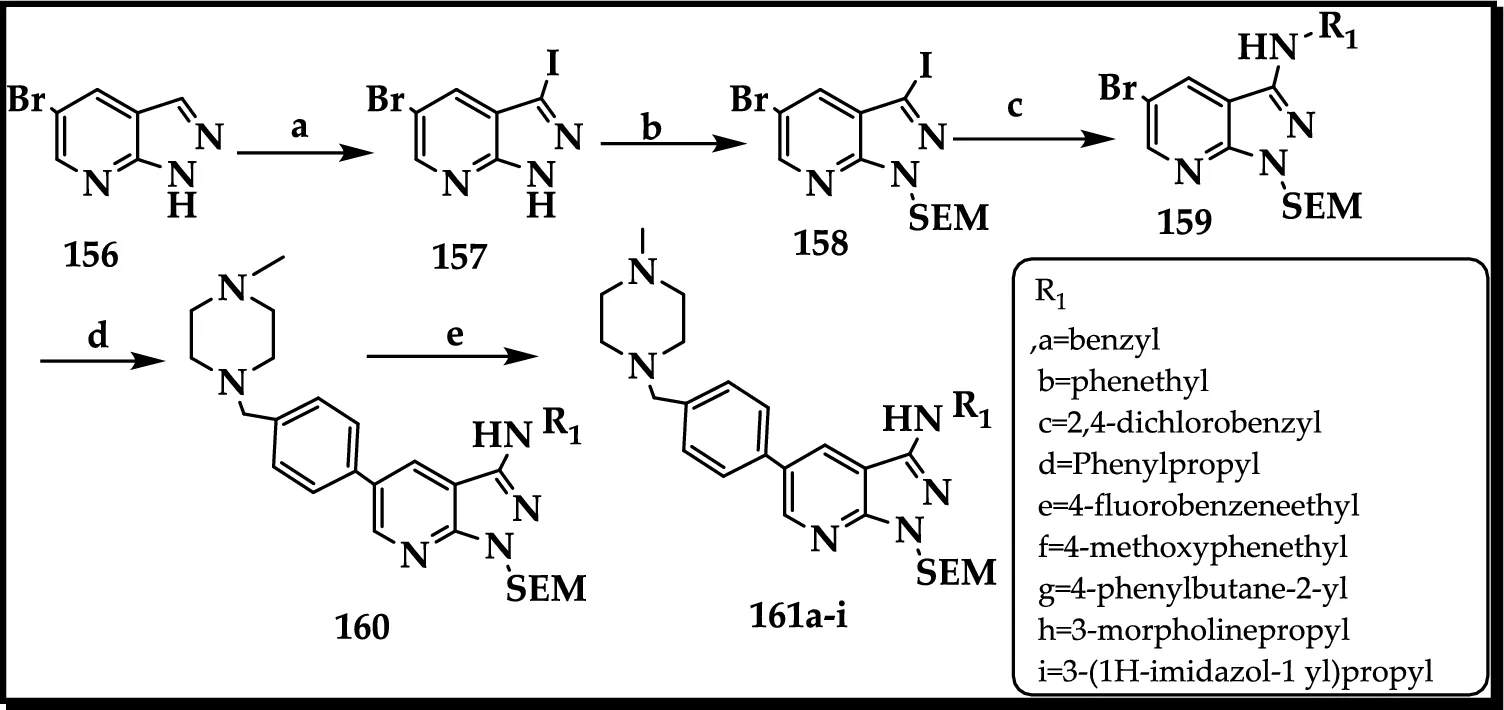
Reaction of 5-bromo-1*H*-pyrazolo [3,4-*b*]pyridine derivatives. Reagents and conditions: (a) N-Iodosuccinimide** (**NIS),DMF, 80 C, 8 h; (b) NaH, N(trimethyl) silylethoxymethyl (SEM-Cl), DMF, 0 C, r.t, 10 h; (c) Ar(CH_2_CH_2_)nNH_2_, t BuONa, 1,4-dioxane, 80 C, 10 h; (d) 1-Methyl-4-[4–(4,4,5,5-tetramethyl-1,3,2-dioxaborolan-2 yl) benzyl] piperazine, Pd(PPh_3_)_4_, Na_2_CO_3_, 1,4-dioxane: H_2_O 4: 1, 80C, 6 h; and (e) 4 M HCl in 1,4-dioxane, r.t.,4 h

Moreover, Sun et al*.* [65] prepared a series of the 3-substituted-1*H*-pyrazolo [3,4-*b*]pyridine derivatives **161a-i** via the routes outlined in Scheme 44.

### Reactions based on pyridine moiety

**Scheme 45:**
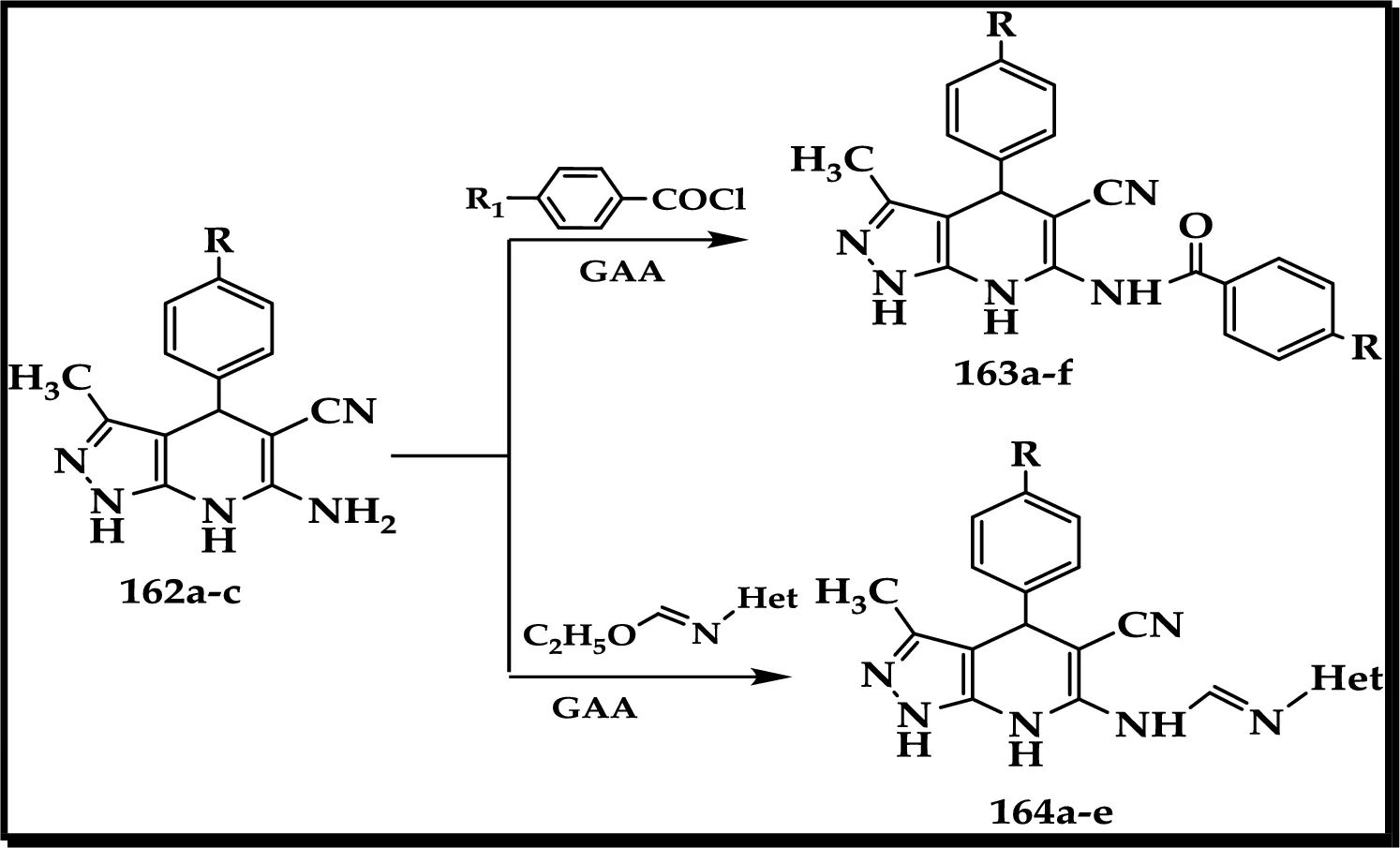
Arylation reaction of 1*H*-pyrazolo[3,4-*b*]pyridine-5-carbonitrile 162a-c

Hawas *et al*. [66] reported the reaction of 1*H*-pyrazolo[3,4-*b*]pyridine-5-carbonitrile **162 a-c** with benzoyl chloride or 4-nitrobenzoyl chloride and ethyl *N*-(heteroaryl)formimidates in glacial acetic acid afforded pyrazolo[3,4-*b*] pyridines **163a-f** and **164a-e**, respectively (Scheme 45).

**Scheme 46:**
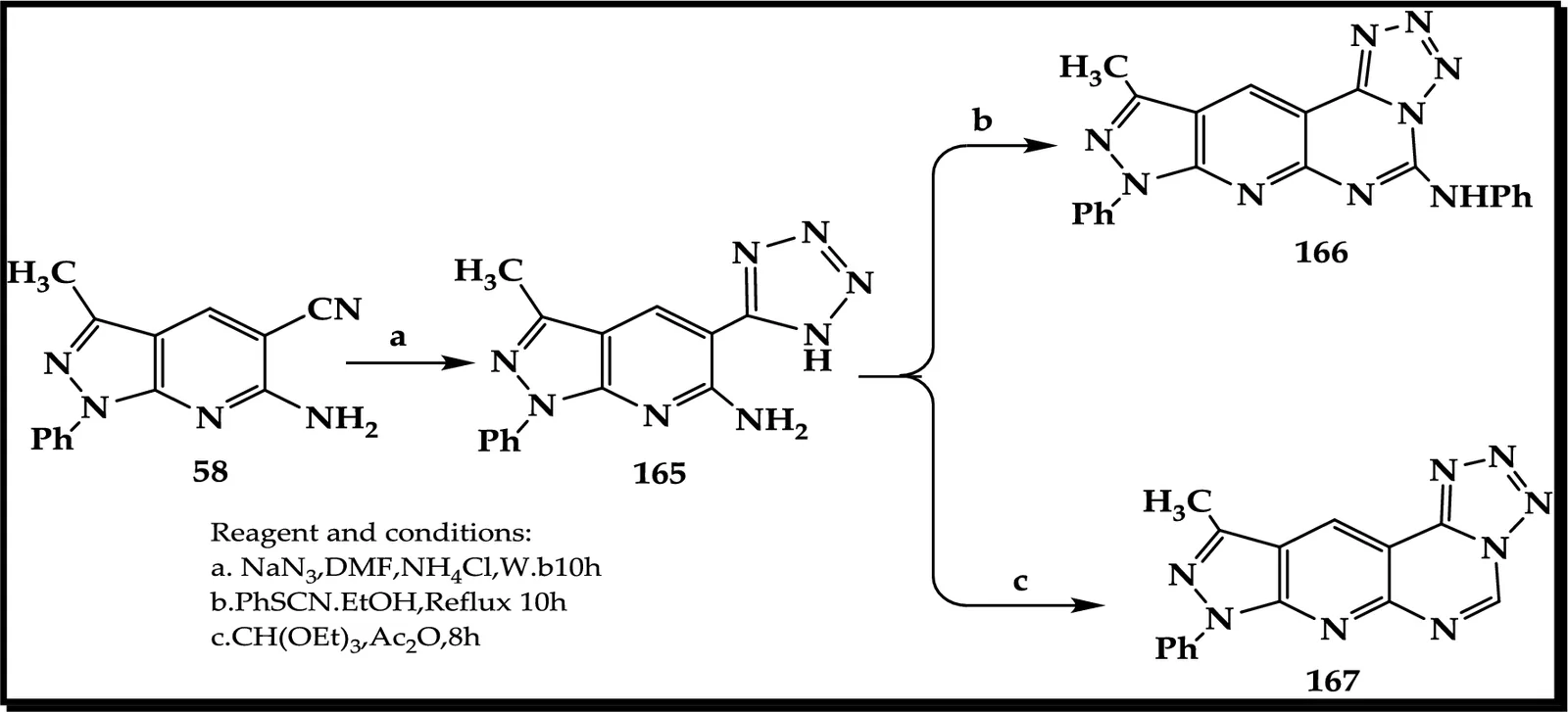
Reactions of 1*H*-pyrazolo[3,4-*b*]pyridine with different reagents

The reaction of 6-amino-3-methyl-1-phenyl-1*H*-pyrazolo[3,4-*b*]pyridine-5-carbonitrile **58** with sodium azide leads to the formation of tetrazolyl pyrazolo[3,4-*b*]pyridine derivative **165**. Treatment of compound **165** with phenyl isothiocyanate and/or triethyl orthoformate afforded pyrazolo-[4′, 3′, 5, 6] pyrido [3,2-*e*] tetrazolo [1,5-*c*]pyrimidines **166**and **167** [52] (Scheme 46).

**Scheme 47:**
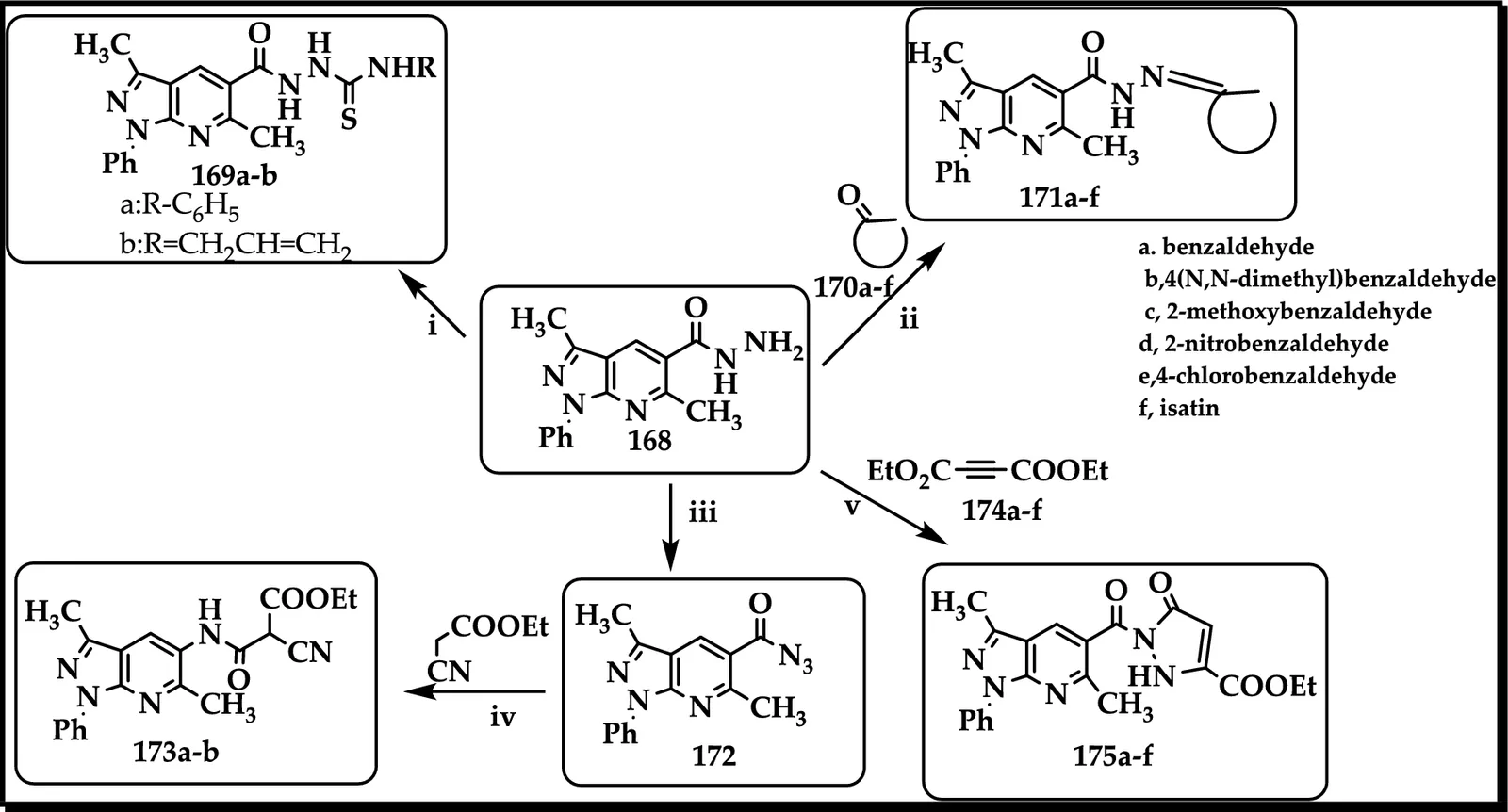
Different reactions on pyrazolo[3,4-*b*]pyridine 168. Reagents and conditions: (i) PhNCS (1**59a**) or CH_2_ = CHCH_2_NCS (1**59b**), EtOH, reflux 8–10 h; (ii) EtOH, piperidine, reflux 8–10 h; (iii) NaNO_2_/HCl, 0–5 °C; (iv) xylene/piperidine, reflux 12 h; (v) (vi) toluene, reflux 8 h

In 2019, Aly and his co-workers [67] reported the reaction of 6-dimethyl-1-phenyl-1*H*-pyrazolo[3,4-*b*]pyridine-5-carbohydrazide **168** with various reagents such as phenyl isothiocyanate, allyl isothiocyanate, various carbonyl compounds **170a-f**, ethyl cyanoacetate, and diethyl acetylene dicarboxylate **174a-f** to afford the corresponding pyrazolo[3,4-*b*]pyridine derivative as illustrated in Scheme 47.

**Scheme 48:**
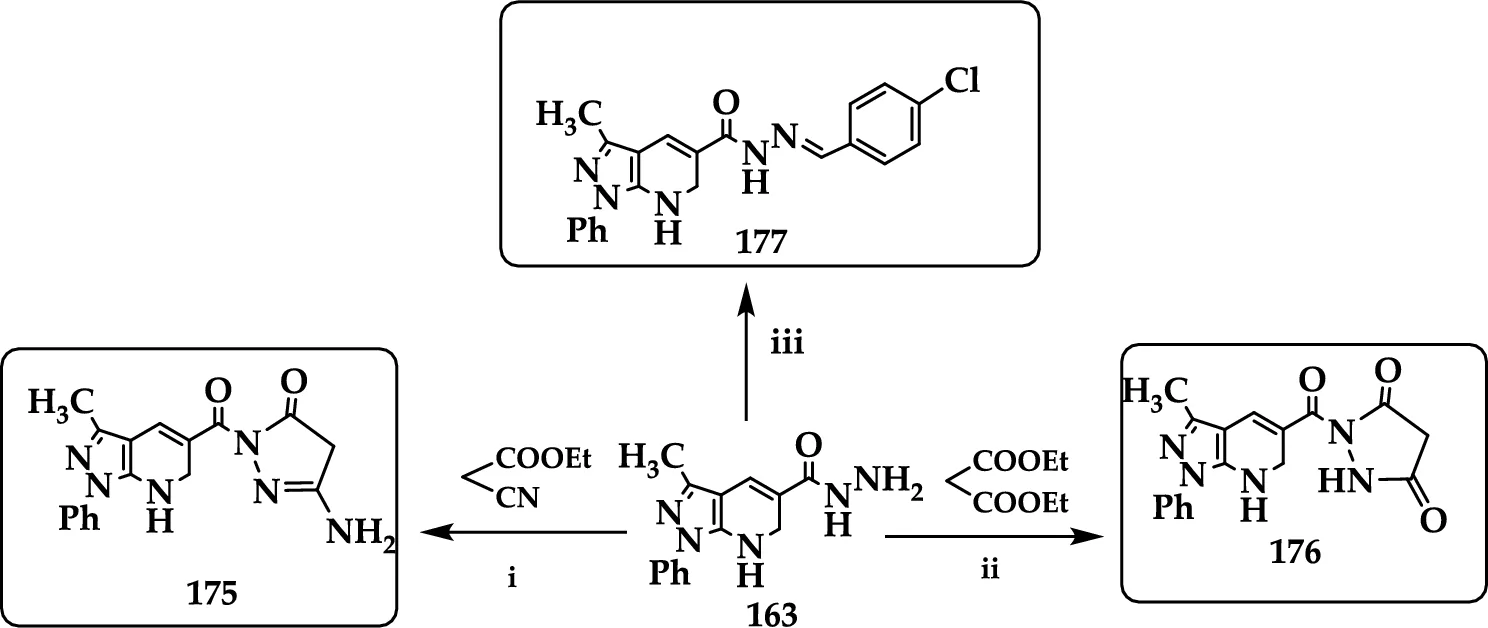
Reaction of pyrazolo[3,4-*b*]pyridne derivative 163 with active methylene compounds and aldehyde. Reagents and conditions: (i) AcOH/*H*_2_SO_4_, reflux, 10 h; (ii) 4-Cl-C_6_H_4_-CHO, EtOH/piperidine, reflux, 8 h

Furthermore, the reaction of pyrazolo[3,4-*b*]pyridine derivative **168** with active methylene such as ethylcyanoacetate and/or diethyl malonate in AcOH acid catalyzed by a few drops of conc. H_2_SO_4_ produced **175** and **176**, respectively**.** In addition, the reaction of equimolar amounts of the compound **163** and *p*-chlorobenzaldehyde in refluxing EtOH gave compound **177** in 90% yield [67] (Scheme 48).

**Scheme 49:**
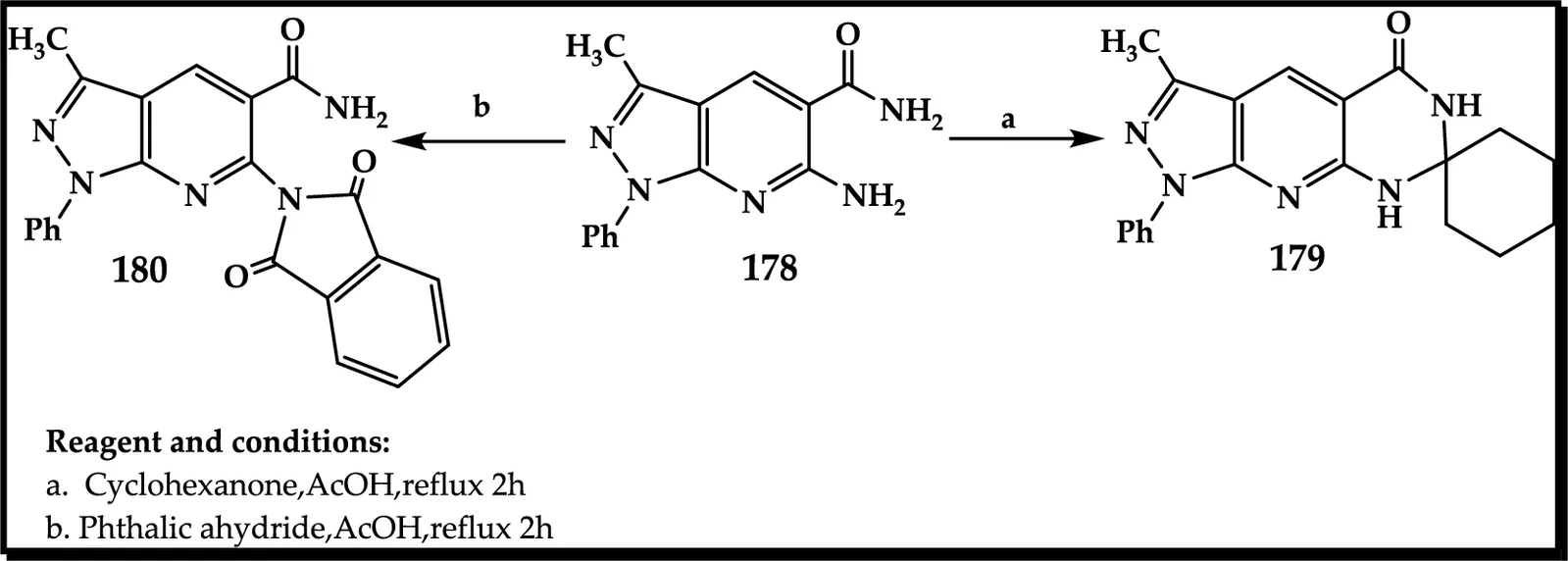
condensation reaction of pyrazolo [3,4-*b*] pyridine derivatives 178. Reagent and conditions: a-Cyclohexanone,AcOH,reflux 2h, b- Phthalic ahydride,AcOH,reflux 2h

On the other side, various 1*H*-pyrazolo [3,4-*b*]pyridine derivatives **179** and **180** were obtained by condensation reaction of pyrazolo [3,4-*b*] pyridine derivatives **178** with cyclohexanone or phthalic anhydride in acetic acid [52] (Scheme 49).

**Scheme 50:**
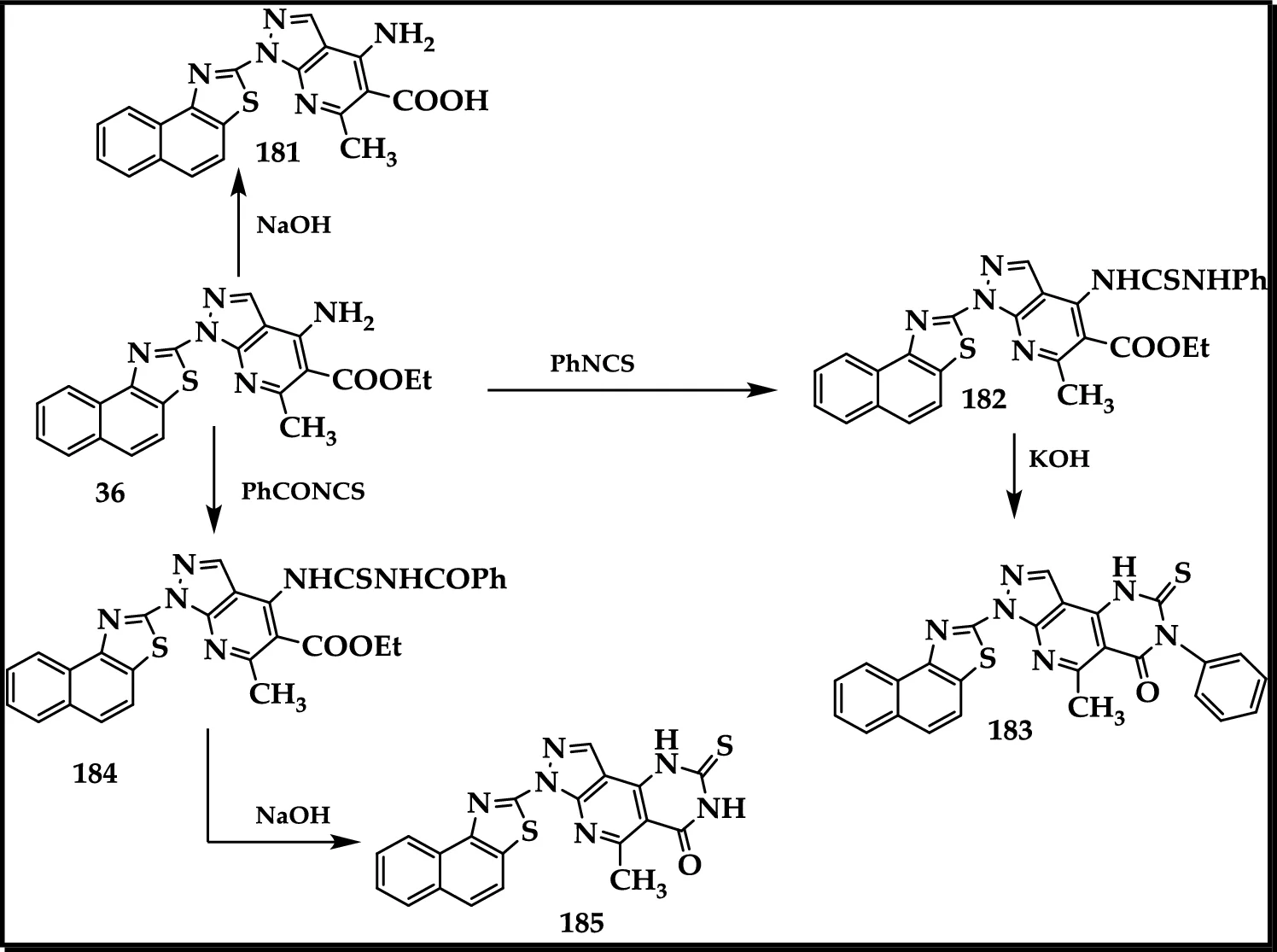
Reactions on compound 36

Fayed et al. [47] found that the hydrolysis of amino ester of 1*H*-pyrazolo[3,4-*b*]pyridine **36** led to the formation of amino acid **181.** In addition, the reaction of compound **36** with phenyl isothiocyanate and benzoyl isothiocyanate yielded the thiourea and benzoyl thiourea derivative **182** and **184** derivatives^.^ Heating compounds **182** and **184** with alkali lead to cyclization afforded **183** and **185**, respectively (Scheme 50).

### Biological evaluation of 1*H*-pyrazolo[3,4-*b*]pyridine

Among the various heterocyclic molecules employed for drug design and discovery, pyrazolopyridine is one of the promising pharmacophores. This review is aimed to discuss the pharmacological properties in brief with structure activity relationship (SAR). The important parts of the overall biomedical applications for 1*H*-pyrazolo[3,4-*b*] pyridines are anticancer, antimicrobial, anti-inflammatory, anti-Alzheimer, and antiviral activity.

### Antitumor activity

A type of anticancer drug that stops or slowing the abnormal growth of tissue by interfering with DNA, the genetic material in cells. In this way, drug design is a part of drug discovery and development. Studies indicated that heterocycles have favorable interactions with protein targets of different diseases. Furthermore, fused heterocycles have been demonstrated as remarkable anticancer agents. Among them, the pyrazolo[3,4-*b*] pyridines were identified as anticancer agents with a broad spectrum of action.

**Fig. 6:**
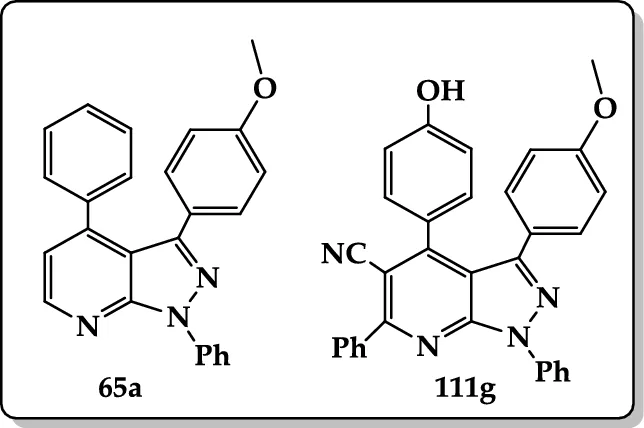
Compounds with anti-cancer activity against Hela, MCF7, and HCT-116

1*H* pyrazolo[3,4-*b*]pyridine derivatives **65a-h** [53] and **111a-h** [1] were synthesized and assessed for anticancer activity against Hela, MCF7, and HCT-116 cancer cell lines compared with doxorubicin as a reference compound. They were shown the highest anticancer activity. The cytotoxicity of compound **65a** (IC_50_ = 26.44 ± 3.23 µM) and **111 g** (IC_50_ = 21.81 ± 2.96 µM) showed good safety profiles on normal cell line **WI-38** when compared with doxorubicin (IC_50_ = 15.60 ± 0.37 µM). SAR studies showed that good anticancer activity of compound **65a** related to *para*-unsubstituted phenyl with respect to Hela cell line. While Compound **111 g**, with OH group in the phenyl group of position **4** in pyridine moiety, in addition to the Ph group in position **2** and the CN function in position **3** of the pyridine moiety, enhanced its anticancer activity toward MCF7 and HCT-116 cell lines (Fig. 6).

**Fig. 7:**
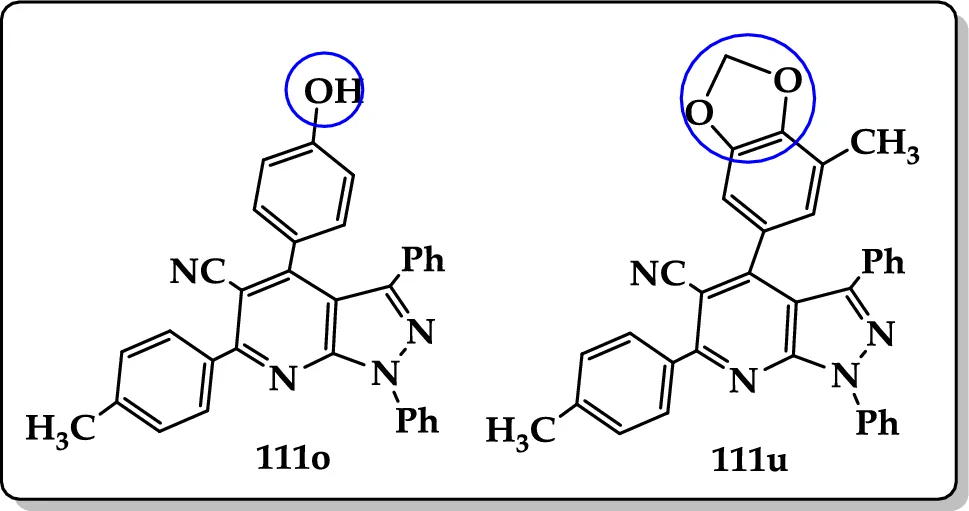
Compound 11 showed anti-proliferative activity against leukemia cell lines

Barghash et al. [7] designed and synthesized new pyrazolo[3,4-*b*]pyridine derivatives **111 o, u** and investigated their antiproliferative activity against leukemia cell lines (K562, MV4-11, CEM, RS4;11, ML-2, and KOPN-8). The results showed that they possessed excellent antileukemic activity due to the presence of a hydroxyl or methylenedioxy group within C-4-phenyl moiety (Fig. 7).

**Fig. 8:**
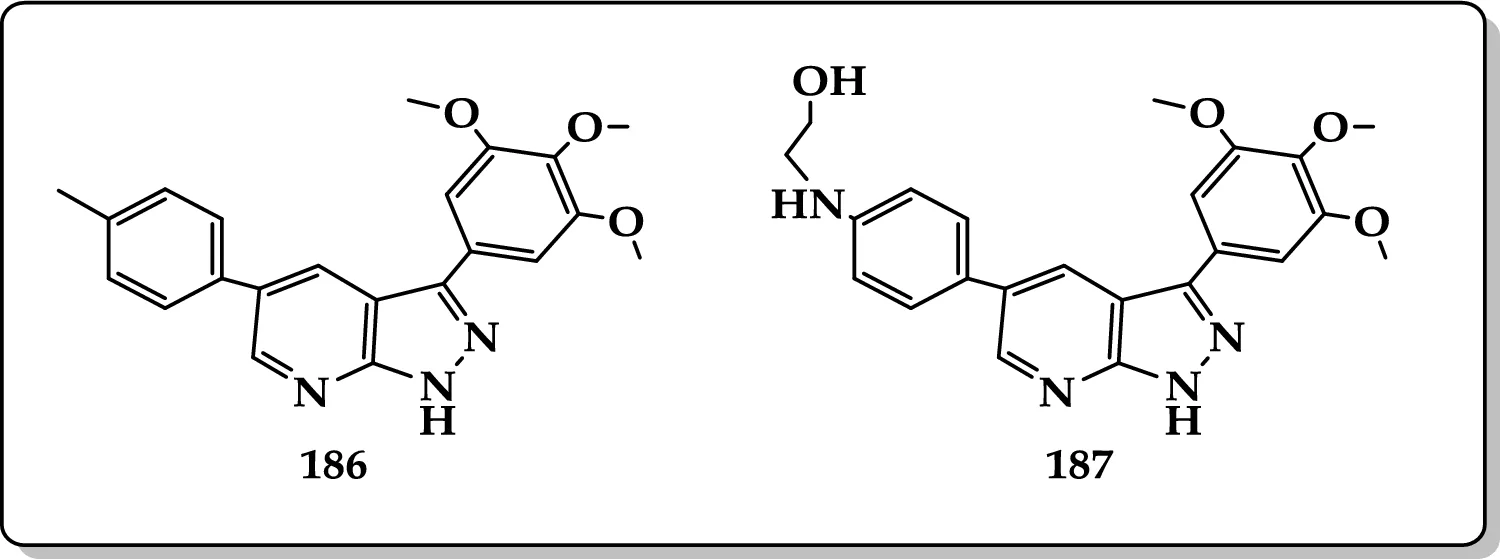
1*H*-pyrazolo[3,4-*b*] with anti-proliferative activity

1*H*-Pyrazolo[3,4-*b*]pyridine derivatives [46] **186** has been designed as a tubulin polymerization inhibitor targeting the colchicine site. It has been tested by MTT assays for its antiproliferative activity against SGC-7901 (human gastric), A549 (human lung), and HeLa (Human cervical) carcinoma cell line. The compound **186** exhibits noticeable in vitro potential activity because SGC-7901 has an IC_50_ = 13 nM compared with SMART IC50 = 18 nM as positive control, and it could significantly inhibit tubulin polymerization and strongly disrupt the cytoskeleton. SAR results showed that this activity is due to the present donating methyl group at position **4** of phenyl ring (Fig. 8).

In addition, 3-(3,4,5-trimethoxyphenyl)-1*H*-pyrazolo[3,4-*b*]pyridine **187** was prepared and investigated for antiproliferative activities against human cancer cell lines including colon (HT-29), leukemic (MOLT-4), non-small-cell lung (A549), breast (MCF7 and MDA-MB-231), liver (PLC8024), and pancreatic (PANC-1) cancer cell and It showed strong potency against all cancer cell lines, with an average IC50 value of 58.609 nM [22] (Fig. 8).

**Fig. 9:**
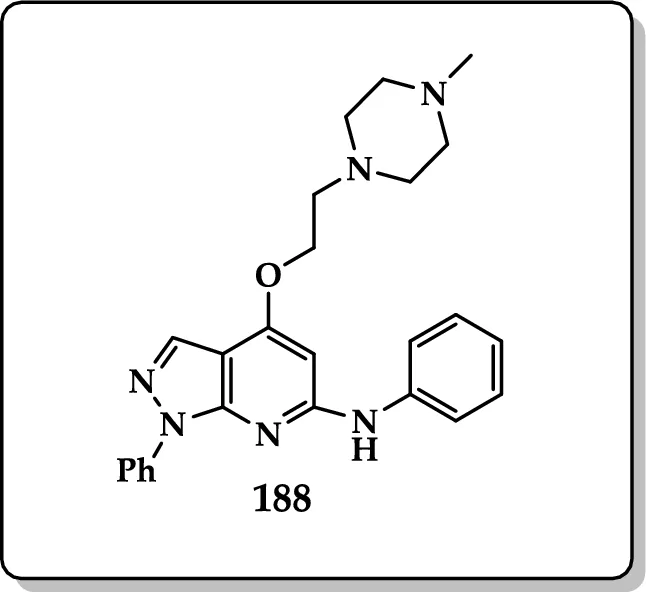
1*H*-pyrazolo[3,4-*b*] pyridine with anticancer activity

Also, Georgiou et al. [54] synthesized new 4-(4-methylpiperazin-1-yl)ethoxy pyrazolo[3,4-*b*]pyridine derivative **188** and investigated for their antitumor activity. Compound **188** displayed better antitumor on tumor cells and weak toxicity to normal cells **WI-38**, where SAR showed that the higher activity was due to the presence of 4-methylpiperazin within C-4 pyridine moiety (Fig. 9).

**Fig. 10:**
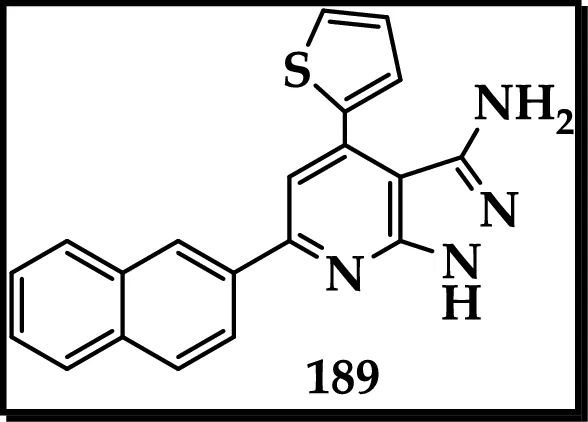
1*H*-pyrazolo[3,4-*b*] pyridine with high inhibitory activity against MCF-7

Furthermore, a new series of 1*H*--pyrazolo[3,4-*b*] pyridines **189** were designed, synthesized, and evaluated for their anticancer activity against MCF7, HepG2, and A549 human lung cancer cell. Compound **189** exhibited higher inhibitory activity (IC_50_ = 19.3 ± 2.1) compared to doxorubicin (IC_50_ = 64.8 µM) against MCF7 due to the presence of the thiophene ring according to structural activity relationship [12] (Fig. 10).

### Antimicrobial activity

Antimicrobial is an agent that kills microorganisms (microbicide) or stops their growth (bacteriostatic agent). Antimicrobial medicines can be grouped according to the microorganisms they act primarily against. For example, antibiotics are used against bacteria, and antifungals are used against fungi.

**Fig. 11:**
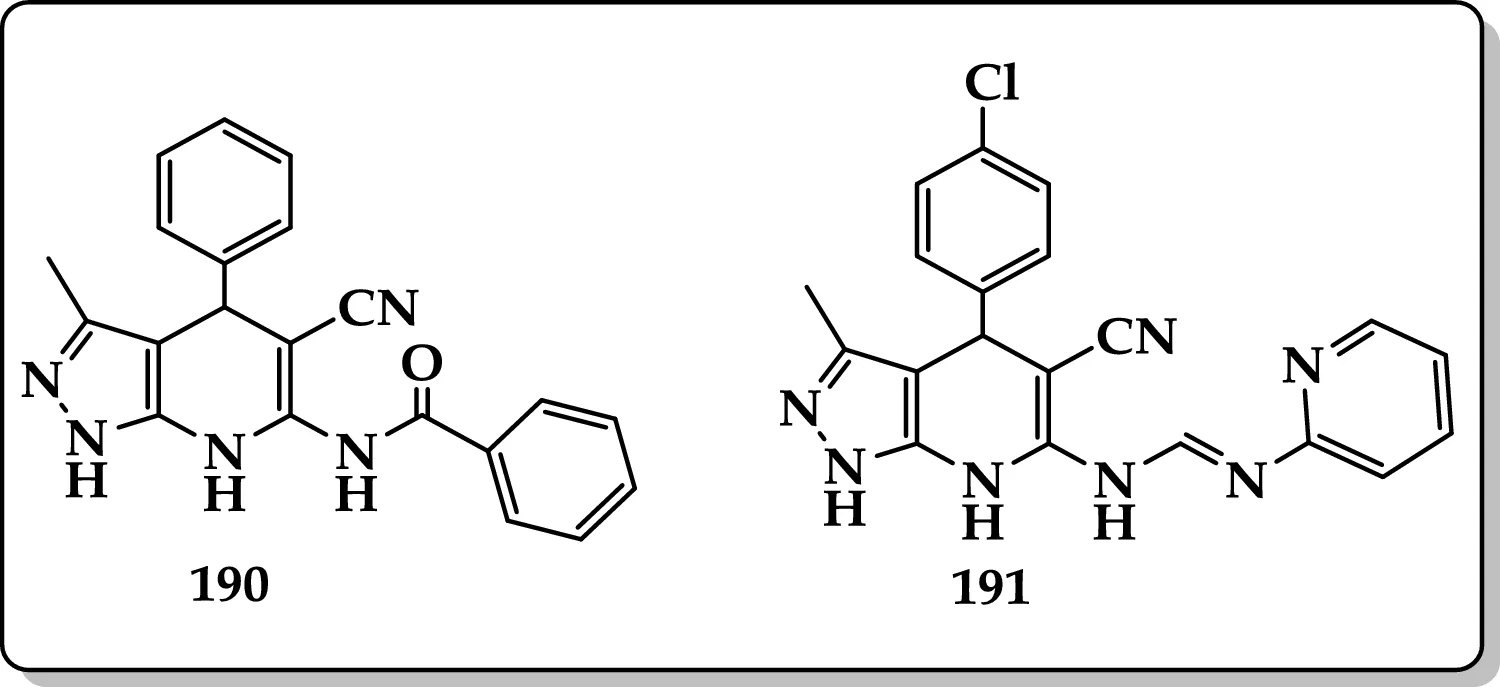
1*H*-pyrazolo[3,4-*b*]pyridine with high antimicrobial activity

Hawas et al. [66] designed and prepared a new 1*H*-pyrazolo[3,4-*b*]pyridine derivatives **190** and **191** and tested as antimicrobial agents against Gram-positive bacteria (*S. aureus* and *B. cereus*), Gram-negative bacteria (*E. coli* and *P. aeruginosa)*, and fungi(*C. albicans* and *A. fumigatus*). Ampicillin (antibacterial) and fluconazole (antifungal) were employed as reference drugs. The compounds **190** and** 191** showed high antimicrobial effectiveness on all tested strains and might be regarded as effective antimicrobial agents with reduced danger of antimicrobial resistance (AMR). SAR showed the highest activity of compounds **190** and **191** due to the presence of 4-chlorophenyl and 4-phenyl within C-4 pyridine moiety (Fig. 11).

**Fig. 12:**
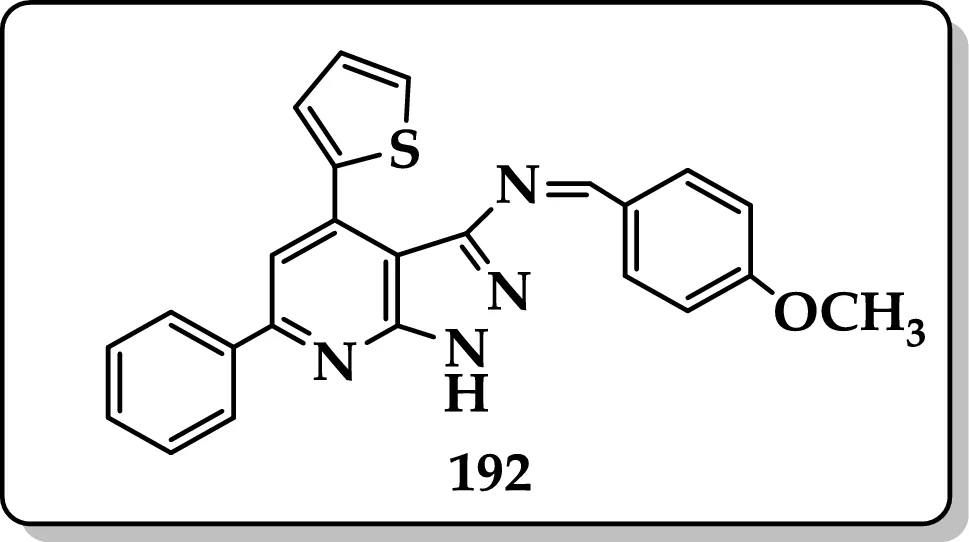
Compound 192 showed antimicrobial activity and enzyme dihydrofolate reductase (DHFR)

On the other hand, 1*H*-pyrazolo[3,4-*b*]pyridine derivatives [42] **183** were synthesized and evaluated as antimicrobial agents against several bacterial, fungal strains, (MRSA) and enzyme dihydrofolate reductase (DHFR). The results demonstrated that compound **192** bearing (4-methoxyphenyl) methanimine has the highest inhibition zone against the resistant bacteria methicillin-resistant *Staphylococcus aureus* (MRSA) with (MIC = 4 µg/m) compared with Gentamicin (MIC = 8 µg/m) as a reference drug. Also, it showed the inhibitory activity against dihydrofolate reductase (DHFR) with IC_50_ values 0.72 ± 0.04 µM, compared with the IC_50_ value 5.54 ± 0.31 µM of the reference inhibitor (Trimethoprim) (Fig. 12).

**Fig. 13:**
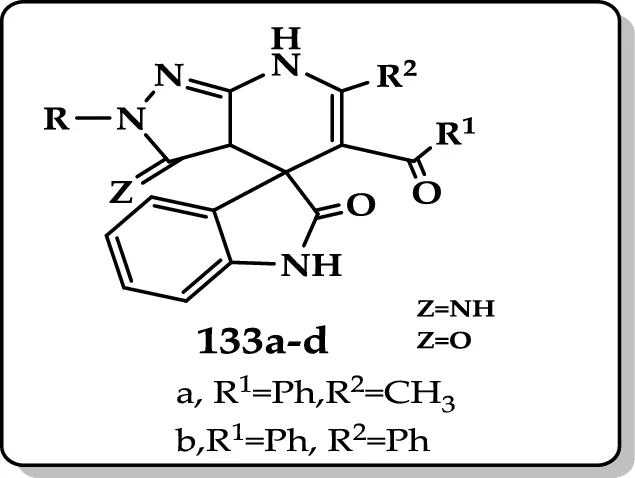
Spiro[indoline-3,4′-pyrazolo[3,4-*b*] pyridines with high antimicrobial activity

Naglaa et al. [63] obtained spiro[indoline-3,4′-pyrazolo[3,4-*b*] pyridines **133a,b** and investigated their antimicrobial activity against bacterial strains *S. aureus* (G + ve), *P. aeruginosa* (G -ve) as well as two different fungi: *C. albicans* (yeast) and *A. niger* (fungus). The results showed that they had good potential activity which suggests that those compounds might have promising chances to be effective antimicrobial curative and potencies (Fig. 13).

**Fig. 14:**
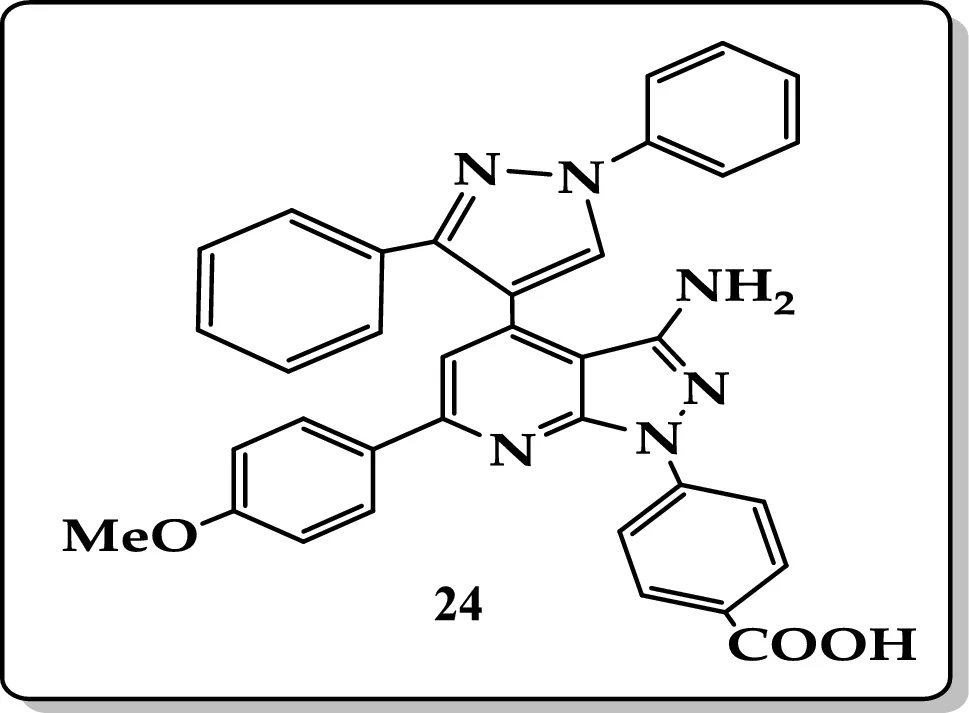
Compound 24 showed strong antibacterial activity

Also, pyrazolo[3,4-*b*]pyridine-3-amine derivatives [44] **24** were obtained and evaluated for antibacterial activity against two Gram-positive bacterial strains (*S. aureus* and *S. mutans*) and two Gram-negative bacterial strains (*K. pneumonia* and *E. coli*). The results was showed strong antibacterial activity of compound **193** with inhibition zones of 42.0 ± 0.6 and 35.4 ± 0.6 mm and MIC values of 62.5 and 125 μg/mL against *S. aureus* and *S. mutans*, respectively, compared to standard drug ampicillin 22 ± 0.1, 28 ± 0.5, and 125 and 250 μg/m. The SAR studies showed this activity due to the presence of carboxylic group (Fig. 14).

**Fig. 15:**
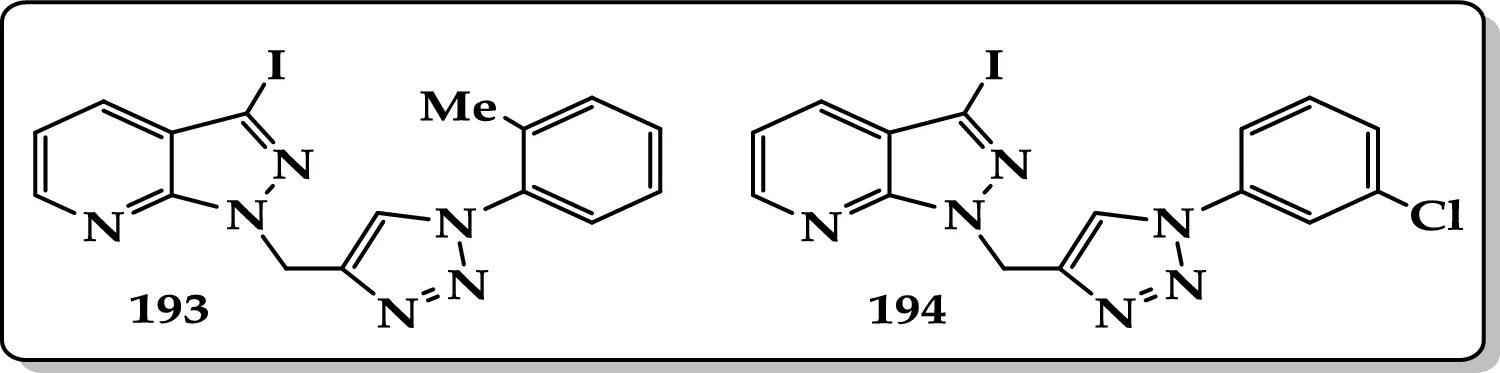
1*H*-Pyazolo[3,4-*b*]pyridine with strong antibacterial activity.

Moreover, 1*H*-pyazolo[3,4-*b*]pyridine [15] **193** and **194** were synthesized and tested against bacterial strains *S. aureus* as Gram-positive bacteria and *K. pneumoniae* as Gram-negative bacteria. They demonstrated strong antibacterial activity with inhibition zone values 15 ± 0.82, 14 ± 0.7 mm, and 18 ± 0.95, 16 ± 0.82 mm. MIC and MIB values were found to be 0.25, 0.5 μg/mL and 0.25, 0.5 μg/mL against *S. aureus* and *K. pneumonia,* respectively. This higher activity is due to the presence methyl group at the 2nd position of the phenyl ring of compound **193** and chloro atom at the 3rd position of the phenyl ring of compound **194** (Fig. 15).

### Anti-inflammatory activity

**Fig. 16:**
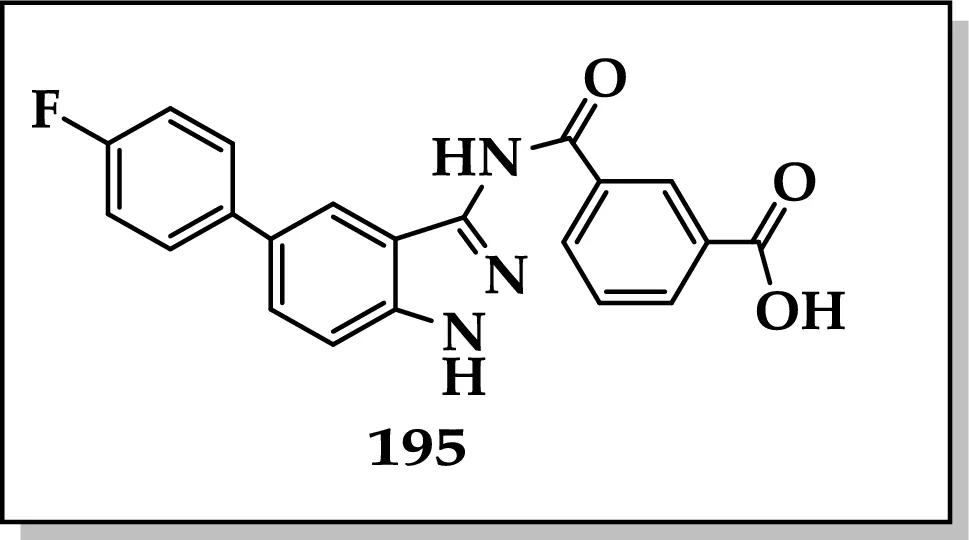
1*H*-Pyazolo[3,4-*b*]pyridine with strong antibacterial activity

Anti-inflammatory agents block certain substances in the body that cause inflammation. They are used to treat many different conditions. Some anti-inflammatory agents are being studied in the prevention and treatment of cancer. The inhibition of spleen tyrosine kinase (Syk) is a motivating approach for the treatment of several allergic and autoimmune disorders, such as rheumatoid arthritis, asthma, and allergic rhinitis. Compound **195** presents great Syk inhibitory activity IC_50_ = 1.2 μM as compared with a reference (IC_50_ = 3.2 µM). The results revealed that the introduction of meta-benzoic acid **195** led to significant increase of the Syk inhibitory activity [68] (Fig. 16).

### Anti-Alzheimer activity

Alzheimer’s disease (AD) is the most common neurodegenerative disorder. In the brain with (AD), there are lower levels of acetylcholine (*Ach*) which helps to send messages between certain nerve cells. In (AD), there is also a loss of the nerve cells that use (Ach). As a result, falling (*ACh*) levels and progressive loss of these nerve cells are linked to worsening symptoms. Donepezil, rivastigmine, and galantamine all prevent an enzyme called acetylcholinesterase (*AChE*) and butyryl cholinesterase (*BuChE*) from breaking down (*ACh*) in the brain. As a result, an increased concentration of (*ACh*) leads to increased communication between nerve cells. This may temporarily alleviate or stabilize some symptoms of Alzheimer’s disease.

**Fig. 17:**
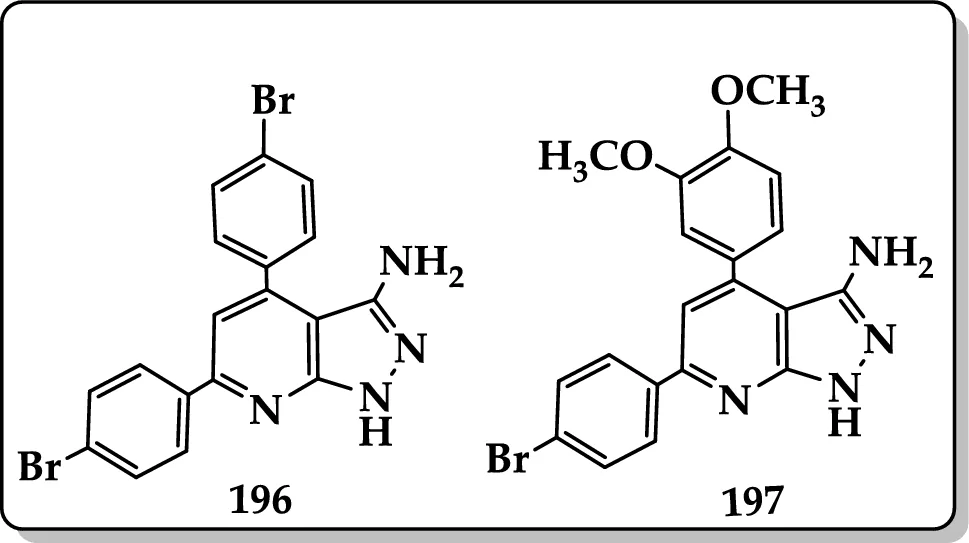
1*H*-pyrazolo[3,4-*b*] pyridines with hAChE and hBuChE enzyme inhibition

Waly et al*.* [20] synthesized 6-phenyl-1*H*-pyrazolo[3,4-*b*]pyridin-3-amine derivatives **196** and **197** and assessed for *h*AChE and *h*BuChE enzyme inhibition. They showed remarkable activity with IC50 values of 0.17 and 0.16 μM compared to donepezil and rivastigmine IC_50_ = 0.219 and 1.32 μM as reference drug, respectively. This higher activity is due to the presence Br atom at position **4** of the phenyl ring of compound **196** and 3,4-dimethoxy groups at position **3** and **4** of the phenyl ring of compound **197**, respectively. So, they could be used for further development of new multitargeted anti-Alzheimer’s agents (Fig. 17).

**Fig. 18:**
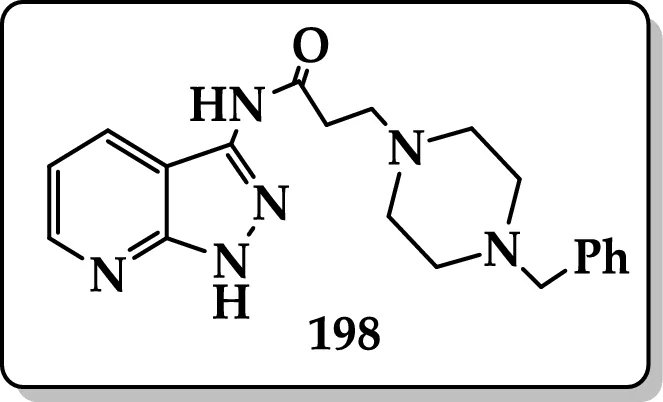
1*H*-pyrazolo[3,4-*b*] pyridine derivative with anti-Alzheimer activity

1*H*-pyrazolo[3,4-*b*] pyridine derivatives **198** was prepared and proved to be anti-Alzheimer’s disease (AD) agents by Ellman’s method. Where they showed potent *hAChE* and* h*BuChE inhibitors with IC50 = 0.034, 0.062 mM and IC_50_ = 3.567, 0.767 mM compared to donepezil and rivastigmine IC_50_ = 0.054, 4.450 mM, respectively. Compound **198** was found to be twofold more active than donepezil. SAR studies demonstrated that compound **198** was a good active *hAChE* inhibitor owing to the presence of an *N*-benzylpiperazine substituent [18] (Fig. 18).

### Antiviral activity

An antiviral is a substance that fights against viruses and inhibits their growth but not effective against bacteria or fungi. Antiviral products used to prevent the growth and spread of viruses. Any substance that slows the replication of viruses is an antiviral agent.

**Fig. 19:**
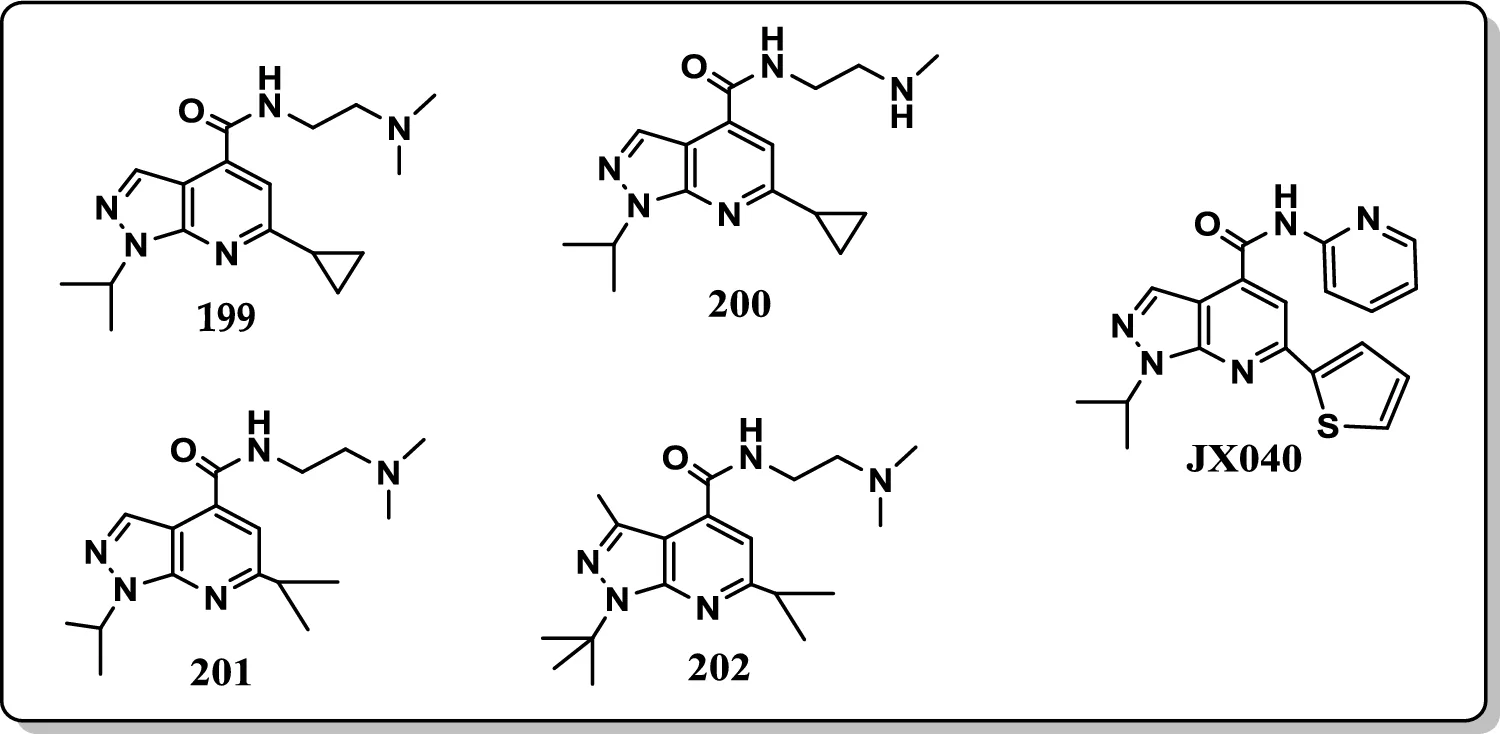
Pyrazolo[3,4-*b*]pyridine derivatives with antiviral activity

Hu et al. [21] synthesized a series of pyrazolo[3,4-*b*]pyridine derivatives and examined for their antiviral activity against nonpolio enteroviruses including EV-D68, EV-A71, and CVB3. Among the tested compounds, **199-202** were the most potent antiviral agents against EV-D68 and EV-A71 with a high cellular selectivity index with EC_50_ values of 0.03, 0.07, 0.03, and 0.34 μM, compared to **JX040 (**EC_50_ = 0.2) as antiviral drugs (Fig. 19).

## Conclusion

Pyrazolopyridine derivatives have shown remarkable potential against a variety of biological targets, making them viable options for further therapeutic development. This review provides a detailed information of recent advancements in pyrazolo[3,4-*b*]pyridine synthesis, reactions, and application. Modern synthetic techniques have not only made building these frameworks more effective and selective, but they have also created new opportunities for the quick creation of structurally varied libraries of derivatives. To fully realize the potential of these molecules, more research and development of reaction strategies, such as greener procedures, catalytic systems, and late-stage functionalization techniques, will be necessary. The advancement of pyrazolo[3,4-*b*]pyridine chemistry and the optimization of its influence on materials science and medicine will require interdisciplinary research that integrates synthetic chemistry, computational modeling, and biological evaluation. Therefore, it seems that pyrazolopyridine derivatives have a bright future ahead of them, with chances to play a major role in the development of new medicines and functional materials. However, the progress in pyrazolo[3,4-b]pyridine synthesis holds great potential for future advancements and applications in drug discovery.

## Data Availability

No datasets were generated or analyzed during the current study.
